# Assessing the water quality hazard and challenges to achieving the freshwater goal in Sri Lanka

**DOI:** 10.1038/s41598-025-93845-1

**Published:** 2025-03-25

**Authors:** Mohammad Shamsudduha, Jaeyoung Lee, George Joseph, Aroha Bahuguna, Samantha Wijesundera, Sreeshankar S. Nair, Yi R. Hoo, Qiao Wang, Sophie C. E. Ayling

**Affiliations:** 1 Department of Risk and Disaster Reduction, University College London, London, UK; 2 Centre for Resilience in Environment, Water and Waste, University of Exeter, Exeter, UK; 3 The World Bank, Washington, DC USA; 4 The World Bank, Colombo, Sri Lanka; 5 The Bartlett Centre for Advanced Spatial Analysis, University College London, London, UK

**Keywords:** Environmental sciences, Hydrology, Natural hazards

## Abstract

Nearly 93% of all drinking water supply in Sri Lanka comes from improved sources such as tubewells. Despite this national achievement, deteriorating water quality remains a major challenge to achieving the UN Sustainable Development Goals, particularly SDG 6 (‘Clean Water and Sanitation’). In this study, we analyze the state of ambient water quality at the national scale using observational and gridded datasets for both groundwater and surface water quality. We collated single-point, one-off measurements from 1,252 groundwater quality datasets provided by two national agencies in Sri Lanka: the National Water Supply and Drainage Board (NWSDB) and the Water Resources Board (WRB), as no national-scale time-series database on groundwater quality exists. Applying geospatial mapping techniques, we developed a multi-parameter (i.e., chloride, alkalinity, nitrate, nitrite, fluoride, phosphate, total dissolved solids, hardness, iron, and sulphate) groundwater-quality hazard map of Sri Lanka. Our results indicate that 3.6 to 3.8 million people are exposed to poor quality of groundwater that is used primarily for drinking purpose. We also find that surface water quality in some river basins (e.g., Kelani River) is compromised due to contamination from industrial and agricultural activities. We conclude that poor water quality, coupled with a lack of strategic national-scale monitoring system for routine water-quality measurements of both surface water and groundwater, pose a critical barrier to achieving sustainable drinking water supply in Sri Lanka.

**Supplementary Information:**

The online version contains supplementary material available at 10.1038/s41598-025-93845-1.

## Introduction

Good quality water for drinking and domestic use, whether it comes from surface or groundwater sources, is beneficial for human health and wellbeing^1^. Access to good-quality water, together with access to adequate water-supply, is necessary for achieving the UN Sustainable Development Goals (SDGs) for food security (SDG 2), human health (SDG 3), and clean water (SDG 6) around the world. A recent report based on a scientific study of global water quality^2,3^reveals that rich and poor countries alike endure high levels of degraded water quality. Like many other developing nations in the Global South, Sri Lanka is committed^4^ to achieving, among others, SDG 6 (*clean water and sanitation for all by 2030*) – also known as the Water Goal. The provision of safe drinking water is one of the highest priorities of the Government of Sri Lanka and, periodically, targets are set to provide improved access to safe drinking water and adequate sanitation^5^. Despite the widespread coverage of ‘improved’ drinking water supplies (e.g., tubewells, piped water supply) in Sri Lanka, challenges remain to achieving the water goal due to degraded surface and groundwater sources and a lack of monitoring systems for routinely measuring water quality and storage changes over time.

**Fig. 1:**
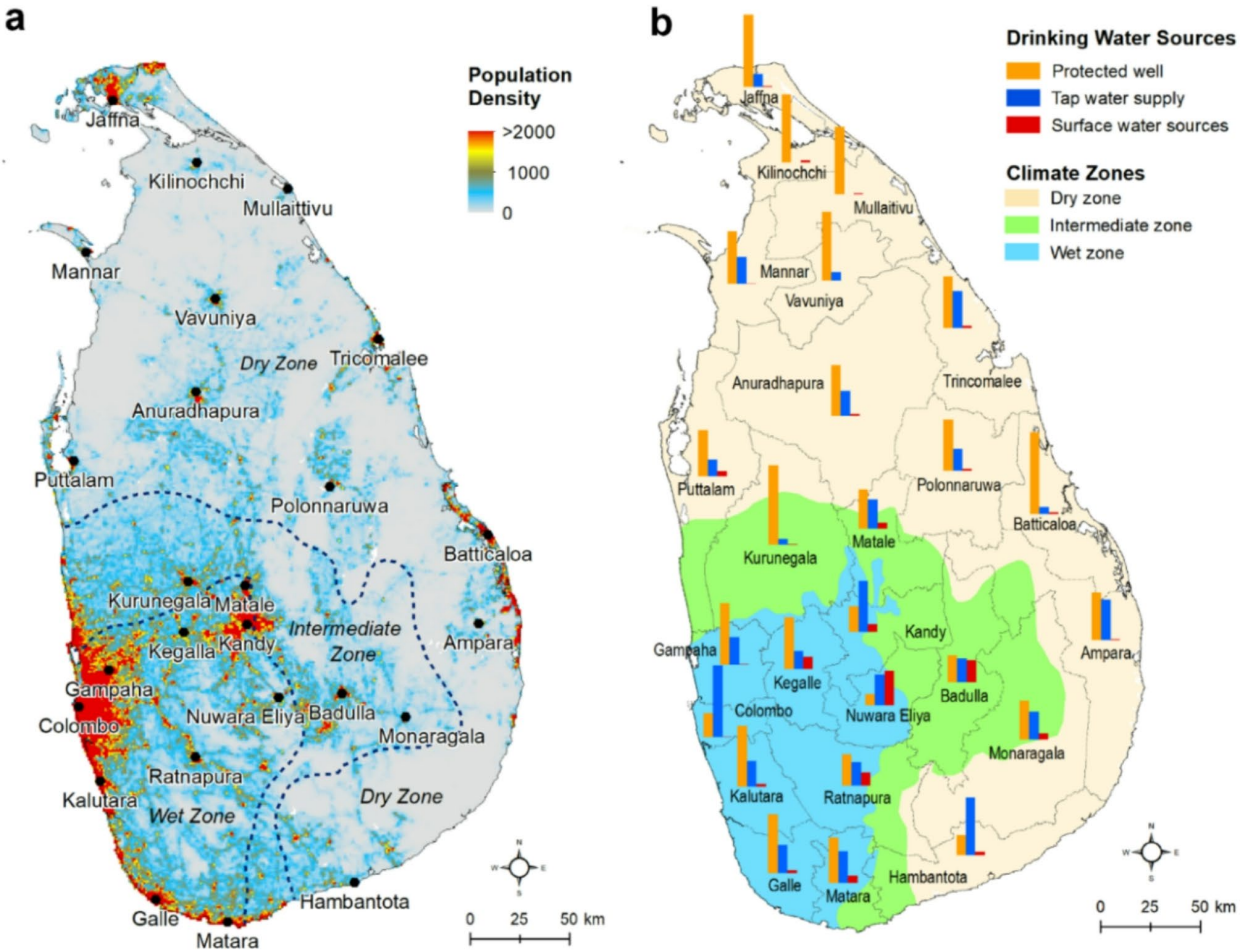
Location map of Sri Lanka showing population and drinking water sources. **a** distribution of gridded population density map (i.e., persons per square kilometer) across Sri Lanka for the year 2020 (data source: WorldPop, https://www.worldpop.org/). **b** map showing district-wise distribution of drinking water sources according to Sri Lanka’s 2012 Census of Population and Housing. The number of various drinking water sources (i.e., protected well, tap water and surface water supplies) is normalized by the number of households in each district (*n* = 25) in the country to show relative proportion of these water provisions. Note that tap water in Sri Lanka can be sourced from either treated surface water or groundwater. Both maps are produced in ArcGIS Desktop (v.10.8) software.

Located in South Asia, Sri Lanka is home to nearly 22 million people (Fig. 1a) in an area of 65,610 km^2^^6^. Surface elevation ranges from 0 to 2,524 m above mean sea level (msl), with elevated mountains located in the south-central part of the country (see supplementary Fig. S1). A detailed description of Sri Lanka’s hydrology, climate and geological conditions is provided in the supplementary information (supplementary text S1 and S2). Based on the spatial distribution of mean annual rainfall (Fig. S1b and S2), Sri Lanka is divided into three major agro-climatic zones^7^: the wet zone (rainfall > 2,500 mm), the dry zone (rainfall < 1,750 mm), and the intermediate zone (rainfall between 1,750 and 2,500 mm). Per capita total internally renewable water resource in Sri Lanka is approximately 2,500 m^3^/year^8^ – more than twice the per-capita freshwater resources in South Asia (1,100 m^3^/year)^8^.

In Sri Lanka, both surface water and groundwater resources are widely used for domestic, commercial, and industrial purposes, and small-scale irrigation. Over the years, Sri Lanka has come a long way in the provision for safe drinking water supplies to its population. For example, the WHO/UNICEF Joint Monitoring Programme for Water Supply, Sanitation and Hygiene reports^9^that 76% of all water supplies in Sri Lanka in 2000 was of improved quality, of which, piped water-supply was 22%, non-piped was 54% and nearly 10% of all water supplies came from surface water sources. Interestingly, groundwater coverage (40%) has not increased substantially over the same period^10^ suggesting that the increased piped water primarily comes from surface water sources. According to Sri Lanka’s 2012 Census of Population and Housing, the coverage of household drinking water sources is 46% (total of 5.2 million households) by protected dug wells (i.e., groundwater), 30% by tap waters and 5% by surface water sources (Fig. 1b). It is, however, reported that since the 2012 Census, piped water supply has increased from 30 to 52% from 2005 to 2020 through 331 large and small water-supply schemes across all provinces in Sri Lanka^4,11^. It should be noted that tap water through piped network can originate from both groundwater and surface water sources, following treatment.

Approximately, one-third of the 300 urban and rural water-supply schemes across Sri Lanka is based on supplies from shallow and deep groundwaters^12^. Approximately, 80% of rural domestic water-supply needs are met by groundwater though dependence on groundwater supply has substantially increased in the commercial and industrial sectors^12^. Most rural people in Sri Lanka heavily depend on dug and hand-operated tubewells since groundwater is perceived to be the safest drinking water source which can be self-managed^13^. In many areas, groundwater provides industrial and commercial water supplies where surface water sources are not fully reliable. Most industries in Sri Lanka largely depend on deep wells where groundwater is safe and of sufficient good quality^14^. The demand for groundwater in Sri Lanka is steadily increasing across all sectors in both urban and rural water supplies, irrigated agriculture, industries, aquaculture, private businesses, and urban water schemes. Despite the increasing reliance on groundwater, no systematic and routine monitoring of groundwater quality and levels exist in the country.

Poor quality of drinking water remains a pervasive problem for the world^15^. Water quality in surface water and groundwater varies regionally and locally across Sri Lanka^16^. For example, Mahagamage and Manage^17^reported poor water quality in the Kelani River basin that supplies 80% drinking water to the capital city, Colombo hosting more than a quarter of the nation’s entire population. Surface water-quality parameters (e.g., nutrients) were also found to be above the permissible limits recommended by the World Health Organization (WHO), the US Environmental Protection Agency (EPA), and Sri Lanka’s standards for drinking water. Watersheds are being degraded due to chemical and bacteriological pollution from domestic, agricultural runoff and industrial effluents. Concerns of contamination by nitrate and bacteria in surface water and groundwater due to poor sanitation and untreated or insufficiently treated wastewater, effluents from industrial and agricultural activities, and eutrophication in lakes or reservoirs are well documented^18,19^.

Groundwater chemistry in Sri Lanka has significant influences on the health of its population^20^. Many people living in the dry to intermediate climate zones use groundwater as the primary drinking water supply that is heavily influenced by the presence of geogenic contaminants such as heavy metals and fluoride resulting in certain diseases, e.g., dental and skeletal fluorosis^10^, and chronic kidney diseases^20^though it still remains unresolved. Groundwater in several areas in the dry zone is well-known to have elevated levels of fluoride that is thought to be linked to the occurrence of chronic kidney diseases due to an exposure of rural populations through drinking water intake. High blood pressure and pre-eclampsia during pregnancy^21^ are common in areas with the high salinity in drinking water.

However, because of the lack of continuous monitoring data (i.e., long-term, time-series records), it is difficult to assess the spatiotemporal trends in water-quality parameters in public water bodies which include surface water and groundwater sources. Studies conducted over the past years, mostly at local levels with a few national-level analyses, reveal the wider geographic extent and characteristics of contamination of water bodies across the country.

The need for national-level water quality maps has become a national priority^22^. This study, for the first time, conducts a national-scale analysis of ambient water quality in Sri Lanka that reveals geographic patterns in surface water and groundwater quality. We use single-point, one-off measurements of groundwater quality and one set of time-series data (2003 − 2016) for the surface water quality from the Kelani River. This goal is achieved through a geospatial mapping of groundwater quality data collated from the National Water Supply and Drainage Board and the Water Resources Board of Sri Lanka and surface water-quality data from the Central Environmental Authority. Multi-parameter groundwater-quality hazard index in created using available datasets and population exposure to poor groundwater quality is estimated at the district level. Finally, this paper discusses the current challenges to achieving the UN Sustainable Development Goals (SDG 6) and potential pathways of climate change impacts on water quality and future public water supplies in Sri Lanka.

## Datasets and methods

### Surface water quality datasets

Surface water-quality data used in this study derived from two sources: (1) gridded (0.5° spatial resolution) time-series (January 1992 to December 2010) of surface water quality data for five parameters from a global-scale analysis conducted by the World Bank^2,3^, data can be downloaded from https://qualityunknown.wbwaterdata.org/quality; and (2) time-series (January 2003 to June 2016) monitoring data on surface water quality for Kelani River in Sri Lanka. Source of the Kelani River water quality data is the Central Environmental Authority in Sri Lanka (http://203.115.26.11:8881/environmentalreport/dl; registration required). Fourteen physical and chemical parameters have been measured fortnightly to monthly intervals at 14 locations by the Central Environmental Authority in Sri Lanka since 2003. These parameters include pH, Electrical Conductivity (EC), turbidity, temperature, dissolved oxygen (DO), COD, Biological or Biochemical Oxygen Demand (BOD), Chloride, chromium (Cr), lead (Pb), nitrogen (NO_3_), phosphorous (PO_4_), Total coliform (T. coli) and Fecal coliform (F. coli) bacteria.

The World Bank report^2^ explored global water quality and its evolution for a 19-year period (1992 to 2010). Water-quality hotspots focused on five major parameters that were tracked by the Sustainable Development Goal (SDG) Indicator 6.3.2 (Proportion of bodies of water with good ambient water quality) and considered the most critical for surface water quality. These parameters include nutrients such as Nitrate-Nitrite (N) and Total Phosphorous (TP); measures of salinity such as Electrical Conductivity (EC); and widely used umbrella proxies for water quality such as Biochemical or Biological Oxygen Demand (BOD), and Dissolved Oxygen (DO).

### Groundwater quality datasets

Groundwater quality datasets used in this analysis come from the monitoring database managed by the National Water Supply and Drainage Board (NWSDB) at the national scale, and another groundwater quality dataset consists of 564 wells comes from the Water Resources Board (WRB) of Sri Lanka. The WRB data points cover seven districts where the agricultural drought study was conducted recently^23^. Neither of these datasets from the two national agencies in Sri Lanka reflects any temporal changes in groundwater quality, as the measurements are one-off, single-point samples taken during different time periods.

We collated 770 data points on various groundwater physical and chemical parameters from the NWSDB. However, 689 data points have location coordinates and one with no significant information. So, in our final analysis we used 688 georeferenced data points (see Supplementary text S3 and Fig. S3 for the location) that geographically cover the entire Sri Lanka. These wells are mostly water-supply wells that were installed by the NWSDB over a long period of time. The construction years of these 688 wells range from 1979 to 2020 with an annual mean of 16 wells/year and most of these wells were installed in the mid-1980s. The distribution of the 657 out of 688 wells, where the year of installation information is available in the dataset, is as follows: 1970s (0.6%), 1980s (35%), 1990s (25%), 2000s (12%), 2010s (28%) and 2020s (0.2%).

Water quality parameters, both physical and chemical properties, were measured during the time of installation of these water-supply wells that ranges from 1979 to 2020 with majority (60%) from the 1980s and 1990s (see Fig. S3 for well installation time with the geographic location). Therefore, these wells are not regularly monitored and are not suitable for time-series analysis. The physical parameters are well depth, color, turbidity, water levels, and yield of wells. Chemical parameters include pH, Chloride (Cl), Total Alkalinity (TA), Nitrate (NO_3_), Nitrite (NO_2_), Fluoride (F), Phosphate (PO_4_), Total Dissolved Solids (TDS), Total Hardness (TH), Iron (Fe), and Sulphate (SO_4_). The depth of these wells ranges from 4 to 105 m below ground level (bgl) with a mean of 44 m bgl and a median of 40 m bgl. The number of wells within various depths varies from only 5 wells below 10 m bgl, 25 wells below 20 m bgl to 328 wells below 40 m bgl. The depth of 197 wells is greater than 50 m bgl.

### Groundwater quality index and hazard mapping

The Water Quality Index (WQI) is a widely used tool for assessing surface water quality by converting complex water quality data into a single index^24,25^. Developed in the 1960s, the WQI has gained global popularity for evaluating both surface and groundwater based on local criteria due to its simplicity and versatility. The process typically involves four steps: selecting water quality parameters, generating sub-indices for each, assigning weighting values, and aggregating the sub-indices to calculate the overall WQI^24^. This method was applied to surface water and groundwater quality in various places around world including Sri Lanka^26^. In this study, we apply the Weighted Arithmetic Water Quality Index (WAWQI) method to calculate groundwater quality index for Sri Lanka. This involves ten parameters such as concentrations of Chloride (Cl), Alkalinity, Nitrate (NO_3_), Nitrite (NO_2_), Fluoride (F), Phosphate (PO_4_), TDS, Hardness, Iron (Fe), and Sulphate (SO_4_). Many of these parameters are correlated and tend to cluster together into distinct groups (see Fig. S4). Details about the WAWQI method can be found in recent literature^25,27^ and summarized in the supplementary information (supplementary text S4).

The Water Quality Index (WQI) method provides a general classification of water quality but does not explicitly quantify the extent or degree of hazards posed to health and ecosystem by specific chemical parameters. To address this limitation, we developed a groundwater-quality hazard index based on chemical parameters that do not meet drinking water standards. This approach allows for a more targeted identification of potential health risks by highlighting specific contaminants that either exceed safe limits or are so low that they do not provide health benefits, offering a clearer representation of the groundwater hazard. Groundwater multi-parameter hazard index maps are useful tools for visualization of composite hazards and their geospatial variability^28^. Groundwater multi-parameter hazard index mapping is conducted using well data collated from the National Water Supply and Drainage Board (NWSDB) as this dataset covers the entire country. A variable number of groundwater samples was selected for each district (Supplementary Information). In this study, a simple method is developed (see Eq. 1) to create a geospatial groundwater-quality hazard map using the NWSDB groundwater quality dataset that has 688 data points.

Two methods are used to calculate the district-wise hazard index: (1) using equally spaced interpolated grid values (i.e., interpolated method), and (2) using groundwater observation point values within each district (i.e., imputed method). Interpolated raster maps were generated using the available data points for each groundwater-quality parameter applying the Ordinary Kriging and the Inverse Distance Weighting (only for Nitrate to preserve local variations and prevent over-smoothing) algorithms in ArcGIS Desktop software (v. 10.8). Interpolation accuracy is assessed within the ArcGIS environment through the built-in root mean square standardized errors (RMSE) where a value close to 1 was considered to be a prediction model with a high degree of accuracy. Interpolated grid (approximately 2-km spatial resolution) values of ten water-quality parameters in each of the 25 districts in Sri Lanka were used to create water-quality hazard index using the following equation:

1 $$\:{GW}_{hazard\:index}=\frac{1}{n}{\sum\:}_{par}^{1}\left(No\:of\:{points\:or\:grids\:in\:GW}_{par}>{WHO}_{std}\right)$$

$\:{GW}_{hazard\:index}$ is groundwater multi-hazard index based on the proportion of grids with concentrations of water-quality parameters $\:{GW}_{par\:(n=10)}\:$exceeding the WHO and the US EPA standards (Table 1) at an interpolated grid point located within each of the 25 districts in Sri Lanka. Only exception is that for total iron concentrations the criterion is reversed, i.e., iron concentrations below the WHO recommended standard was used as literature suggests^29,30^that low iron concentration in drinking water is linked to anemia among children and women particularly during pregnancy^31^.

To compare the results from interpolated grids, we apply the same hazard index calculation method as described above but for point observations. However, the key problem is the inconsistency in the number of data points per groundwater-quality parameter. Analysis is performed in R programming language^32^ on 688 data points. Exploratory analyses reveal that not all parameters have 688 observations. There are missing data for several groundwater quality parameters (Table 1). Missing data gaps vary substantially from as little as 4% (hardness) to as high as 71% (phosphate).

For the estimation of $\:{GW}_{hazard\:index\:}$all missing data gaps are imputed or infilled using a nonparametric missing value imputation method using Random Forest machine learning algorithm from the missForest package in R software^33^. Random Forest algorithm does not require a validation dataset in this case, as it uses a technique called out-of-bag (OOB) to evaluate the quality of the imputation model in this case. OOB evaluation treats the training dataset as if it were on the test dataset of a cross-validation.

Performance of data imputation by missForest package is evaluated by the normalized root mean squared error (NRMSE), which is defined in Eq. 2. For this study, the overall imputation error is 22% which is considerable but can be accepted to be reasonable given the high range of missing values among the imputed variables^33^.

2 $$\:\sqrt{\frac{mean\:{\left({X}_{true}-{X}_{imp}\right)}^{2}}{var\left({X}_{true}\right)}}$$

Where $\:{X}_{true}$ is the complete data matrix, $\:{X}_{imp}$is the imputed data matrix and mean/var being used as short notation in Eq. 2 for the empirical mean and variance computed over the continuous missing values only^33^. We report the groundwater hazard indices resulted from both methods.

In addition, we explore the impact on groundwater quality hazard on population health, we collate district-wise data on cases of stunting among under 5 years old children and anemia cases in both children and women in 25 districts from the Demographic and Health Survey 2016 data for Sri Lanka^34^. To examine the relationship between iron and anemia at the district level, we apply Pearson correlation and simple linear regression models in R programming language.

### Focused review of surface water quality

We undertook a targeted literature review on surface water and groundwater quality in Sri Lanka. Two academic research databases (i.e., Web of Science and Scopus) were selected due to extensive coverage of literature and the capability of combining complex keyword strings. The search terms were “water quality in Sri Lanka” or “water pollution in Sri Lanka”; the literature search was conducted on 23 March 2022. In addition, we have looked through all literature from the World Bank report, “Sri Lanka Water Quality Study”, published in 2021.

As a result, 395 pieces of literature were identified for further screening. Abstracts were screened after removing duplicate entries based on their relevance to the research criteria. Firstly, articles were included for the next step if the full texts were available via databases and written in English. We manually excluded water-quality-related papers that passed the first-level selection criteria but focused on topics such as water technology for agriculture, water treatment efficiency (e.g., filtration systems), bottled water use, and microplastic contamination – none of which fall within the scope of our analysis, which focuses on ambient water-quality situation analysis.Through the screening process, a total of 116 (91 from the in-depth literature search and 25 from a World Bank report) papers were finally included in this review for data extraction and synthesis. A list of the literature surveyed in this review analysis is provided in supplementary Table S2.

A dominant number of papers (see supplementary Table S2) discussed groundwater contamination and its potential link to chronic kidney disease of unknown etiology (CKDu) in North Central Province (Polonnaruwa and Anuradhapura districts), which are known to be vulnerable areas. Regarding surface water, Kelani River and Kandy Lake are the most well studied and focused water resources, and the geographical coverage of surface water study was not well distributed. Many water quality parameters were monitored and presented as summary statistics, but raw data was unavailable for most cases. This creates a barrier to extracting data from this in-depth literature review.

### Limitations and sources of uncertainties

There are several limitations and sources of uncertainties in the analysis. One major limitation in our study is that the 688 groundwater-quality data points from sampled wells are distributed across different geographic areas and span a time range from 1979 to 2020. Most of these wells (approximately 60%) were installed by the National Water Supply and Drainage Board (NWSDB) during the 1980s and 1990s. For our national-scale water-quality hazard mapping, we included all wells with available information, regardless of temporal variations. We assume that groundwater quality has largely remained stable over time, though this may vary depending on natural and anthropogenic factors such geology, land use and climate change, groundwater abstraction, and recharge conditions. This assumption, though not perfect, is necessary due to the lack of consistent temporal data. Caution should be exercised when interpreting groundwater quality dynamics in Sri Lanka, as the absence of long-term monitoring data limits our ability to assess temporal variations, trends, and potential episodic contamination events.

The availability of time-series monitoring data on surface water quality across the entire Sri Lanka is also very limited. In this study, we only used monitoring data for the Kelani River basin. We used machine-learning algorithm generated gridded data on surface water parameters for Sri Lanka derived from a global-scale mapping study by the World Bank^2^, and the uncertainty in the gridded data can be high at the country scale. Other sources of uncertainties in our analysis derive from imputation of missing data using the random forest algorithm available through the missForest R library and geostatistical interpolation of groundwater parameters at the national scale using algorithms available with the ArcGIS environment. The number of data points for Phosphate in the groundwater dataset is low (*n* = 199). This contributes to some level of uncertainty in the national-scale interpolation. The Central Laboratory Services at NWSDB implements rigorous sampling and analytical protocols for all water-quality assessments and chemical analyses accredited by the Sri Lanka Accreditation Board for Conformity Assessment. Lack of national-scale health outcomes data to make direct associations between water quality and health exposure is also a limitation of our analysis.

**Table 1:** Groundwater quality parameters and their descriptive statistics at the National scale in Sri Lanka (data source: National water supply and drainage board, NWSDB).

Variable	Unit	WHO / US EPA Standards	Sri Lanka Standards^†^	No of data points	Mean	Median	Std. Dev.	Minimum	Maximum
Well depth	m	N/A	N/A	688	43.6	40.0	16.0	4.0	105
Yield	liter/min	N/A	N/A	669	136.7	35.0	334.0	0.3	3200
GWL depth	m	N/A	N/A	509	13.5	11.0	8.8	1.0	61
pH	N/A	6.5–8.5	6.5–9.0	675	7.3	7.4	0.7	4.2	9
Chloride	mg/liter	250	200–1200	598	191.3	50.0	487.5	1.0	4930
Alkalinity	mg/liter	400^‡^	400	613	208.8	181.0	142.4	8.0	960
Nitrate	mg/liter	10	10	316	2.6	0.7	5.6	0.0	48
Nitrite	mg/liter	1.0^*^	0.01	208	0.4	0.01	1.8	< 0.01	21
Fluoride	mg/liter	1.5	0.6‒1.5	613	0.9	0.6	0.9	0.01	8
Phosphate	mg/liter	0.1^§^	2.0	199	0.7	0.4	1.0	< 0.01	9
TDS	mg/liter	600^††^	500‒2000	348	584.7	324.5	873.5	15.00	8322
Hardness	mg/liter	500	250‒600	659	266.3	180.0	368.4	10.00	3880
Iron	mg/liter	0.3	1.0	620	2.7	0.6	7.4	0.01	120
Sulphate	mg/liter	400	200–400	437	42.3	19.0	85.4	1.00	776

^†^Standard values are taken from the Sri Lanka Standards for potable water^35^ where there highest desirable as well as maximum permissible limits for certain parameters.

^††^A value between 300 and 600 mg/liter is considered to be good^29^.

^‡^No WHO standard for Alkalinity is found; this standard is used by some US states^36^.

^*^Nitrite standard is taken from the United States Environmental Protection Agency (EPA)^37^.

^§^US EPA recommends a standard of 0.1 milligrams per liter (mg/L) for total phosphorus^38^.

## Results

### Status of surface water quality and contamination

Sri Lanka has 103 rivers of which around 20 are perennial originating in central and southern highlands where rainfall is the highest in the country^5^. Catchment areas range from 10 to 10,000 km^2^and, together, they cover about 90% of the total land area^39^ (Fig. S5). Approximately 6% water supply in Sri Lanka comes from surface water. Piped water supply derived, in part, from surface water including major rivers in the country and water quality varies greatly.

**Fig. 2:**
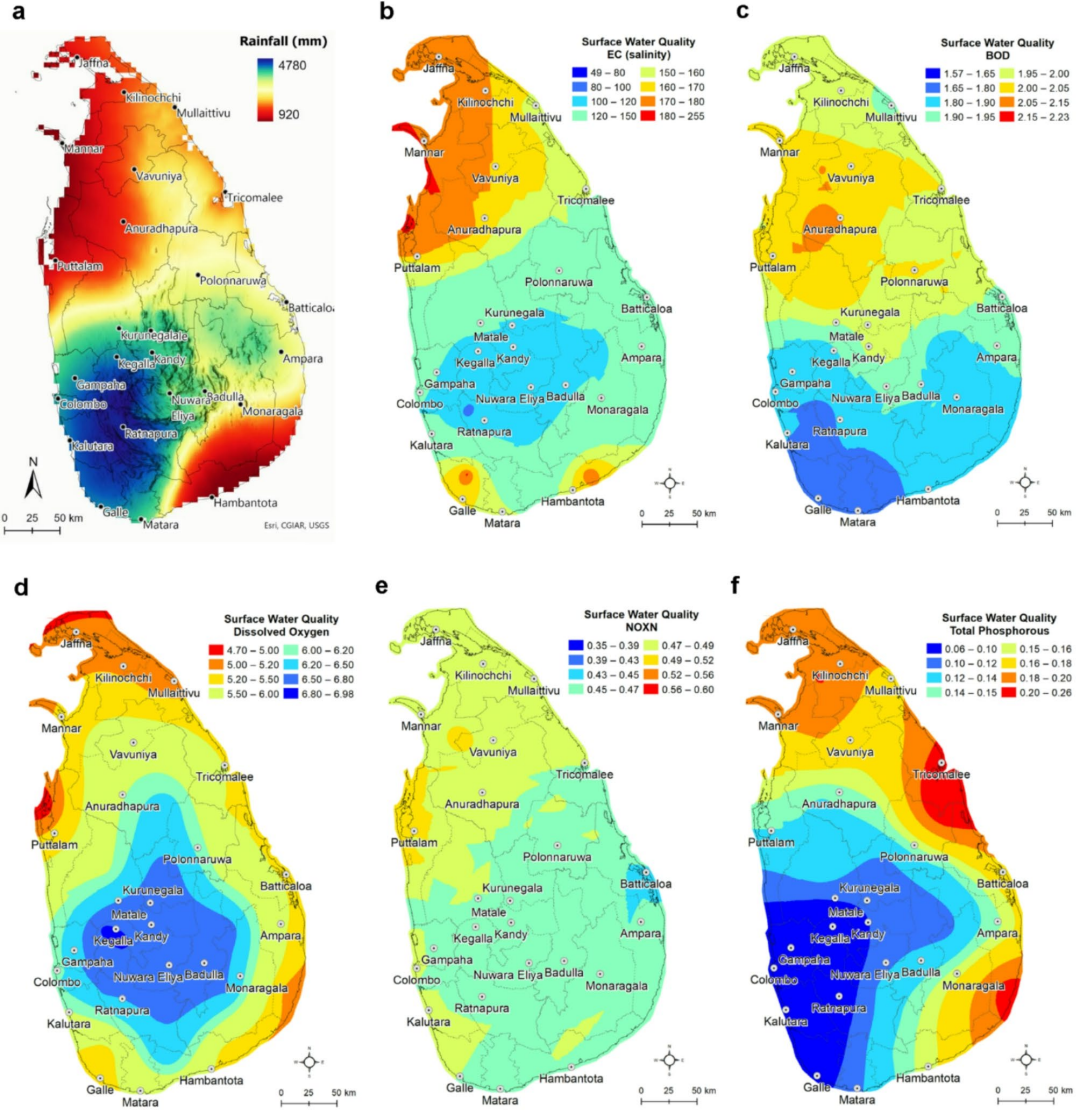
Geostatistically interpolated maps of mean annual rainfall and mean (1992–2010) surface water-quality parameters across Sri Lanka. **a** mean annual rainfall; **b** Electrical Conductivity or EC (salinity); **c** BOD (Biochemical Oxygen Demand); **d** Dissolved Oxygen (DO); **e**; NOxN or Nitrates (NO_3_) and Nitrites (NO_2_); and **f**Total Phosphorous (TP). Further details about these gridded data can be found in Damania, Desbureaux^2^. The unit of all parameter values is mg/L except for EC which is µS/cm. The Empirical Bayesian Kriging interpolation method is used for creating these maps from 22 equally spaced grid points in ArcGIS Desktop (v. 10.8).

**Fig. 3:**
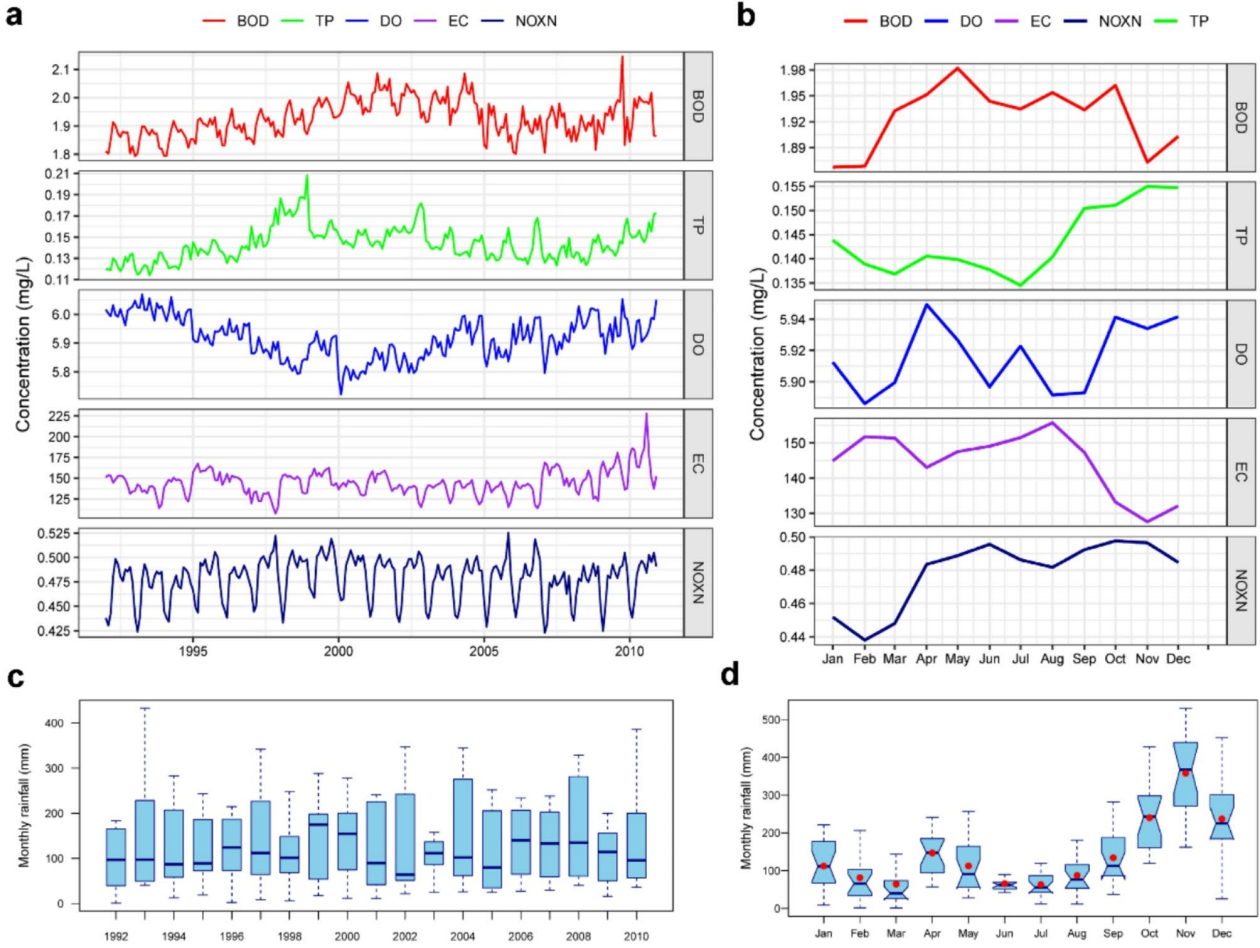
Aggregated long-term (1992–2010) plots of surface water parameters across Sri Lanka. **a** monthly time series; and **b** seasonal variability in BOD (Biochemical Oxygen Demand), Total Phosphorous (TP), Dissolved Oxygen (DO), Electrical Conductivity or EC (µS/cm) and NOxN or Nitrates (NO_3_) and Nitrites (NO_2_). Gridded data source: World Bank^2,3^ **c** inter-annual variability in monthly rainfall in each year from 1992 to 2010 shown in Box and Whisker plots; and **d**averaged monthly climatology of rainfall in Sri Lanka. Rainfall time-series (1992 to 2010) data derive from global gridded dataset provided by the Climatic Research Unit (CRU), University of East Anglia (CRU v. TS4.05). Graphs are created in R programming language^32^.

**Fig. 4:**
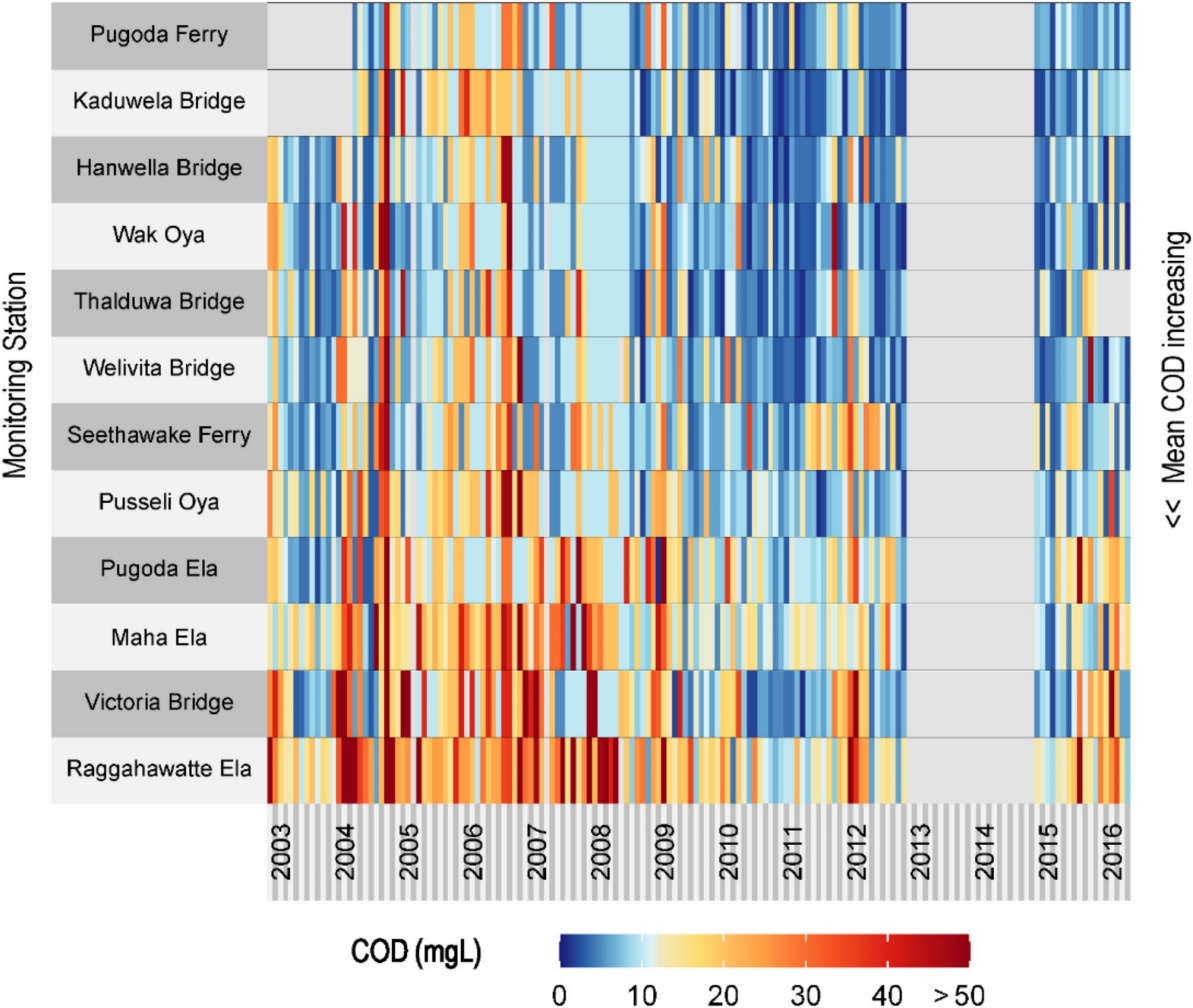
Long-term (2003–2016) changes in Chemical Oxygen Demand (COD) in 12 monitoring sites along Kelani River in Sri Lanka. Stations are arranged from top to bottom of the heatmap according to their mean COD values. Gaps in the monthly time-series data (2013–2014) are shown in light grey shade. Data source: The Central Environmental Authority (http://203.115.26.11:8881/environmentalreport/dl; registration required to access the data). Location of the stations and heatmaps of other water-quality parameters are provided in the supplementary information (see Figs. S9-S17). The heatmap is produced in R programming language^32^.

**Fig. 5:**
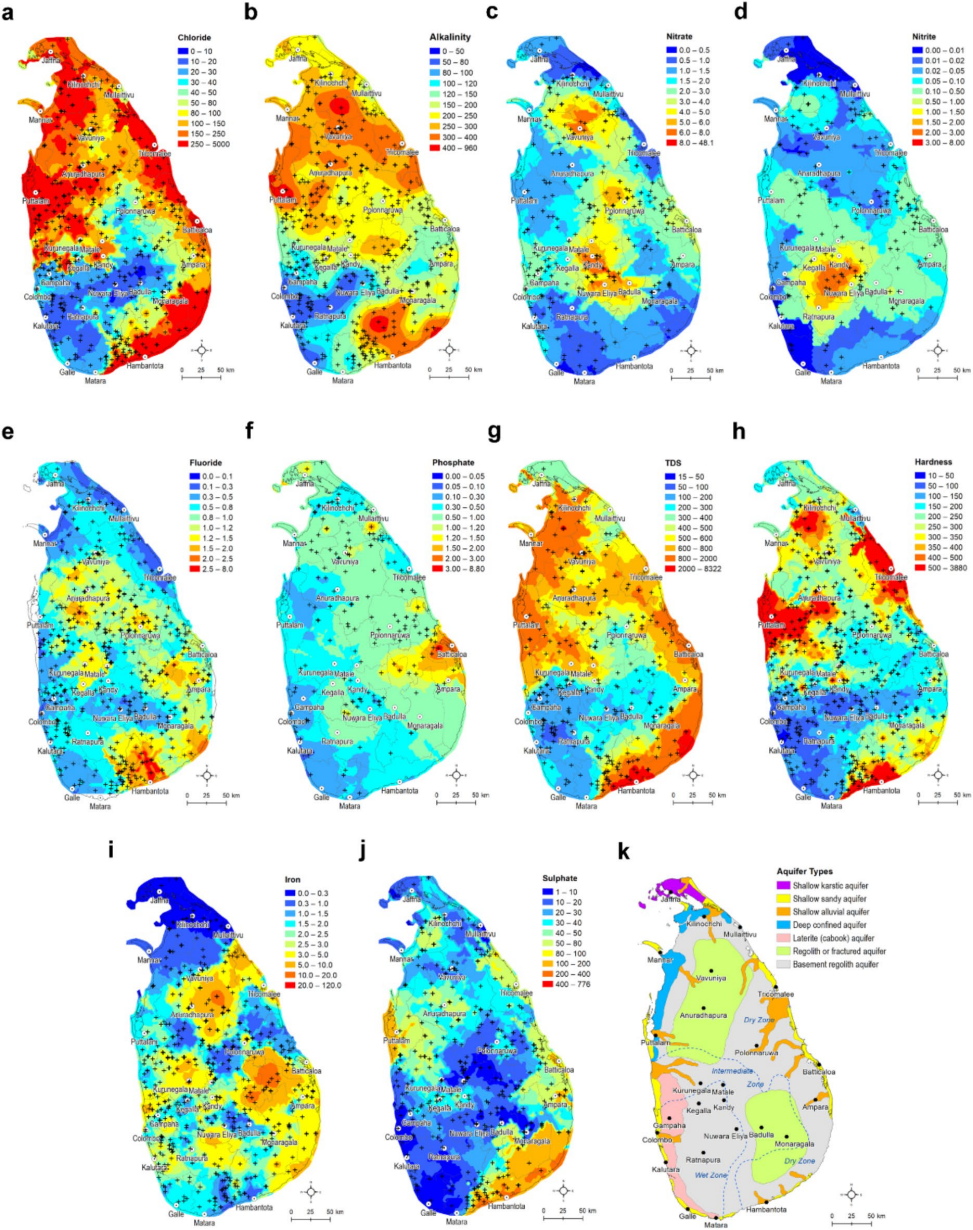
Spatially interpolated gridded maps of groundwater quality and hydraulic parameters across Sri Lanka. **a** chloride; **b** alkalinity; **c** nitrate; **d** nitrite; **e** fluoride; **f** phosphate; **g** total dissolved solids (TDS); **h** hardness; **i** total iron; **j** sulphate and **k** aquifer types and distribution in Sri Lanka. Data source: values are interpolated from variable data points (maximum number is 688) water-point locations available within the National Water Supply and Drainage Board (NWSDB) database. The location of data points for each parameter is also shown on the maps as black cross. Ordinary Kriging and Inverse Distance Weighting (only for nitrate) interpolation methods are used for creating these maps in ArcGIS Desktop (v. 10.8) from available point observations for each parameter (points also shown on these maps).

Surface water-quality data from a global-scale analysis conducted by the World Bank^2,3^ show variability in spatial (Fig. 2) as well as temporal (Fig. 3) scales at the national level in Sri Lanka. For example, surface water salinity represented by Electrical Conductivity (EC) values is higher (> 180 µS/cm) in the dry climate and lower (< 100 µS/cm) in the wet climate zones. These EC values are much lower than the suitable salinity levels (~ 750 µS/cm) for drinking water in order to maintain good human health^28^. Freshwater EC can be up to 1,500 µS/cm. Mean Biological Oxygen Demand (BOD) values are ~ 2 mg/L in the dry climate zone, suggesting moderately polluted rivers^2^. Dissolved Oxygen levels across the country is generally above 5 mg/L, within Sri Lanka’s bathing and contact recreational water standard (National Environmental Regulations, 2019) except a few coastal areas in the north, northwest and southeast within the intermediate and dry climate zones (Fig. 4). Total Phosphorus (TP) and Nitrogen Oxides (NOxN: NO_2_ and NO_3_) values are also within the Sri Lankan ambient water-quality standards. Long-term trends (1992 to 2010) in surface water-quality parameters (Fig. 5) show temporal variability with discernible seasonality and slightly increasing trends in all five parameters (BOD, TP, DO, EC and NOxN) since 2000. EC has an inverse relationship with seasonal rainfall, whereas TP and NOxN show a positive association with rainfall suggesting flushing of agrochemicals with rainwater. However, the trends of these surface water quality parameters in Sri Lanka’s dry and intermediate to wet climate zones show slightly different long-term patterns of change (see Figs. S6 and S7).

The most important rivers in terms of area, length and human use for drinking water and irrigation water supplies are the Mahaweli Ganga, Malwathu Oya (Aruvi Aru), Kala Oya, Deduru Oya, Kalu Ganga, Walawe Ganga, Kelani Ganga, Yan Oya, Gal Oya and Mi Oya (Fig. 4). Surface water from many of these rivers is being used for drinking water supplies^40^. The Mahaweli River is the longest river of Sri Lanka with a length of 335 km crossing three different provinces: Central, North Central and Eastern (Fig. S5). Other rivers that provide public drinking water supplies are Deduru River which runs through the Northwestern province and feeds into the Deduru reservoir.

The Kelani River with the second largest watershed (2,298 km^2^) in Sri Lanka has been playing an important role in the country’s water supply and economy for several decades^41^as it drains the most fertile land in the wet climate zone and through most populated (2.1 million people live in the river basin) (Fig. S5b; Table S3) and economically important administrative district (Western Province) including Colombo – the Capital City^42^. Kelani River is the main water source for 80% Colombo district’s total population^43^. Mahagamage and Manage^17^ reported that most water abstraction sites within the Kelani River have poor water-quality index. In this study, we collated time-series data of surface water quality of the Kelani River on fourteen physio-chemical parameters from 2003 to 2016 (Table S4).

Long-term (2003–2016) monitoring of water-quality of the Kelani River shows changes in the water-quality parameters over time at 12 locations (see Fig. S8 for location). Figure 4 shows temporal changes in monthly Chemical Oxygen Demand (COD) measurements in Kelan River at 12 locations along the river. Heatmaps of other water-quality parameters are presented in Figs. S9-S17. The COD standard for Category A (i.e., water that requires simple treatment for drinking) and Category B (i.e., water for bathing and contact recreational purposes) is 10 mg/L. It appears from the heatmap that COD levels have decreased in many stations since 2010; however, in some locations (e.g., Raggahawatte Ela, Victoria Bridge, Maha Ela) the COD levels are excessively high compared to Sri Lanka’s standard of 10 mg/L^35^. The trend analysis of COD time-series data (2003–2016) suggests a declining linear trend over time (ranging from − 0.40 mg/L/year at Thalduwa Bridge to −6.4 mg/L/year at Eswathu Oya) at all locations along the Kelani River, except for Pugoda Ela, which shows a slight rising trend of 0.1 mg/L/year. In addition, microbiological (bacterial) contamination of water in Kelani River is a major concern for surface water management. The entire Kelani River Basin was found to be contaminated with total coliform bacteria and *Escherichia coli*(E. coli) bacteria that best indicate fecal pollution and the possible presence of pathogens^44^.

Other notable water-quality issues linked to Sri Lanka’s surface water bodies include nutrients pollution affecting Uma Oya (upper catchment of Mahaweli Ganga), the Mahaweli Ganga and the Gin River^45^. The algal blooms are reported in several inland freshwater reservoirs. The harmful cyanobacterial blooms found in 17 freshwater reservoirs in Sri Lanka^46^. Heavy metal pollution with mercury and lead was reported in several river basins including Mahaweli, Deduru and Gin rivers^40^.

### Status of groundwater quality and contamination

Groundwater quality in Sri Lanka is highly variable (Fig. 5) within the country but appears to be controlled by climate and geology and the distribution of aquifer systems (see aquifers in Sri Lanka in Fig. 5k and supplementary Fis. S18). In this study, we analyze 688 single-point groundwater quality data from the National Water Supply and Drainage Board (NWSDB) and 564 from the Water Resources Board (WRB) databases (Fig. S3) using R programming language^32^. For mapping, we interpolate groundwater parameters at the national scale using geostatistical interpolation algorithms in ArcGIS Desktop software (v.10.8).

The NWSDB samples cover all 25 districts, but the number varies (Table S5). We also calculate summary statistics of each groundwater-quality parameter within the aquifer units, climate zones and districts (see supplementary Tables S6-8). Table 2 shows the mean values of some selected groundwater quality variables within three climate zones in Sri Lanka. Clear variations of some variables (e.g., fluoride, hardness, salinity indicated by TDS and chloride concentrations) are seen in three climate zones.

**Table 2:** Mean groundwater quality parameters within each climate zone in Sri Lanka. The unit for well depth and groundwater-level depths is meter (m), and for all water-quality parameters the unit is mg/l.

Climate Zone	Well Depth	Yield (L/min)	GWL Depth	pH	Chloride	Alkalinity	Nitrate
Dry zone	41.6	125.8	11.6	7.4	279.2	262.9	2.5
Intermediate zone	42.6	91.6	14.6	7.2	129.0	193.2	2.9
Wet zone	49.9	223.1	17.0	7.2	60.20	104.9	2.8

	Nitrite	Fluoride	Phosphate	TDS	Hardness	Iron	Sulphate
Dry zone	0.1	1.0	0.7	875.3	343.2	2.3	54.4
Intermediate zone	0.5	0.7	0.6	436.2	234.4	3.3	32.0
Wet zone	1.3	0.5	0.4	178.7	127.3	2.6	18.4

#### Groundwater quality index and multi-parameter hazard map

**Fig. 6:**
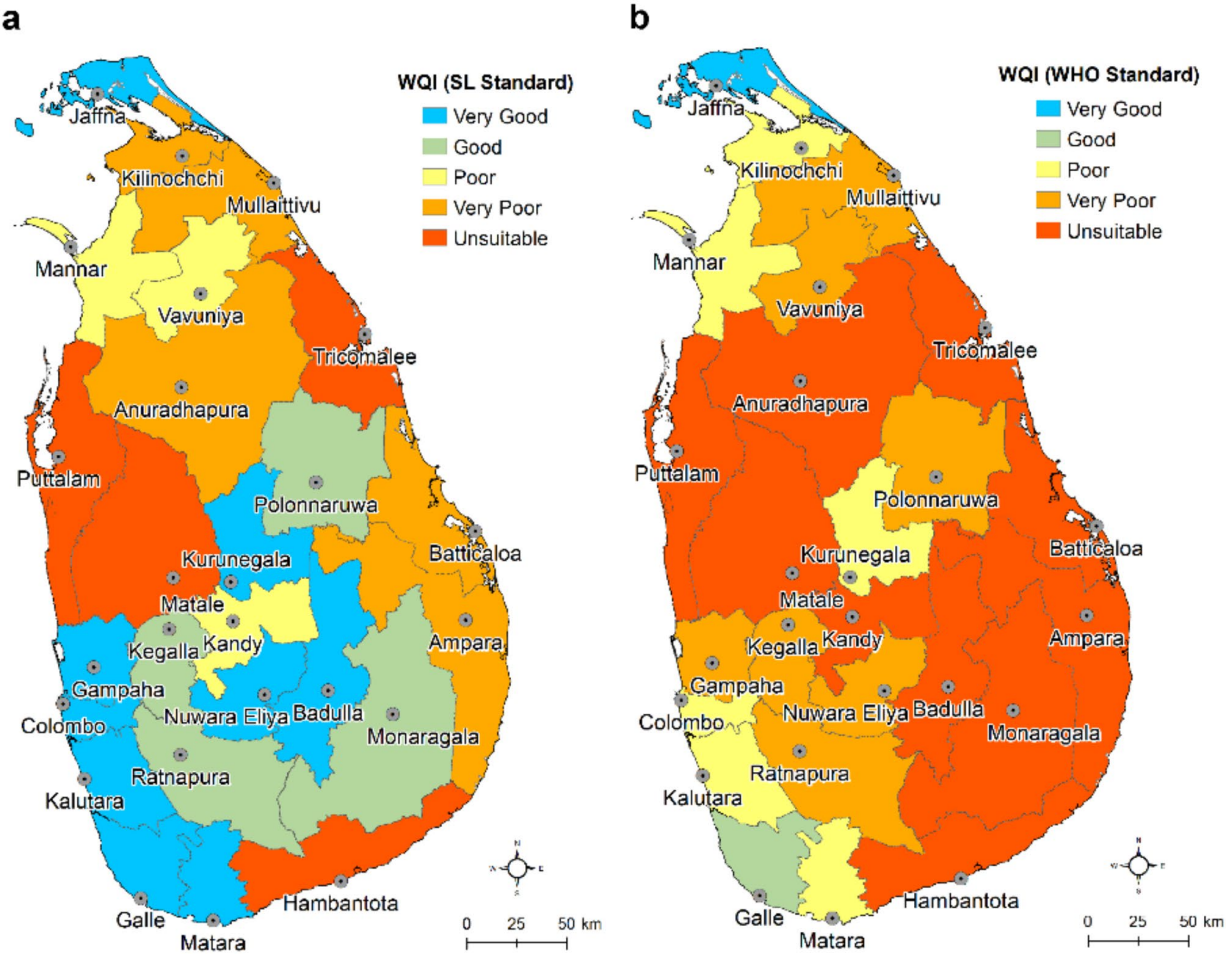
Groundwater quality index (WQI) maps of Sri Lanka. **a** District-wise WQI scores and classification of groundwater based on the Sri Lanka drinking water standards. **b** WQI scores and classification of groundwater based on the WHO and US EPA standards. These original GIS maps are produced in ArcGIS Desktop (v.10.8) software.

Groundwater quality index (WQI) and multi-parameter hazard index mapping are conducted using a total of 688 groundwater quality data points collated from the NWSDB database applying a method developed in this study considering the WHO and other international standards such as the US EPA. Aggregated WQI scores in the 25 districts of Sri Lanka based on the Sri Lanka standards and the WHO and US EPA standards show different groundwater-quality status (Fig. 6) due to certain parameters (e.g. Phosphate). Results show that only a few districts in Sri Lanka present very good to good quality groundwater sources.

**Fig. 7:**
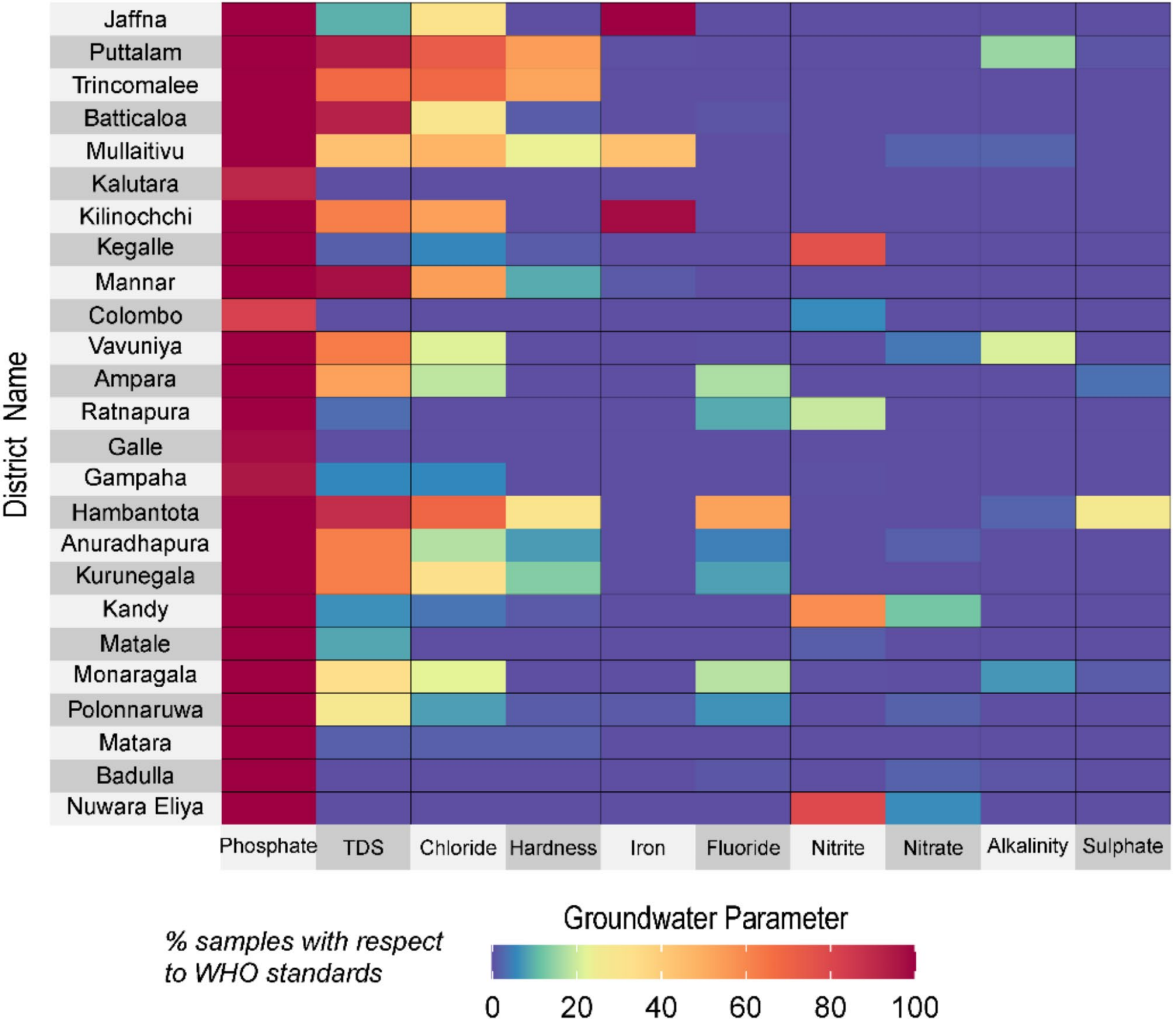
Heatmaps showing groundwater quality in 25 districts in Sri Lanka. Proportion (in percent) of interpolated grids of groundwater physicochemical parameters exceeding (below for iron concentrations in groundwater) the corresponding WHO and the US EPA standards in each district across Sri Lanka.

Groundwater risks (i.e., risk = hazard x exposure x vulnerability) to human health starts with the hazard where a single chemical constituent or a combination of several constituents (multiple chemical parameters) can characterize the hazard. We have not produced a groundwater quality risk map in this study due to the lack of information on actual groundwater users (exposure) and vulnerability (e.g., access to wells). Here, we have analyzed ten chemical parameters such as Chloride (Cl), Alkalinity, Nitrate (NO_3_), Nitrite (NO_2_), Fluoride (F), Phosphate (PO_4_), TDS, Hardness, Total Iron (Fe), and Sulphate (SO_4_) in 25 districts in Sri Lanka and their proportion (percentage) not meeting the WHO and US EPA standards (Table 1) that varies across the country (Fig. 7). In the case of iron concentration, we consider the reverse criterion where low concentrations of iron (less than the WHO recommended standard of 0.3 mg/L), though not considered to be a health hazard, are less beneficial to health, particularly for children and women^47^. The importance of iron in drinking water is recognized to protect impoverished young children from anemia^48,49^. Low intake of iron through drinking water can lead to anemia among children and women when their dietary intake of iron is also low^29^.

**Fig. 8:**
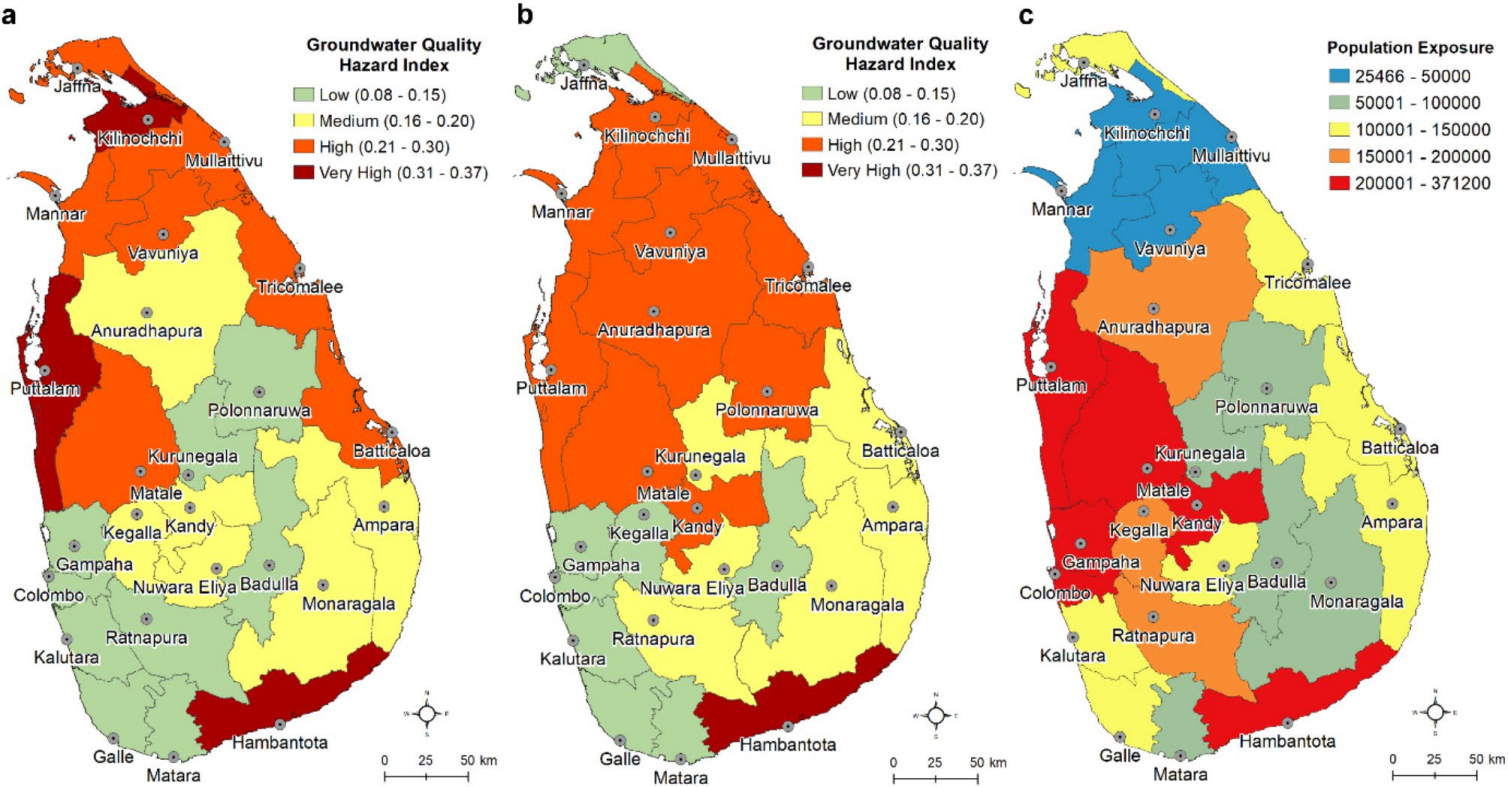
Groundwater multi-parameter hazard index and population exposure maps of Sri Lanka. **a** District-wise groundwater-quality hazard index based on ten groundwater-quality parameters based on interpolated raster grids (interpolation method); **b** District-wise groundwater-quality hazard index based on ten groundwater-quality parameters based on imputed point observations (imputation method), and **c** district-wise estimated population exposure based on total population of the district regardless of their sources of drinking water to the groundwater-quality hazard in Sri Lanka. Note that the exposure of population in even some low-risk (primarily due to low level of hazard) districts is high (e.g., Colombo, Gampaha) due to higher population number and greater density. These original GIS maps are produced in ArcGIS Desktop (v.10.8) software.

Groundwater multi-parameter hazard index map (Fig. 8a) is created using the sum of all hazard index scores at all gird points within each district. Population exposure to various levels of groundwater hazard levels is calculated using the multi-parameters scores and the corresponding number of people (Fig. 8b) living in each of the 25 districts. Based on cumulative multi-parameter hazard scores, our analysis shows that groundwater users in Hambantota, Puttalam, Batticaloa, Kilinochchi, Jaffna, Mullaitivu, Kurunegala, Mannar, Trincomalee and Vavuniya districts in Sri Lanka are presumably at most health risk due to high hazard resulting from poor-quality groundwater. Districts with low groundwater quality multi-parameter hazard index are Colombo, Galle, Gampaha, Kalutara, Badulla, Matale and Matara.

## Discussion

Water quality is intrinsically linked to human health and nutrition. Nationally, 85% of Sri Lanka’s water supplies came from improved sources, as of 2016, of which 36% is piped water supply, 50% is non-piped water supply and ~ 6% water still comes from surface water. Piped water supply derives from both surface and groundwater sources though no data on water quality are available. Interestingly, groundwater provides only 40% of water supplies nationally, though more than 70% of rural populations depend on it. Large cities like Colombo and Kandy are primarily dependent on surface water for their drinking water supplies^26^.

Poor drinking water quality regardless its sources has been impacting human health in Sri Lanka. Commonly occurring health issues that are linked to poor quality water are dental and skeletal fluorosis^50^, chronic kidney disease with uncertain etiology (CKDu)^51,52^, and water-borne diseases^44^. Recently, the development of antimicrobial resistance to antibiotic drugs has been linked to emerging antibiotic contamination in water^53,54^. The residues of widely used antibiotics such as penicillin, tetracyclines, and sulfamethoxazole were detected in Kelani River^54^. Liyanage and Manage^53^ reported that surface and groundwater in the Kelani River catchment are a possible reservoir of two antibiotics, namely, penicillin and tetracycline and their resistance genes. Furthermore, our review analysis reveals that microplastic is another emerging pollutant found in the coastal areas in Sri Lanka, particularly along the southern coastal belt including the marine protection area^55^.

Our population exposure analysis of groundwater quality multi-hazard index at the district level reveals that approximately 3.6 million (interpolation method) to 3.8 million (imputation method) out of approximately 22 million people (16.4–17.4%) in Sri Lanka are exposed to poor groundwater quality through drinking (Fig. 8c). The district with the highest hazard index (0.37) is Hambantota and lowest hazard index (~ 0.09) is Colombo. More than 1.5 million people in Kandy, Kurunegala, Puttalam, Hambantota, Gampaha and Colombo districts alone are exposed to some degree of poor groundwater quality based on the hazard index analysis. Shallow sandy and alluvial aquifers, and fractured Regolith aquifer (see Fig. S18) in the north are particularly low in ambient groundwater quality in Sri Lanka.

Rural populations are particularly vulnerable to adverse health outcomes as they increasingly depend on groundwater. Several adverse health outcomes are reported to be linked to drinking water intake that comes from poor-quality groundwater. For example, intake of high saline water through drinking can cause hypertension and pre-eclampsia during pregnancy as reported in Bangladesh^21^. High fluoride concentrations in groundwater lead to dental and skeletal fluorosis^56^. Other health related outcomes linked to chemical constituents in drinking water include methemoglobinemia in children and stomach cancer in adults for exposure to very high concentrations of nitrate and nitrite^57^. Water alkalinity which is linked to pH can affect health indirectly due to contamination of drinking-water through excessive pipe corrosion^29^, and alteration of taste and appearance that may lead to insufficient fluid intake.

Our multi-parameter hazard index does not include all the toxic and high-priority elements found in groundwater such as arsenic (metalloid) and other heavy metals (e.g., lead, nickel) due to lack of point observations in the national-agency (NWSDB) one-off monitoring dataset. However, our in-depth review of literature reveals that an academic research was conducted in Sri Lanka that collecting 1,304 data points and analyzed for arsenic^50^. Water samples were collected from the wells in all 25 districts in Sri Lanka from 2010 to 2014. District-level maps of groundwater arsenic show that arsenic is generally low in Sri Lanka (see Fig. S19). Only in Mannar, Puttalam and Batticaloa, Mullaitivu and some parts of Anuradhapura districts that are located in the dry zone groundwater has high (> 10 µg/L) concentrations of arsenic^10^. Groundwater in Colombo districts has high concentrations of chemical oxygen demand (COD) and biochemical oxygen demand (BOD) indicating anthropogenic contamination of water^58^. Higher concentrations of electrical conductivity, total dissolved solids, hardness, chloride, and fluoride in groundwater in intermediate and dry climate zones originate from water-rock interactions and high evaporation rates^59^.

Heavy metal (Cd, Pb) concentrations are found to be elevated in shallow groundwater in the northern dry zones such as Anuradhapura district^60^where cases of CKDu cases are the highest in the country^61^, but this is conclusive and thus debatable. The natural variability in water nutrients (Na, Ca, Mg) in groundwater along with high fluoride levels is an important factor for increased occurrence of kidney diseases^62^. Cases of iron deficiency among children in Sri Lanka is well known^63^. Associations between geology, aquifer types, and groundwater quality need to be studied in detailed to characterize the origin and evolution of water quality in Sri Lanka.

Sri Lanka has made good progress in terms of access to safe drinking water over the last few decades^4^. Over 90% of all water supply comes from improved sources of which 52% is currently supplied through pipe network that originate from both treated surface water and groundwater sources^64^. Safe drinking water is sourced from a variety of sources including hand-pumped tubewells, protected dug wells, rainwater harvesting systems. However, ~ 15% of Sri Lanka’s population is unable to access a safe water source within 200 m of their residence^5^. There are still big gaps and challenges to achieving full access to safe drinking water supply for all, especially in rural communities across Sri Lanka^10^. To align the vision with SDGs, the Government of Sri Lanka is fully committed to providing safe drinking water and improved sanitation to all its citizens^5^, for example, through piped water-supply provision. We find that the lack of routine (periodic) monitoring of groundwater quality especially for hazardous chemicals (e.g., arsenic, fluoride) at the national scale is a key limitation towards achieving the SDG 6 (Target 6.3: Improve water quality; Indicator 6.3.2: Proportion of bodies of water with good ambient water quality).

Challenges to achieving SDG 6 which will also address SDG 3 (good health and well-being) are rapid urbanization, lack of monitoring data and information, investment needs, sector governance, and greater political and financial sustainability^5^that Sri Lanka is currently facing. Due to its tropical climate consisting of distinct wet and dry seasons, availability of surface and groundwater is variable. Climate change will intensify the variability in freshwater supplies in future as projections indicate an increase in the frequency of high and extreme rainfall events with a net increase in annual rainfall^65^enhancing agricultural drought in Sri Lanka^23^. Further research is needed to establish how surface groundwater systems will respond to climate change and rainfall variability in future.

This study has looked explicitly at ambient water quality issues that are not uniform in surface and groundwater sources across Sri Lanka due to its climate, geology, urbanization and human settlements, industrial activities, and land-use practices. Surface water quality is affected by industrial effluents, agrochemicals, waste disposal and treatments and other land-use practices. Groundwater quality is generally controlled by geology, water-rock interactions in the aquifer, agricultural and other land-use practices, climate change influences recharge rates through rainfall variability, and sea level rise. In this study, we developed a groundwater multi-hazard index using various physiochemical parameters at the district level. Results from the interpolation method show that water quality is the poorest in Hambantota district located in the dry climate zone. Other districts (Puttalam, Batticaloa, Kilinochchi, Jaffna, Mullaitivu, Mannar) with high hazard index are also located in the dry and intermediate climate zones.

Rural communities are particularly vulnerable to adverse health outcomes due to poor-quality water as they are currently reliant on groundwater for drinking water. However, no national-level health data are available for analysis in this study. Strategies are planned by the government for extending water supply services beyond urban areas. To help ensure sustainable water supply and sanitation systems, rural groups or community organizations across Sri Lanka are focusing on both water quantity and quality^5^, for example, sectoral investment. Further, the NWSDB will expand water quality testing and surveillance facilities to help assure reliability in water quality in water-supply schemes^11^.

Sri Lanka needs a conjunctive water-supply system that uses both surface and groundwater sources. For example, some places may have abundant surface water, but the seasonal variability can be high too; therefore, groundwater, if available, can be developed for strategic use (i.e., in case of drought) when surface water is scare. Due to its major share in irrigated agriculture and rural drinking water supplies, water management institutions need to critically highlight the role of groundwater and specifically address in policy discussions^14^.

At the moment, little or no systematic regular or routine monitoring of groundwater takes place in the country. One-off monitored groundwater samples were collected and analyzed by the NWSDB and WRB for ambient water chemistry during any tubewell installation by the agencies. Additionally, we collated 564 groundwater quality data (Fig. S20) that were collected and analyzed by WRB in some specific districts of Sri Lanka. We collated these the national-scale water-quality data sets as secondary information from the government agencies. We find that groundwater quality is comparable between the two national agencies. These groundwater samples show different water types based on their chemical compositions (Fig. S21) – highlighting the diverse geology, aquifer types and climatic conditions in the country. This is the first comprehensive assessment of surface water and groundwater quality in Sri Lanka at the national scale. Several priority water chemicals (arsenic, fluoride, heavy metals) are not routinely analyzed at the national scale by the national agencies. We find that the Water Resources Board in Sri Lanka under the Ministry of Irrigation and Water Resources Management is undertaking pilot programs to establish groundwater monitoring network^66^. Therefore, we argue that establishing dedicated monitoring networks for collecting regular data on surface and groundwater levels and water quality will help the country track the progress in achieving SDGs (e.g., SDG 6 clean water for all) in the face of growing demand for water and climate change. Sri Lanka is a vulnerable country to climate change and sea level rise which will impact its freshwater resources, both quantity and quality. The National Adaptation Plan (NAP) for Climate Change Impacts in Sri Lanka (2016–2025), and the Nationally Determined Contributions (NDCs) have emphasized the vulnerability of the water sector and devised several measures to address water security under climate change^10^. Sri Lanka needs to implement its short-term plans of providing small-scale water supply systems such as piped distribution, small reverse osmosis, and prioritization of community tank systems rejuvenation for optimizing freshwater storage and groundwater replenishment^5,67^. In the medium-term, it should include medium-sized water-supply schemes based on surface water and groundwater sources in small towns and surrounding villages. Over the longer term, several large-scale water-supply schemes need to be implemented where priority should be given to areas where the ambient water (i.e., surface water, groundwater) quality is characterized as poor in this study and the potential risk to public health is high.

## Conclusions

This study provides the first comprehensive national-scale assessment of water quality in Sri Lanka, focusing on both surface water and groundwater. Given the critical role of water quality in achieving Sustainable Development Goal 6 (Clean Water and Sanitation), our research highlights key challenges faced by Sri Lanka. This study leverages water-quality data from key national government agencies, including 1,252 groundwater quality datasets from the National Water Supply and Drainage Board (NWSDB) and the Water Resources Board (WRB), alongside surface water quality data for the Kelani River sourced from the Central Environmental Authority and gridded data from a global database developed by the World Bank. Groundwater multi-parameter hazard index maps provide valuable tools for visualizing composite hazards and their geospatial variability across Sri Lanka. By using a data-driven approach, we created water quality indices (WQI) and groundwater-quality hazard maps, revealing significant spatial variability in water quality across the country, with millions of people exposed to substandard groundwater.

Our findings indicate that groundwater quality, particularly in rural areas where it is a major source of drinking water, is often compromised by naturally occurring contaminants and anthropogenic activities. The study estimates that approximately 3.6 to 3.8 million people are exposed to poor groundwater quality, which poses a significant public health risk. Surface water quality, especially in the Kelani River, is also threatened by industrial and agricultural pollution, further highlighting the urgent need for strategic interventions.

To achieve SDG 6 (Target 6.3: Improve water quality; Indicator 6.3.2: Proportion of bodies of water with good ambient water quality), Sri Lanka must establish a robust and routine water quality monitoring system for both surface water and groundwater. Some initiatives are currently ongoing by the Government of Sri Lanka. This will, however, require significant investment in infrastructure, training, capacity strengthening and raising public awareness. Monitoring systems should include a focus on key contaminants like fluoride, nitrate, and iron, and consider the potential exacerbation of water quality issues due to climate change and increased industrial activities.

Our results underscore the importance of prioritizing water supply improvements in regions identified as high-risk in our hazard maps. Policymakers should use these findings to guide investments in water infrastructure and to ensure equitable access to clean water, particularly in rural areas. Further research is necessary to improve the understanding of water quality trends, association between groundwater quality and recharge processes, and to develop innovative solutions for safeguarding Sri Lanka’s water resources in the face of growing environmental and climatic challenges.

## Electronic supplementary material

Below is the link to the electronic supplementary material.

Supplementary Material 1

## Data Availability

Gridded population data for Sri Lanka was downloaded from WorldPop (https://www.worldpop.org/datacatalog/); Time-series data on water quality parameters of the River Kelani was accessed from the Sri Lanka’s Central Environmental Authority (http://203.115.26.11:8881/environmentalreport/dl; free registration is required to access the time-series data under the “Status of Kalani River Basin”). The gridded surface-water quality dataset was downloaded from a global database produced and published by the World Bank (https://qualityunknown.wbwaterdata.org/quality); gridded precipitation data for Sri Lanka was downloaded from WorldClim 2.1 (http://worldclim.org); groundwater quality parameters were obtained from the National Water Supply and Drainage Board (NWSDB) and the Water Resources Board (WRB) of Sri Lanka. We do not have necessary permission from these two government agencies to make these datasets publicly available; however, for research purposes, we can provide all the processed water-quality datasets upon request made to the corresponding author of the paper.
