# Supplementary material for: Assessing the water quality hazard and challenges to achieving the freshwater goal in Sri Lanka
Source: Sci Rep. 2025 Mar 25;15:10187. doi: 10.1038/s41598-025-93845-1 (PMC11933399; doi:10.1038/s41598-025-93845-1)
Supplement: Supplementary file 1 — Supplementary Material 1 [file 41598_2025_93845_MOESM1_ESM.pdf]

Assessing the water quality hazard and challenges to achieving the freshwater goal in Sri Lanka

Mohammad Shamsudduha[1,*], Jaeyoung Lee[2], George Joseph[3], Aroha Bahuguna[3], Samantha Wijesundera[4], Sreeshankar S. Nair[3], Yi R. Hoo[3], Qiao Wang[3], Sophie C. E. Ayling[3,5]

[1] Department of Risk and Disaster Reduction, University College London, London, UK
[2] Centre for Resilience in Environment, Water and Waste, University of Exeter, UK
[3] The World Bank, Washington DC, USA
[4] The World Bank, Colombo, Sri Lanka
[5] The Bartlett Centre for Advanced Spatial Analysis, University College London, London, UK

[*]Correspondence to M. Shamsudduha: m.shamsudduha@ucl.ac.uk

Supplementary Information

Supplementary text:

S1. Sri Lanka: hydrology and climate conditions

The Democratic Socialist Republic of Sri Lanka, located in the southern tip of India between the Bay of Bengal in the southwest and the Arabian Sea in the southeast, is a humid tropical island in the Indian Ocean with distinct climate and geomorphologic conditions (Dissanayake and Chandrajith, 2018). The island country lies between the latitudes of 5°52′ and 9°50′ North and longitudes of 79°42′ and 81°52′ East. Surface elevation ranges from 0 to 2,524 m above mean sea level (msl) with elevated mountains in the south-central part of the country (Fig. S1a) around Nuwara and Kandy region. Surface elevation decreases towards the coastline around the island. The topographic configuration of the island has a centrally located highland region that is surrounded by lowland plains having a remarkable influence on the climate, hydrology, and biodiversity (Dissanayake and Chandrajith, 2018).

The national mean annual rainfall in Sri Lanka is 1880 mm with a standard deviation of 780 mm. According to the spatial distribution of mean annual rainfall (Fig. S1b), Sri Lanka is divided into 3 major climatic zones such as the wet zone (mean annual rainfall >2500 mm), dry zone (mean annual rainfall <1750 mm) and the intermediate zone (mean annual rainfall ranging from 1750 to 2500 mm). There is striking spatiotemporal variability in the mean annual rainfall across the county (Fig. S2) with the highest amount rainfall falling in June and the lowest in February. However, the second inter-monsoon between October and November most of the country including the dry zone receives much rainfall. In fact, Fig. S2 shows that the dry climate zone receives higher amount of rainfall in November and December compared to the wet climate zone.

Sri Lanka has a monsoon climate with rainfall originating mainly from monsoonal, conventional, and depressional systems influenced by orographic influences (Zubair et al., 2008). The climate in Sri Lanka can be characterized as tropical and monsoonal and is divided into four seasons: the northeast monsoon (NEM, Dec-Feb; also known as *Maha*) and southwest monsoon (SWM, May-Sep; also known as *Yala*) are major monsoon seasons that are highly depending on wind pattern; between these two periods there are another two weather patterns, namely, the first inter-monsoon (FIM, March-April) and second inter-monsoon (SIM, Oct-Nov) seasons (Jayakody, 2015). Most of the agricultural lands in Sri

Lanka are cultivated during the *Maha* season, whereas, during the *Yala* season, rice cultivation is limited to the wet zone and some parts of the dry zone that are irrigated.

S2. Geology and aquifer systems in Sri Lanka

Geomorphologically, the land surface of Sri Lanka features a complex configuration due to geology and regional tectonics (Dissanayake and Chandrajith, 2018). The island can be divided into three distinct physiographical regions namely as coastal lowlands, uplands, and highlands. The highlands are located in the southcentral part of the country in Nuwara Eliya and Norton Plains region and characterized by highland planation surfaces, erosional scarps, numerous lineaments, and faults (Panagos et al., 2011). The uplands comprise the lower highland planation levels (Kandy plain) and are characterized by erosional scarps, fold ridges and lineaments. The coastal lowlands are characterized by plateau of Miocene limestones, upwarped Pleistocene coastal plains, river plains, beach ridges. Almost 90% of the land area of Sri Lanka is drained by 103 river basins (Fig. S5) of which the River Mahaweli Ganga is the longest (335 km) in the country. Most of the other major rivers of Sri Lanka originate either from highlands or the uplands following a radial drainage pattern (Dissanayake and Chandrajith, 2018).

Geologically, Sri Lanka presents continuation of the geological characteristics of that of southern India. India and Sri Lanka are separated by a 64–137 km narrow and quite shallow (max depth of 10 m) strait, called the Palk Strait (Villholth and Rajasooriyar, 2010). Sri Lanka forms a part of the very old and stable continental mass consisting of Precambrian landmass and a small fragment of the Gondwana supercontinent (Dissanayake and Chandrajith, 2018). Nine tenth of the area of Sri Lanka is underlain by crystalline metamorphic rocks (Fig. S18) of the Precambrian age and the remaining rock mass is made up of the Miocene limestone in the north and north-western coastal regions and the Quaternary deposits along the northwestern, southern and eastern coastal regions (Cooray, 1984; Villholth and Rajasooriyar, 2010). Some sequences of sedimentary rocks are found mainly in the north and north-western coastal belt and in some isolated patches elsewhere in the island (Dissanayake and Chandrajith, 2018).

The Precambrian rocks are subdivided into three major units on the basis of their lithological, geochronological and geochemical characteristics (Cooray, 1994; Dissanayake and Chandrajith, 2018; Dissanayake and Weerasooriya, 1985): (1) The Highland Group (Highland Series) that geographically occupies a broad belt running across the center of the Island from southwest to northeast (Fig. S18). The major rock types found in this area are charnockites, quartzites, marbles, gneisses and granulites; (2) The Vijavan Complex that mostly occupies the lowlands of Sri Lanka on the northwest and eastern part of the central Highland Group belt. This complex consists of microcline biotite gneisses, hornblende gneisses, migmatites and granites; and (3) The Southwest Group that consist of cordierite gneisses, charnockites, garnetiferous gneisses, wollastonites and calc silicates rocks. The Highland Complex, which is a folded belt that is flanked by the Eastern Vijayan Complex to the east and the Wester Vijayan Complex to the west. The Highlands and Uplands are located within the wet and intermediate climate zones.

In both dry and intermediate regions of the country (Fig. S1b), groundwater is the main source of potable water. About 80% of the rural domestic water supply needs are met from groundwater by means of dug wells and tube wells according to an old report (Panabokke and Perera, 2005). Groundwater yield and quality vary considerably throughout the country depending on rainfall distribution, topography, geology, and hydrogeological conditions (Fan,

2015). Weathering of metamorphic bedrocks and fracturing have created major aquifers throughout the country (Indika et al., 2022). Highly productive aquifers are generally found in the sedimentary limestone zones extending from Puttalam to the Jaffna Peninsula and Mullativu (Fig. S18). The remaining parts of the country have local and discontinuous weathered and fissured hard rock aquifers of low productivity. Different geological and climatic conditions in the country give rise to some special geochemical characteristics to the groundwater through prolonged water–rock interaction in its highly diverse aquifer systems.

Weathering of metamorphic bedrocks and fracturing over a long geological timescale has created major aquifers throughout this land (Panabokke and Perera, 2005). Numerous studies carried out over the last 25 years by the Water Resources Board (WRB) and the National Water supply and Drainage Board (NWSDB) of Sri Lanka led to aquifer mapping at the national scale. Based on geological, geomorphological, and hydrogeological characteristics six major aquifer types have been identified and mapped by Panabokke and Perera (2005) and later on their delineation is slightly modified by others (e.g., Dissanayake and Chandrajith, 2018). The six types of aquifers are: (1) Shallow karstic aquifers of Jaffna Peninsula, (2) Deep confined aquifer, (3) Coastal sand aquifer, (4) Shallow alluvial aquifer, (5) Shallow regolith aquifer of the hard-rock region, and (6) south-western lateritic (Cabook) aquifer. The National Atlas of Sri Lanka (Karuratne, 2007; Lees and Gunatilake, 2017) published slightly different geographic distributions of aquifer systems in Sri Lanka based on an intense mapping exercise conducted by the Sri Lankan Water Resources Board between the late 1960s and early 1980s. According to this publication there are seven aquifer types or regimes (Fig. S18) in Sri Lanka. Based on available information on aquifer types, here we have mapped the aquifer systems (Fig. S18c) into seven categories: (1) Shallow karstic aquifer (1.4% area of Sri Lanka), (2) Deep confined aquifer (3.6%), (3) Shallow sandy aquifer (4.2%), (4) Laterite (cabook) aquifer (5.2), (5) Shallow alluvial aquifer (6.5%), (6) Regolith or fractured aquifer (25.5), and (7) Basement regolith aquifer (53.6). Aquifer productivity or yield varies substantially among these aquifer types. In terms of yield of the installed wells, the most productive aquifer is the shallow karstic aquifer with a median yield of 400 liters/min. The second most productive aquifer system is the deep confined aquifer with a median well yield of 210 liters/min. For the remaining five aquifer systems, the median well yields range from 30 to 36 liters/min. The basement regolith aquifer system that covers the largest part of Sri Lanka is the least productive (30 liters/min) of all aquifer types.

S3. Water quality monitoring and standards in Sri Lanka

There is a vast amount of water-quality data scattered over several organizations in Sri Lanka that operate within the water sector (Premanath, 2021). These agencies include the National Water Supply and Drainage Board (NWSDB), Water Resources Board (WRB), Central Environmental Authority (CEA), Mahaweli Authority of Sri Lanka (MASL), Irrigation Department (ID), Board of Investment (BOI), Industrial Technology Institute of Sri Lanka (ITI), International Union for Conservation of Nature (IUCN), Centre for Environmental Justice (CEJ), International Water Management Institute (IWMI) and universities. Many development and research projects carried out on water resources in Sri Lanka but not followed up with subsequent observations. Thus, there is no national-level master database on water quality of surface and ground waters.

In Sri Lanka, the National Water Supply and Drainage Board (NWSDB) is responsible for ensuring the quality of portable water that is directly supported by quality monitoring of water-supply sources. The Water Resources Board (WRB) is responsible in regulation and monitoring of groundwater quality. The Board of Investment of Sri Lanka (BOI) oversees the

wastewater management in industrial zones registered under BOI to ensure compliance of the wastewater from the common treatment plants with effluent standards. In addition, there are local authorities such as the Mahaweli Authority conducts water quality testing in their main reservoirs as a surveillance measure. The Irrigation Department recently initiated a program of monitoring the water quality in selected rivers for the purpose of riverine management. Agrarian Development Department has launched a water quality monitoring program recently with the participation of farmer organizations.

Many countries around the world established physical, chemical, and biological standards for potable water considering the importance of drinking water quality. Since 1958, the World Health Organization (WHO) has periodically published "International standards for drinking-water" and later "Guidelines for drinking-water quality". The Sri Lanka Standards Institute (SLSI) has set specification for potable water in 2013 (SLS 614, 2013). SLSI is responsible for setting standards for water quality for various uses. It is not a regulatory authority, but it promotes quality assurance across all sectors, disseminates related information and provides laboratory facilities.

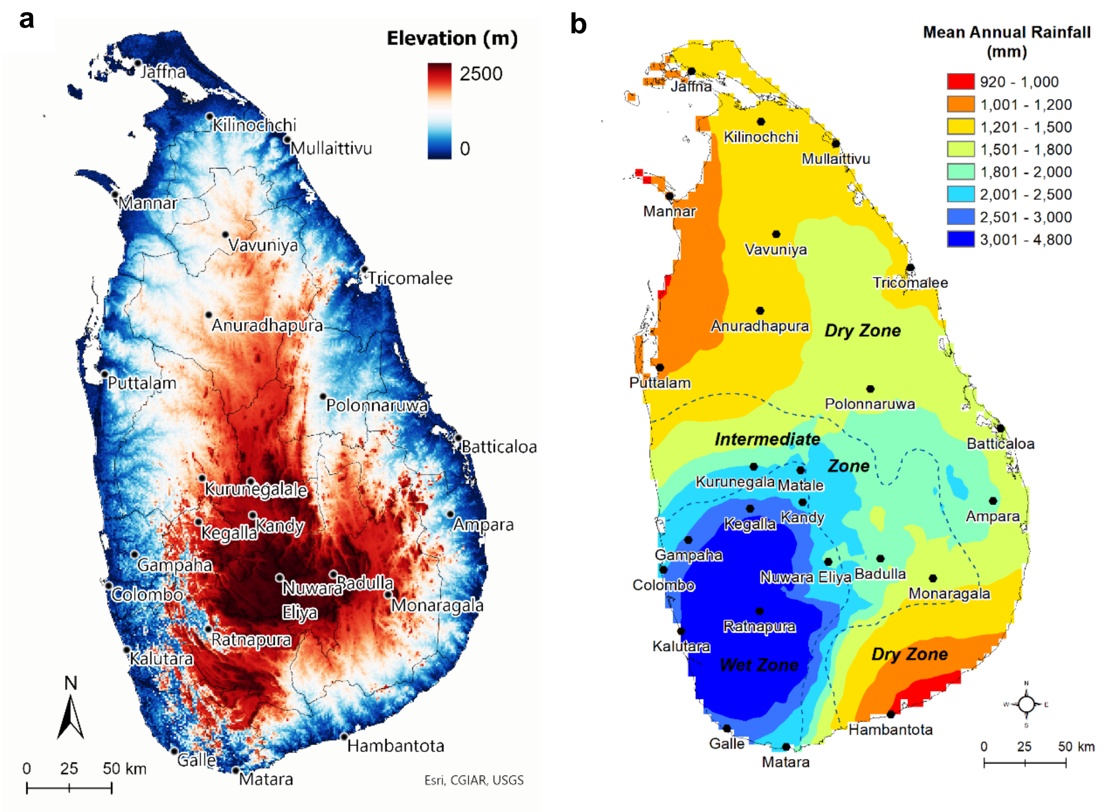

Fig. S1 Maps showing surface elevation and mean annual rainfall in Sri Lanka. **a** Elevation map (data: SRTM 90-m resolution global elevation data); and **b** long-term (1970-2000) mean annual rainfall across Sri Lanka (data source: WorldClim version 2; https://www.worldclim.org/). These original GIS maps are created in ArcGIS Desktop (v.10.8) software.

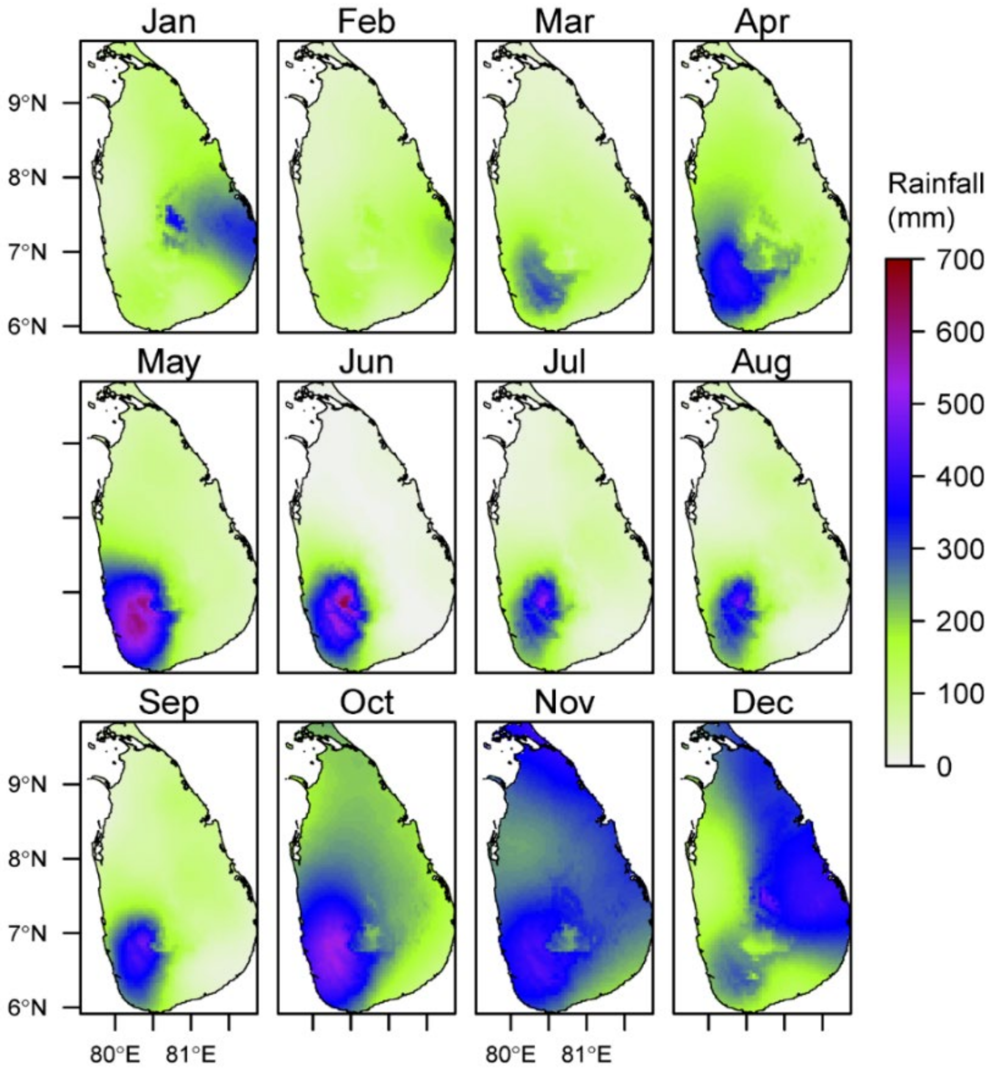

Fig. S2 Monthly climatology of rainfall in Sri Lanka. Maps shows long-term (1970-2000) mean monthly rainfall in Sri Lanka (data source: WorldClim version 2; https://www.worldclim.org/). These maps are created using R programming language.

S4. Water Quality Index method

Groundwater quality index is calculated using the Weighted Arithmetic Water Quality Index (WAWQI) method (Makubura et al., 2022) using the following formula (eq.1).

$$\mathrm{WQI} = \sum \mathrm{Q_i W_i} \;/\; \sum \mathrm{W_i} \quad \text{………. eq.1}$$

Where, Q_i is the quality rating for the ith water quality parameter; W_i is the unit weight assigned to the ith water quality parameter.

The quality rating Q_i is calculated using the following equation (eq.2):

$$Q_i = [100 * (V_i - V_0)] / (S_i - V_0) \quad \text{……… eq.2}$$

where, V_i is the actual value of the ith parameter; $V_0$is the ideal value for that parameter (see the ideal concentration in the table below); and S_i is the standard permissible value for the ith parameter.

Ideally, the unit weight W_i for each parameter is determined by: $W_i = K / S_i$ …….. eq.3

where, K is a constant, calculated as: $K = 1 / \Sigma(1 / S_i)$ ……. eq.4

However, in this study we used manual weight (Table S1) based on ranking of parameters with regard to their level of health hazard. For example, Fluoride is given the highest weight of 10 whereas phosphate is given the weight of 1. The relative importance of certain contaminants like fluoride or nitrate might be more pronounced in the specific geographical area or use case (e.g., public health concerns, regional standards).

We also assign specific ideal values (i.e., expected or targeted values for the specific water quality parameters) rather than assigning a value of 0 for all parameters. We argue that these ideal values or targeted concentrations of the selected parameters in drinking water source might be deemed as beneficial to health. For example, drinking water with zero chloride concentration may taste unpleasant and chloride ions are essential in maintaining proper fluid balance and other physiological functions in the body (Makubura et al., 2022).

The WQI values were used to assess the water quality status based on predefined ranges. These categories range from ‘Very Good’ or ‘Excellent’ to ‘Very Poor’ or ‘Unsuitable’ based on the computed WQI.

Table S1. Groundwater quality parameters used in the calculation of WQI. Drinking water standards, ideal (expected or targeted concentrations) and weights are provided.

Parameter	Unit	WHO/EPA Standard	Sri Lanka Standard	Ideal value	Manual weight
Chloride	mg/liter	250	250	100	8
Alkalinity	mg/liter	400	400	100	2
Nitrate	mg/liter	10	50	0	7
Nitrite	mg/liter	1.0	1.0	0	6
Fluoride	mg/liter	1.5	1.5	0.7	10
Phosphate	mg/liter	0.1	2.0	0	1
TDS	mg/liter	600	500	300	9
Hardness	mg/liter	500	600	100	5
Iron	mg/liter	0.3	1.0	0.1	3
Sulphate	mg/liter	200	200	0	4

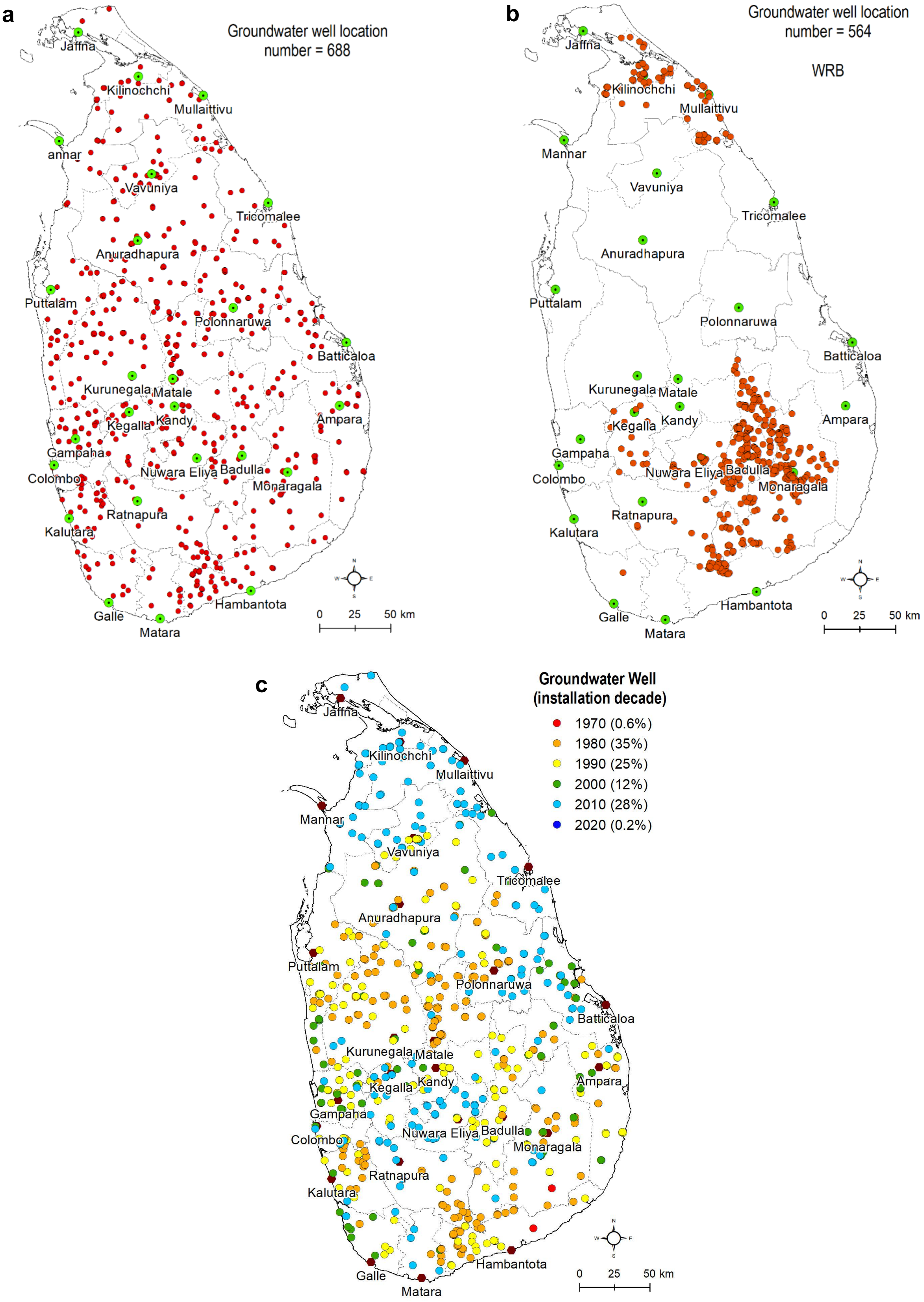

Fig. S3 Location maps of groundwater samples used in this study. a map shows location of 688 groundwater data from wells across Sri Lanka collated from the National Water Supply and Drainage Board (NWSDB); **b** map shows location of 564 groundwater data from wells in seven districts in Sri Lanka collated from the Water Resources Board (WRB); and **c** Well installation time (decade). These original GIS maps are created using ArcGIS Desktop (v.10.8) software.

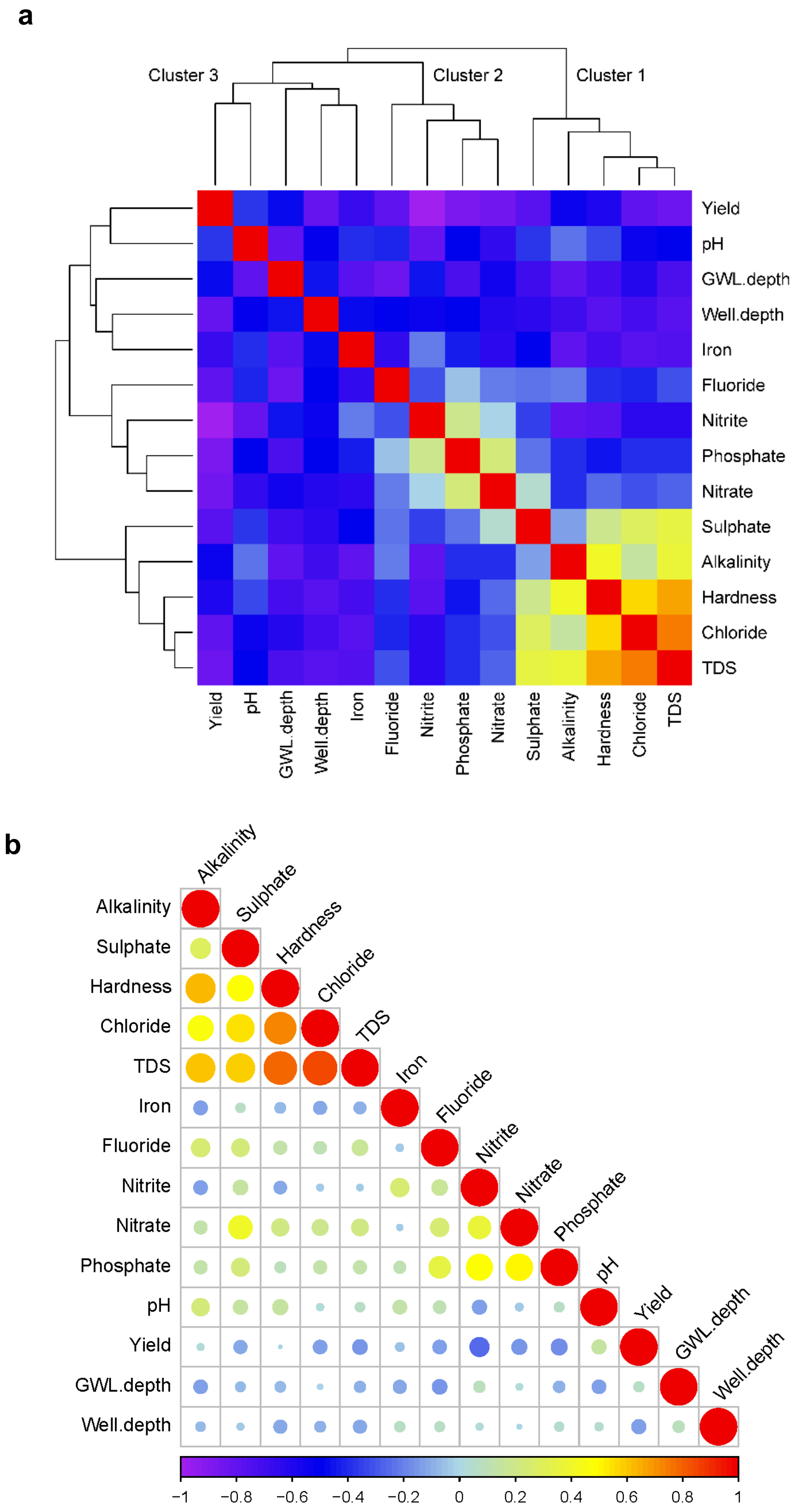

Fig. S4 Correlation matrix and clusters among groundwater quality parameters. a Correlation among various groundwater quality parameters and clusters they form among them, and **b** Visual exploratory correlation matrix plot among the same variables. Both plots are generated in R programming language using the 'corrplot' package.

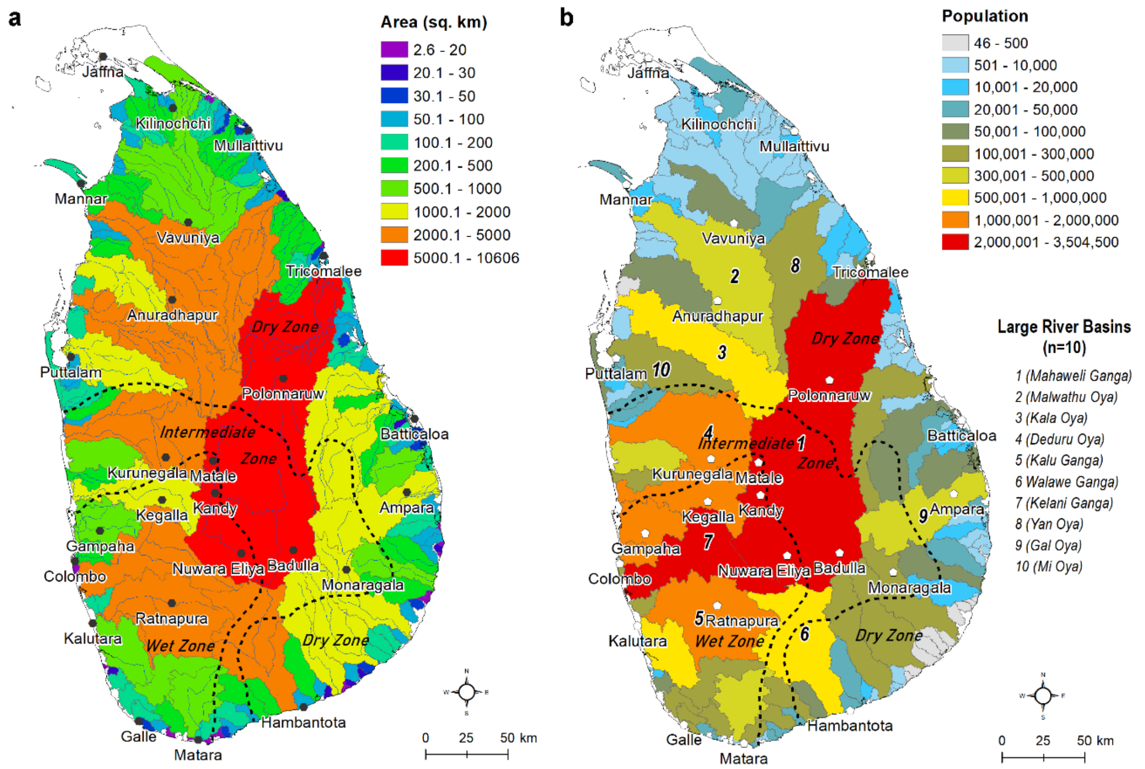

Fig. S5 Maps showing river basins and population within each basin in Sri Lanka. a Area of river basins in Sri Lanka; and **b** population within the river basins. The map also highlights the largest ten river basins with numbers from 1 to 10 where 1 being the largest of all river basins in Sri Lanka. The three climate zones, dry, intermediate, and wet, are marked by dash lines and labels on both maps. These original GIS maps are produced in ArcGIS Desktop (v.10.8) software.

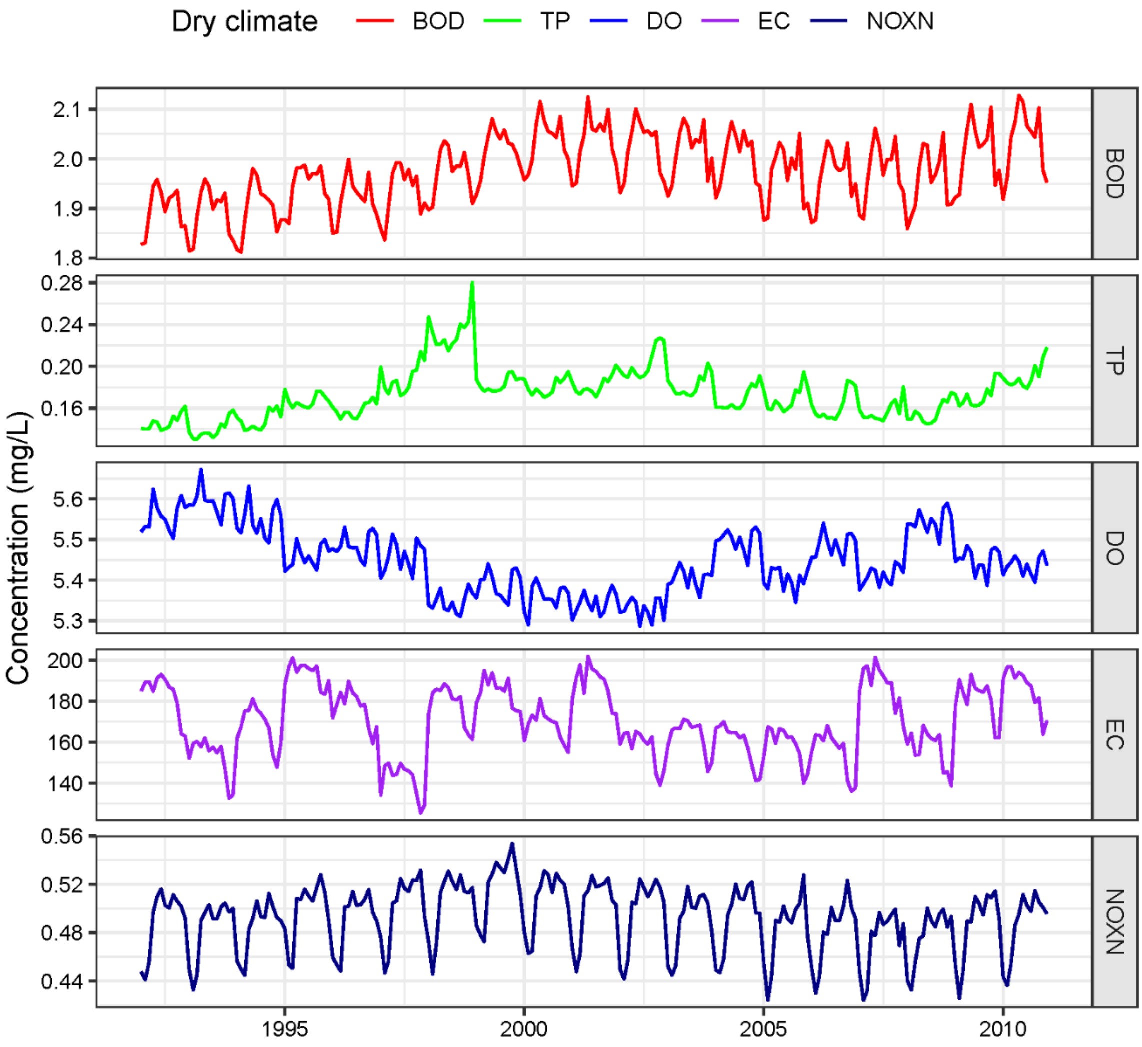

Fig. S6 Aggregated long-term (1992-2010) plots of surface water parameters across the dry climate zone in Sri Lanka. Monthly time series of BOD (Biochemical Oxygen Demand), Total Phosphorous (TP), Dissolved Oxygen (DO), Electrical Conductivity or EC (μS/cm) and NOxN or Nitrates (NO3) and Nitrites (NO2). Gridded data source: World Bank. Graphs are created in R programming language.

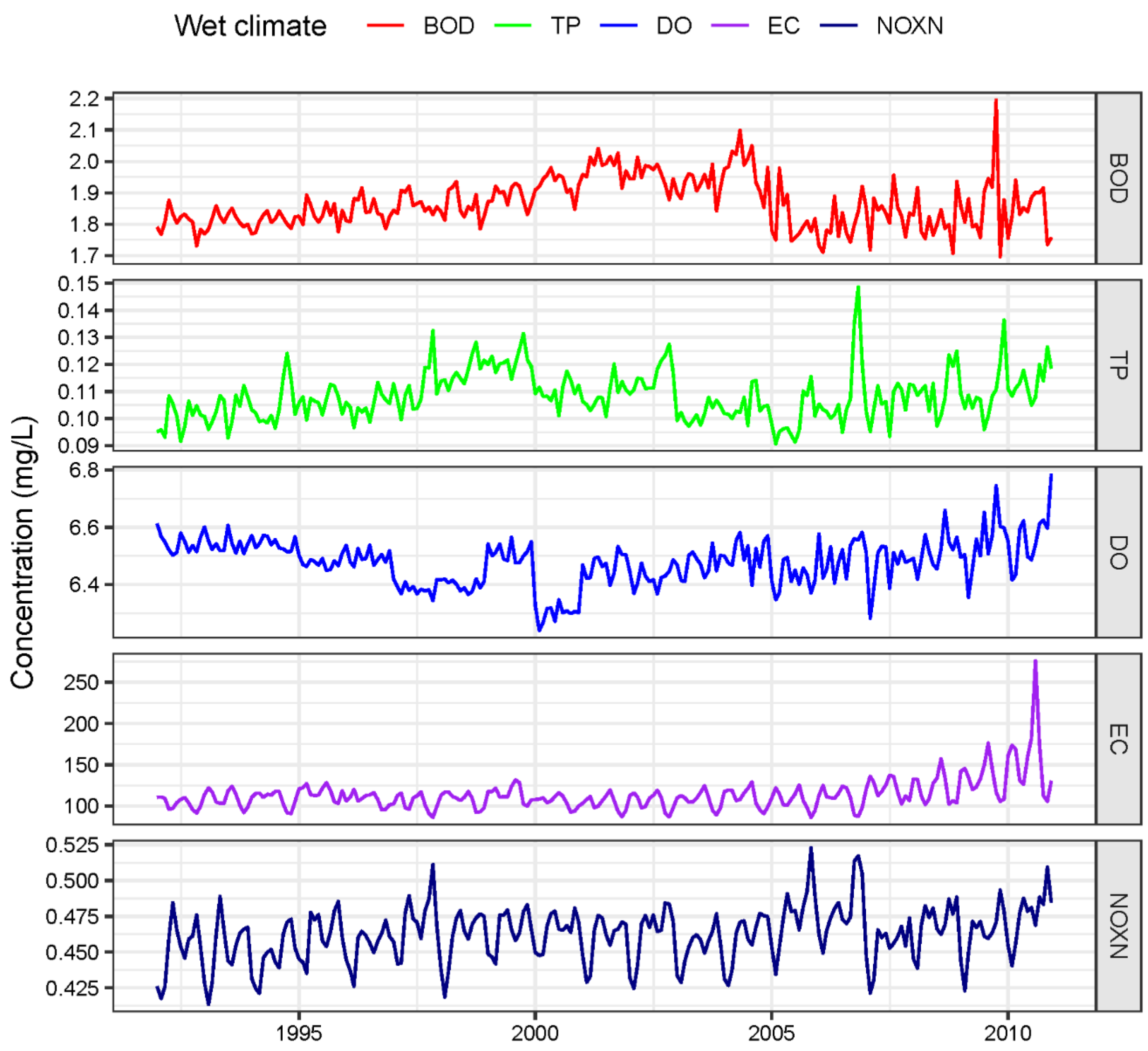

Fig. S7 Aggregated long-term (1992-2010) plots of surface water parameters across the intemediate and wet climate zones in Sri Lanka. Monthly time series of BOD (Biochemical Oxygen Demand), Total Phosphorous (TP), Dissolved Oxygen (DO), Electrical Conductivity or EC (μS/cm) and NOxN or Nitrates (NO3) and Nitrites (NO2). Gridded data source: World Bank. Graphs are created in R programming language.

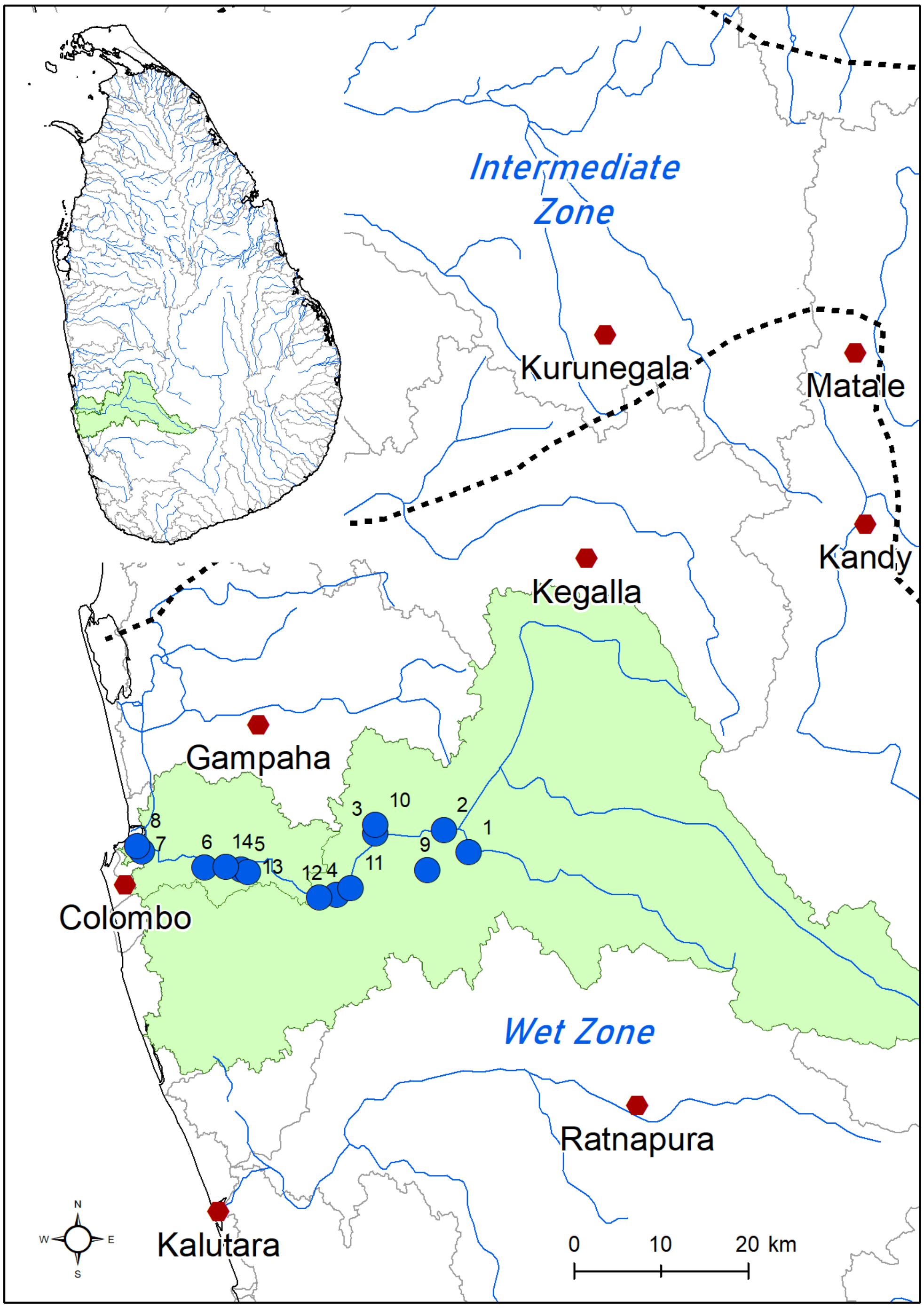

Fig. S8 Location map of surface water-quality data from the Kelani River basin. The monitoring points are: Thalduwa Bridge (1 - upstream), Seethawake Ferry (2 - upstream), Pugoda Ferry (3 - upstream), Hanwella Bridge (4 - mid-stream), Kaduwela Bridge (5 – downstream), Welivita Bridge (6 - downstream), New Bridge Peliyagoda (7 - downstream), Victoria Bridge (8 - downstream), Eswathu Oya (9 - upstream), Pugoda Ela (10 - upstream), Wak Oya (11 - mid-stream), Pusseli Oya (12 - mid-stream), Maha Ela (13 - downstream), and Raggahawatte Ela (14 - downstream). This is an original GIS map that is produced in ArcGIS Desktop (v.10.8) software.

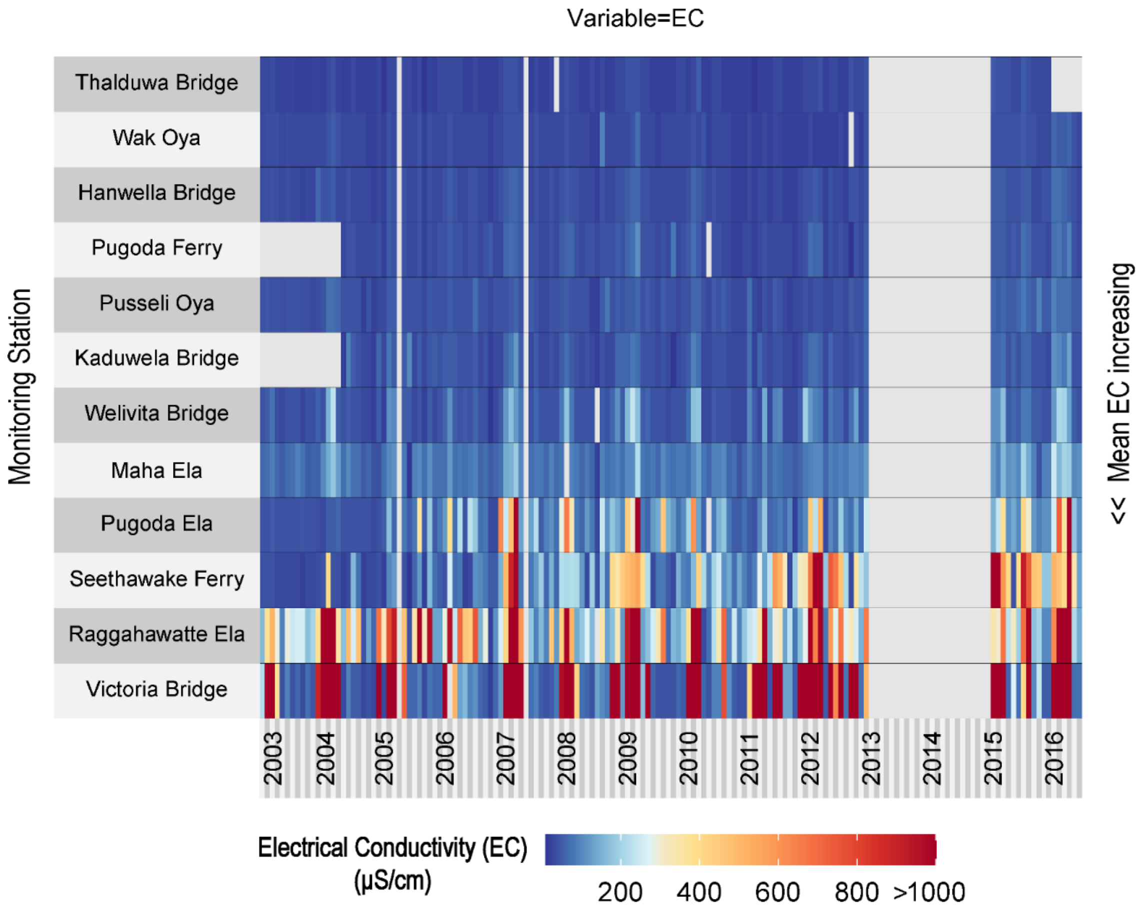

Fig. S9 Long-term (2003-2016) changes in Electrical Conductivity (EC) in 12 monitoring sites along Kelani River in Sri Lanka. Stations are arranged from top to bottom of the heatmap according to their mean parameter values. Gaps in the monthly time-series data (2013-2014) are shown in light grey shade. Data source: The Central Environmental Authority (http://203.115.26.11:8881/environmentalreport/dl). The monitoring points are: Thalduwa Bridge (1 - upstream), Seethawake Ferry (2 - upstream), Pugoda Ferry (3 - upstream), Hanwella Bridge (4 - mid-stream), Kaduwela Bridge (5 – downstream), Welivita Bridge (6 - downstream), New Bridge Peliyagoda (7 - downstream), Victoria Bridge (8 - downstream), Eswathu Oya (9 - upstream), Pugoda Ela (10 - upstream), Wak Oya (11 - mid-stream), Pusseli Oya (12 - mid-stream), Maha Ela (13 - downstream), and Raggahawatte Ela (14 - downstream). This heatmap is created using R programming language.

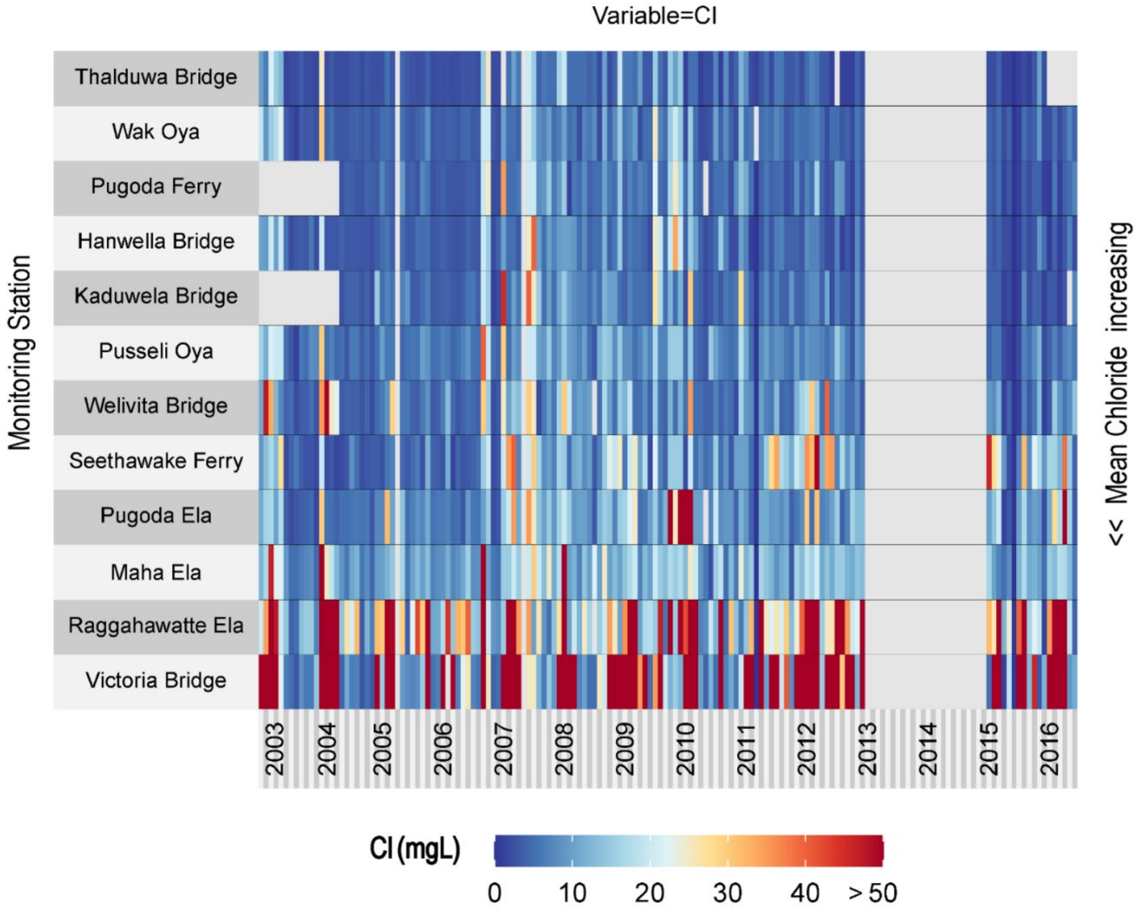

Fig. S10 Long-term (2003-2016) changes in Chloride (Cl) in 12 monitoring sites along Kelani River in Sri Lanka. Stations are arranged from top to bottom of the heatmap according to their mean parameter values. Gaps in the monthly time-series data (2013-2014) are shown in light grey shade. Data source: The Central Environmental Authority (http://203.115.26.11:8881/environmentalreport/dl). The monitoring points are: Thalduwa Bridge (1 - upstream), Seethawake Ferry (2 - upstream), Pugoda Ferry (3 - upstream), Hanwella Bridge (4 - mid-stream), Kaduwela Bridge (5 – downstream), Welivita Bridge (6 - downstream), New Bridge Peliyagoda (7 - downstream), Victoria Bridge (8 - downstream), Eswathu Oya (9 - upstream), Pugoda Ela (10 - upstream), Wak Oya (11 - mid-stream), Pusseli Oya (12 - mid-stream), Maha Ela (13 - downstream), and Raggahawatte Ela (14 - downstream). This heatmap is created using R programming language.

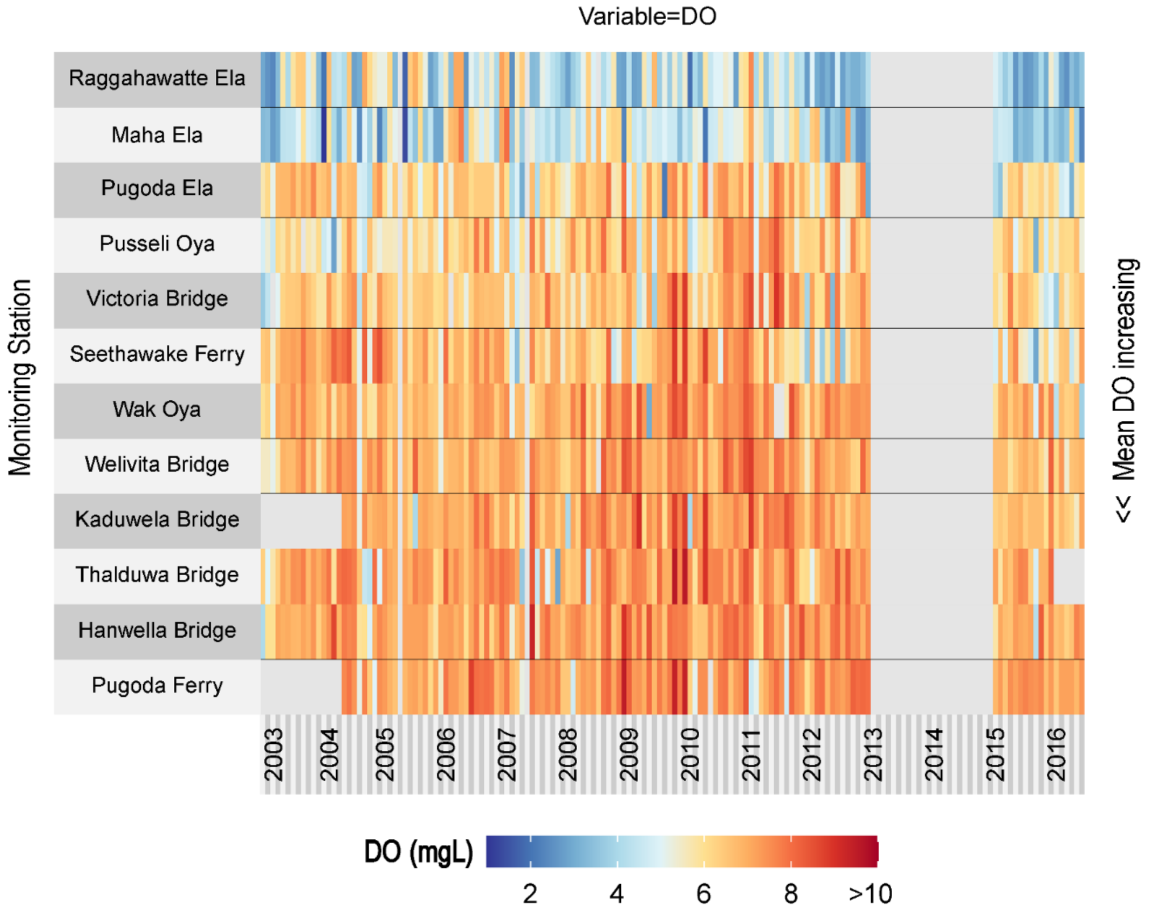

Fig. S11 Long-term (2003-2016) changes in Dissolved Oxygen (DO) in 12 monitoring sites along Kelani River in Sri Lanka. Stations are arranged from top to bottom of the heatmap according to their mean parameter values. Gaps in the monthly time-series data (2013-2014) are shown in light grey shade. Data source: The Central Environmental Authority (http://203.115.26.11:8881/environmentalreport/dl). The monitoring points are: Thalduwa Bridge (1 - upstream), Seethawake Ferry (2 - upstream), Pugoda Ferry (3 - upstream), Hanwella Bridge (4 - mid-stream), Kaduwela Bridge (5 – downstream), Welivita Bridge (6 - downstream), New Bridge Peliyagoda (7 - downstream), Victoria Bridge (8 - downstream), Eswathu Oya (9 - upstream), Pugoda Ela (10 - upstream), Wak Oya (11 - mid-stream), Pusseli Oya (12 - mid-stream), Maha Ela (13 - downstream), and Raggahawatte Ela (14 - downstream). This heatmap is created using R programming language.

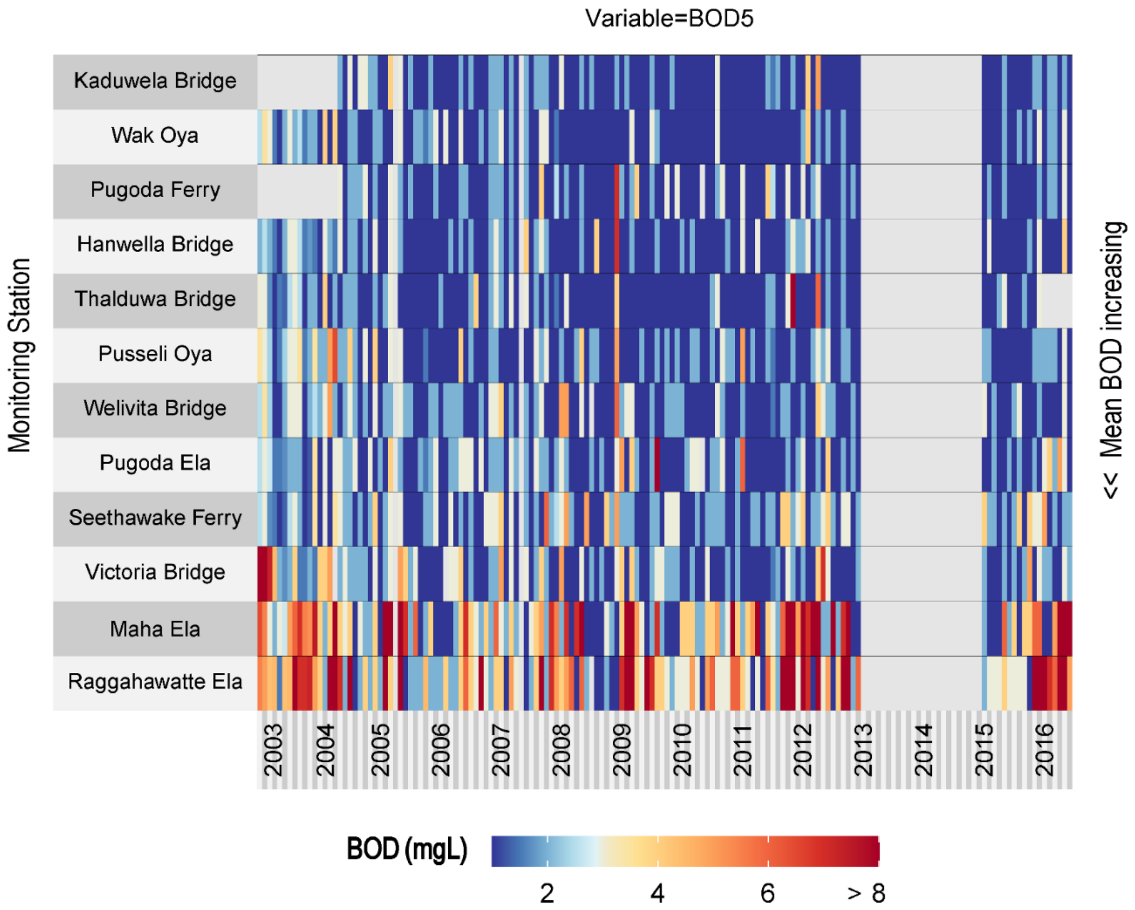

Fig. S12 Long-term (2003-2016) changes in Biochemical Oxygen Demand (BOD) in 12 monitoring sites along Kelani River in Sri Lanka. Stations are arranged from top to bottom of the heatmap according to their mean parameter values. Gaps in the monthly time-series data (2013-2014) are shown in light grey shade. Data source: The Central Environmental Authority (http://203.115.26.11:8881/environmentalreport/dl). The monitoring points are: Thalduwa Bridge (1 - upstream), Seethawake Ferry (2 - upstream), Pugoda Ferry (3 - upstream), Hanwella Bridge (4 - mid-stream), Kaduwela Bridge (5 – downstream), Welivita Bridge (6 - downstream), New Bridge Peliyagoda (7 - downstream), Victoria Bridge (8 - downstream), Eswathu Oya (9 - upstream), Pugoda Ela (10 - upstream), Wak Oya (11 - mid-stream), Pusseli Oya (12 - mid-stream), Maha Ela (13 - downstream), and Raggahawatte Ela (14 - downstream). This heatmap is created using R programming language.

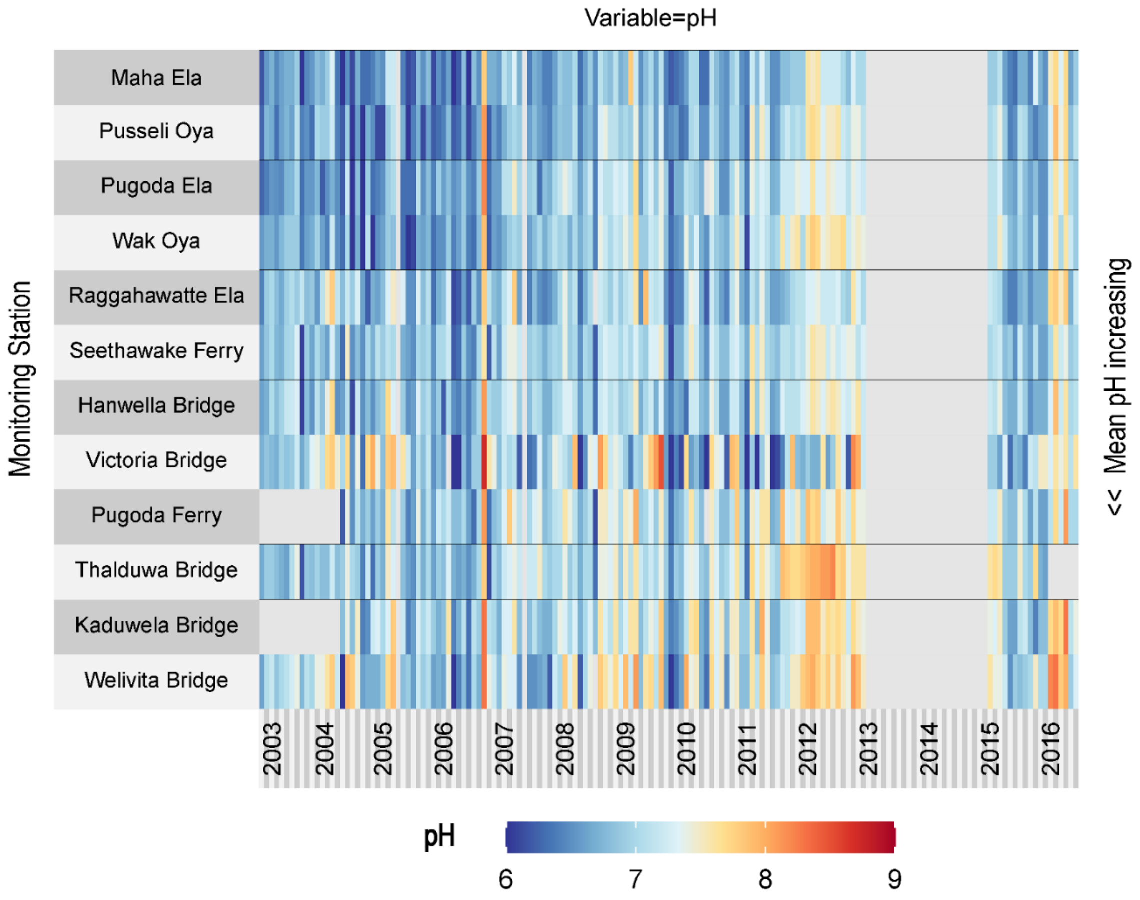

Fig. S13 Long-term (2003-2016) changes in water pH in 12 monitoring sites along Kelani River in Sri Lanka. Stations are arranged from top to bottom of the heatmap according to their mean parameter values. Gaps in the monthly time-series data (2013-2014) are shown in light grey shade. Data source: The Central Environmental Authority (http://203.115.26.11:8881/environmentalreport/dl). The monitoring points are: Thalduwa Bridge (1 - upstream), Seethawake Ferry (2 - upstream), Pugoda Ferry (3 - upstream), Hanwella Bridge (4 - mid-stream), Kaduwela Bridge (5 – downstream), Welivita Bridge (6 - downstream), New Bridge Peliyagoda (7 - downstream), Victoria Bridge (8 - downstream), Eswathu Oya (9 - upstream), Pugoda Ela (10 - upstream), Wak Oya (11 - mid-stream), Pusseli Oya (12 - mid-stream), Maha Ela (13 - downstream), and Raggahawatte Ela (14 - downstream). This heatmap is created using R programming language.

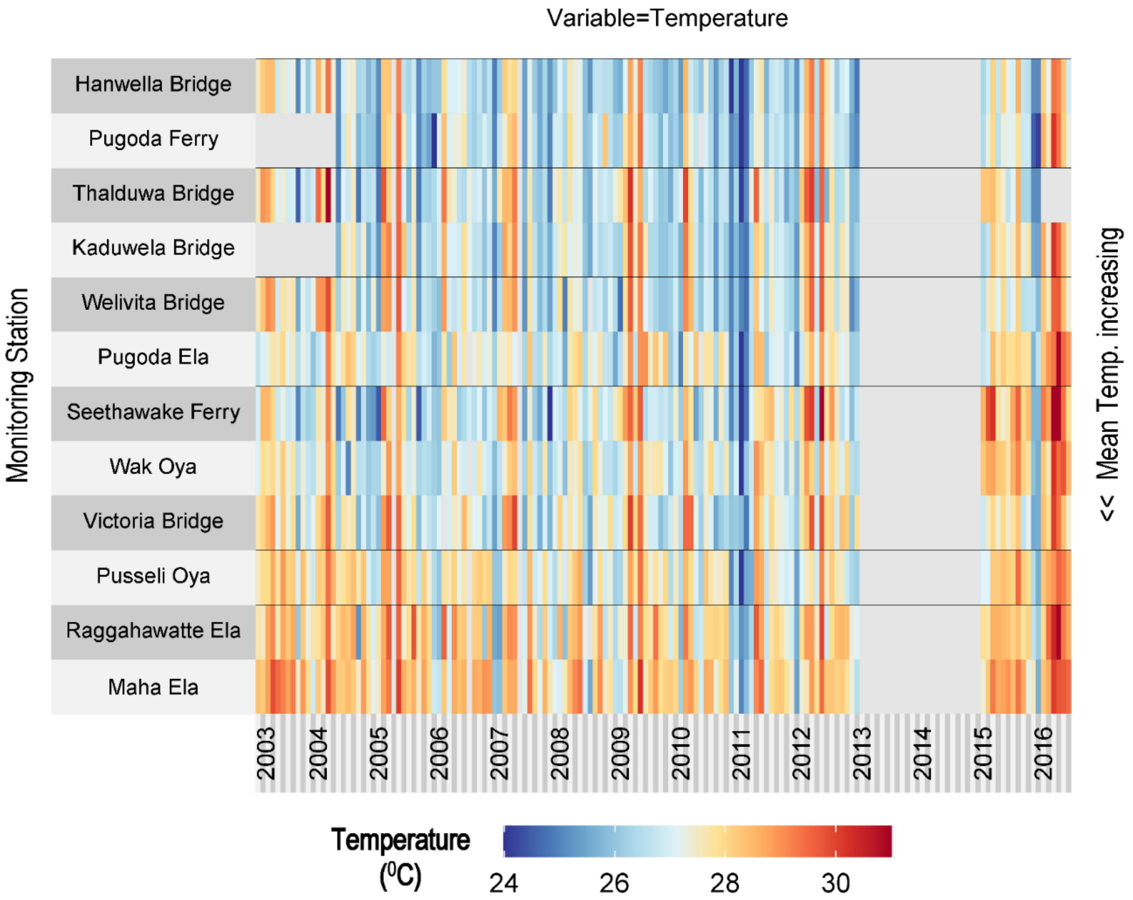

Fig. S14 Long-term (2003-2016) changes in water Temperature in 12 monitoring sites along Kelani River in Sri Lanka. Stations are arranged from top to bottom of the heatmap according to their mean parameter values. Gaps in the monthly time-series data (2013-2014) are shown in light grey shade. Data source: The Central Environmental Authority (http://203.115.26.11:8881/environmentalreport/dl). The monitoring points are: Thalduwa Bridge (1 - upstream), Seethawake Ferry (2 - upstream), Pugoda Ferry (3 - upstream), Hanwella Bridge (4 - mid-stream), Kaduwela Bridge (5 – downstream), Welivita Bridge (6 - downstream), New Bridge Peliyagoda (7 - downstream), Victoria Bridge (8 - downstream), Eswathu Oya (9 - upstream), Pugoda Ela (10 - upstream), Wak Oya (11 - mid-stream), Pusseli Oya (12 - mid-stream), Maha Ela (13 - downstream), and Raggahawatte Ela (14 - downstream). This heatmap is created using R programming language.

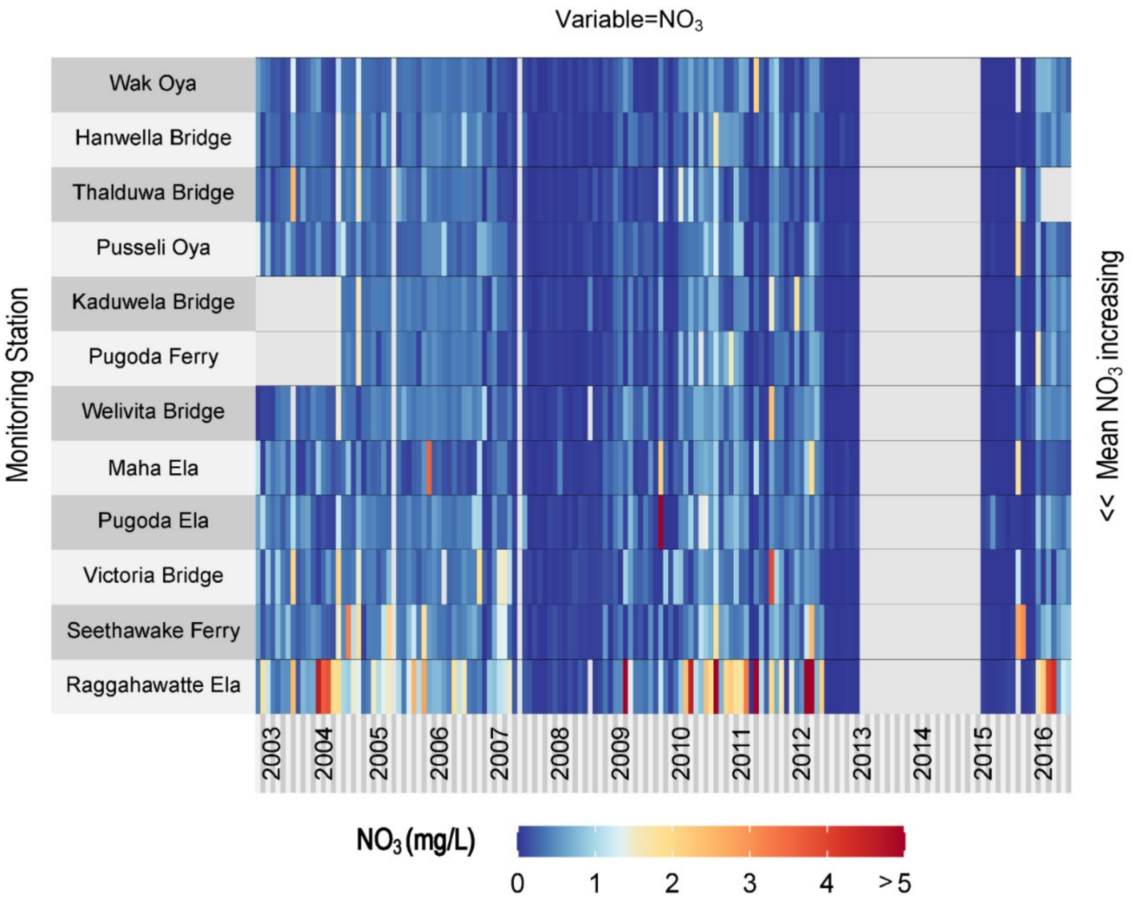

Fig. S15 Long-term (2003-2016) changes in nitrogen concentrations (NO_3) in 12 monitoring sites along Kelani River in Sri Lanka. Stations are arranged from top to bottom of the heatmap according to their mean parameter values. Gaps in the monthly time-series data (2013-2014) are shown in light grey shade. Data source: The Central Environmental Authority (http://203.115.26.11:8881/environmentalreport/dl). The monitoring points are: Thalduwa Bridge (1 - upstream), Seethawake Ferry (2 - upstream), Pugoda Ferry (3 - upstream), Hanwella Bridge (4 - mid-stream), Kaduwela Bridge (5 – downstream), Welivita Bridge (6 - downstream), New Bridge Peliyagoda (7 - downstream), Victoria Bridge (8 - downstream), Eswathu Oya (9 - upstream), Pugoda Ela (10 - upstream), Wak Oya (11 - mid-stream), Pusseli Oya (12 - mid-stream), Maha Ela (13 - downstream), and Raggahawatte Ela (14 - downstream). This heatmap is created using R programming language.

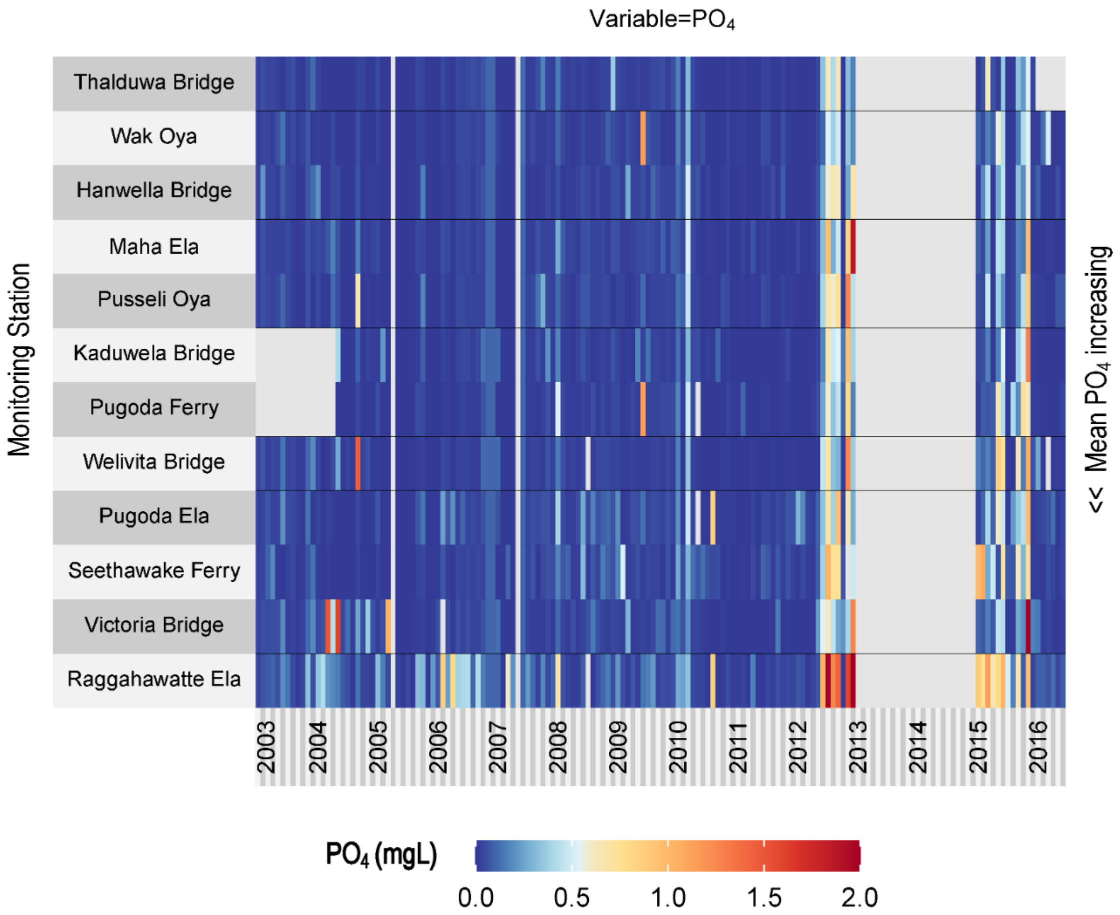

Fig. S16 Long-term (2003-2016) changes in phosphorous concentrations (PO_4) in 12 monitoring sites along Kelani River in Sri Lanka. Stations are arranged from top to bottom of the heatmap according to their mean parameter values. Gaps in the monthly time-series data (2013-2014) are shown in light grey shade. Data source: The Central Environmental Authority (http://203.115.26.11:8881/environmentalreport/dl). The monitoring points are: Thalduwa Bridge (1 - upstream), Seethawake Ferry (2 - upstream), Pugoda Ferry (3 - upstream), Hanwella Bridge (4 - mid-stream), Kaduwela Bridge (5 – downstream), Welivita Bridge (6 - downstream), New Bridge Peliyagoda (7 - downstream), Victoria Bridge (8 - downstream), Eswathu Oya (9 - upstream), Pugoda Ela (10 - upstream), Wak Oya (11 - mid-stream), Pusseli Oya (12 - mid-stream), Maha Ela (13 - downstream), and Raggahawatte Ela (14 - downstream). This heatmap is created using R programming language.

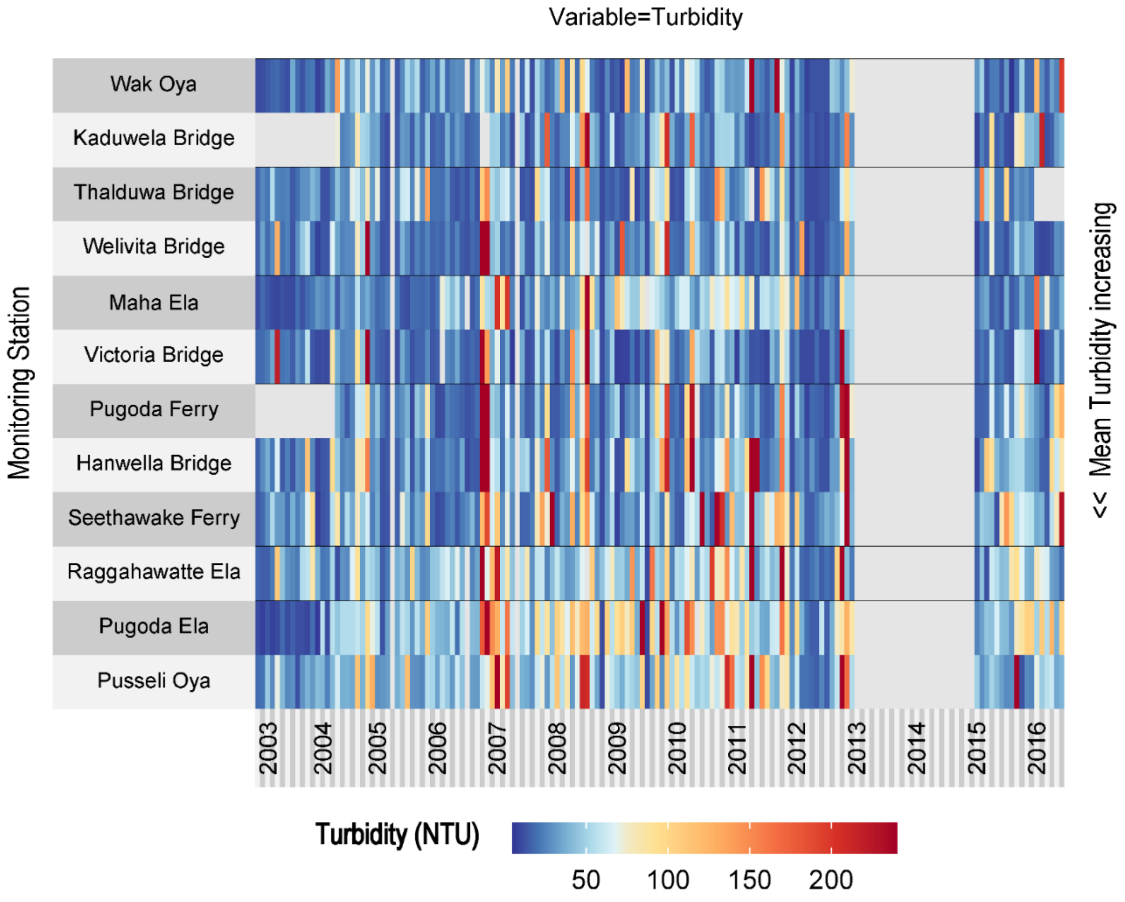

Fig. S17 Long-term (2003-2016) changes in water turbidity (TURB) in 12 monitoring sites along Kelani River in Sri Lanka. Stations are arranged from top to bottom of the heatmap according to their mean parameter values. Gaps in the monthly time-series data (2013-2014) are shown in light grey shade. Data source: The Central Environmental Authority (http://203.115.26.11:8881/environmentalreport/dl). The monitoring points are: Thalduwa Bridge (1 - upstream), Seethawake Ferry (2 - upstream), Pugoda Ferry (3 - upstream), Hanwella Bridge (4 - mid-stream), Kaduwela Bridge (5 – downstream), Welivita Bridge (6 - downstream), New Bridge Peliyagoda (7 - downstream), Victoria Bridge (8 - downstream), Eswathu Oya (9 - upstream), Pugoda Ela (10 - upstream), Wak Oya (11 - mid-stream), Pusseli Oya (12 - mid-stream), Maha Ela (13 - downstream), and Raggahawatte Ela (14 - downstream). This heatmap is created using R programming language.

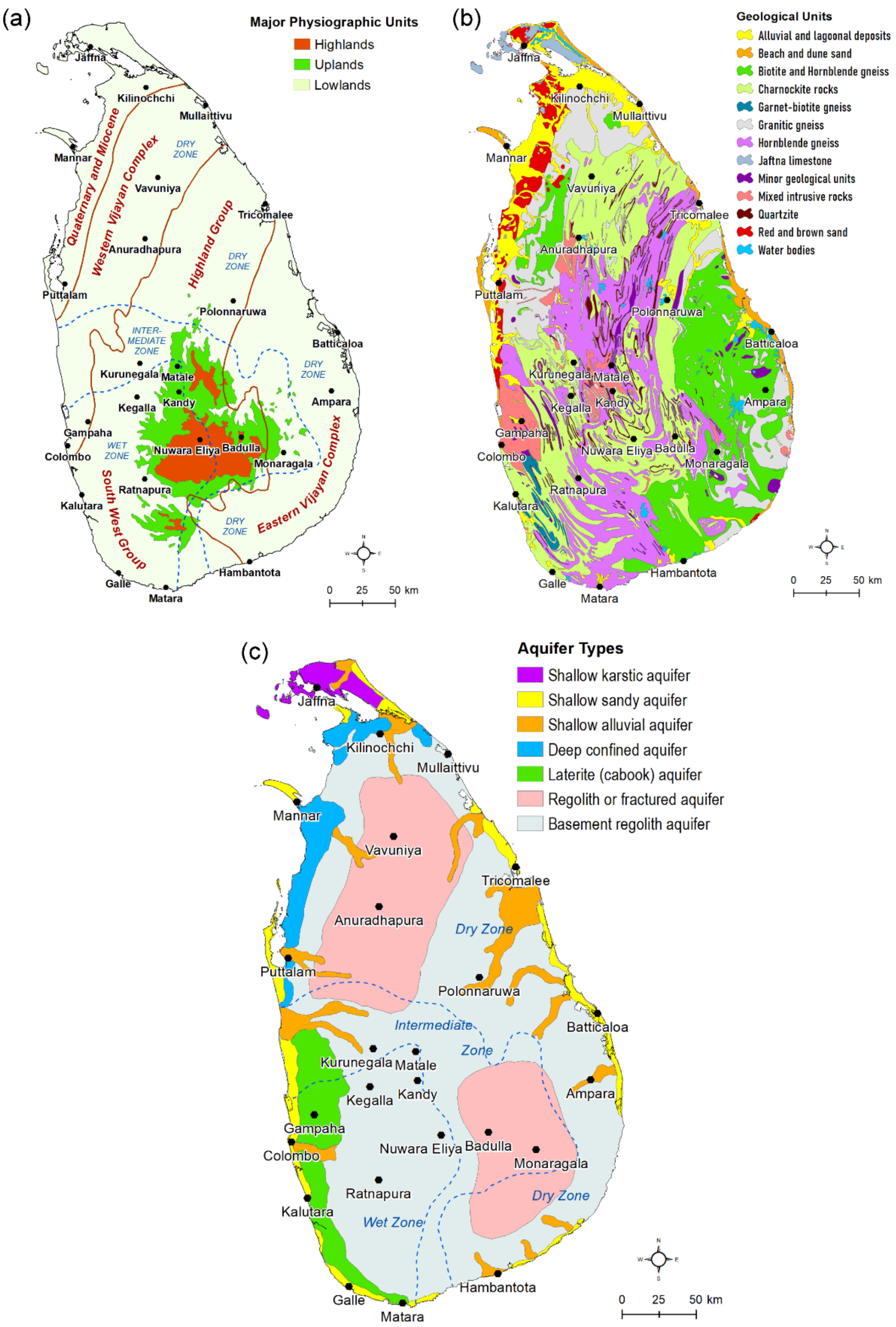

Fig. S18 Geology and aquifer systems in Sri Lanka. a Major physiographic units and geological divisions in Sri Lanka; **b** detailed geological formations; and **c** geographic distribution of various aquifer systems in Sri Lanka (note: map compiled and digitized from several sources including (Dissanayake and Chandrajith, 2018; Dissanayake and Weerasooriya, 1985; Karuratne, 2007; Panabokke, 2007; Panabokke and Perera, 2005). These original GIS maps are created using ArcGIS Desktop (v.10.8) software.

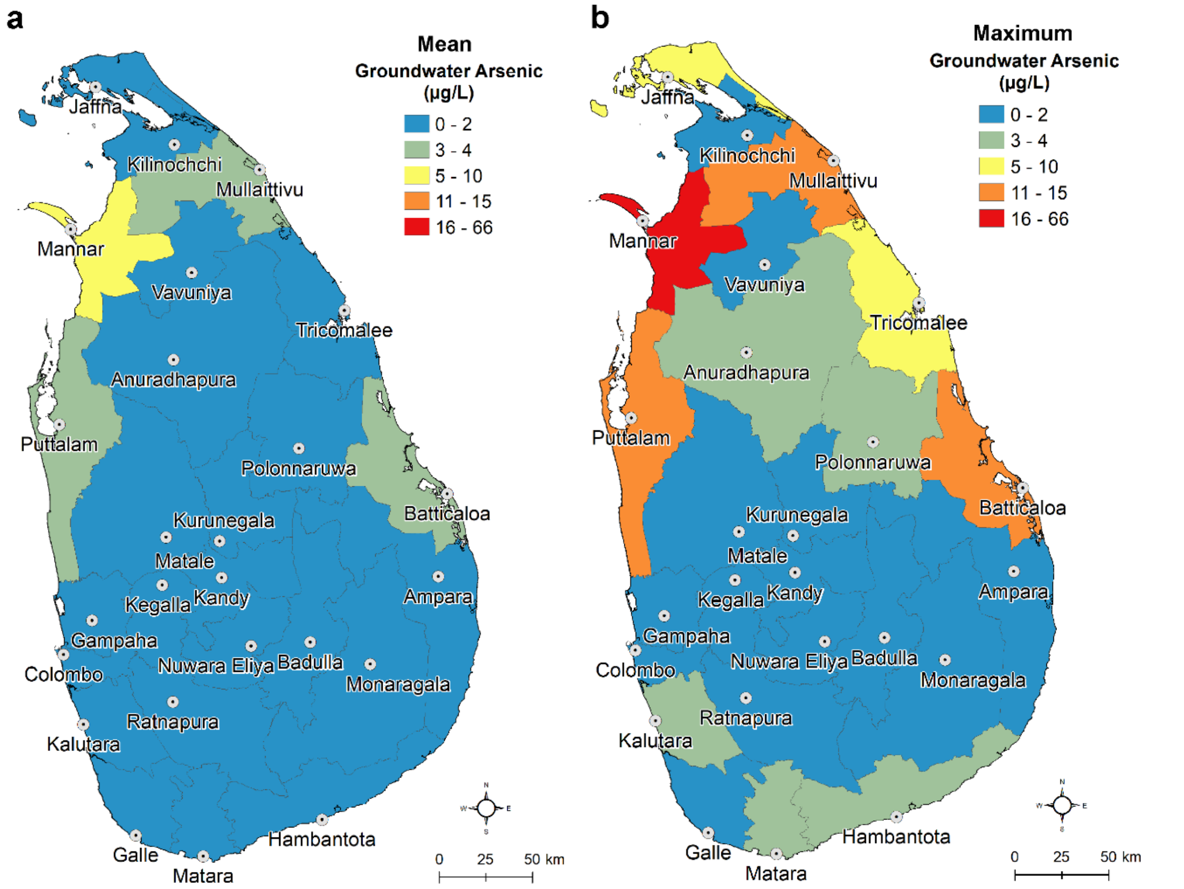

Fig. S19 Aggregated groundwater arsenic concentrations in the districts of Sri Lanka. **a** The maximum groundwater arsenic concentrations in the 25 districts of Sri Lanka; and **b** The average groundwater arsenic concentrations in the 25 districts of Sri Lanka. These statistics derive from a national-scale study of 1,304 groundwatre-quality data points collected and analysed by Herath et al. (2017). Water samples were collected from the wells in all districts in Sri Lanka from 2010 to 2014. These original GIS maps are created using ArcGIS Desktop (v.10.8) software.

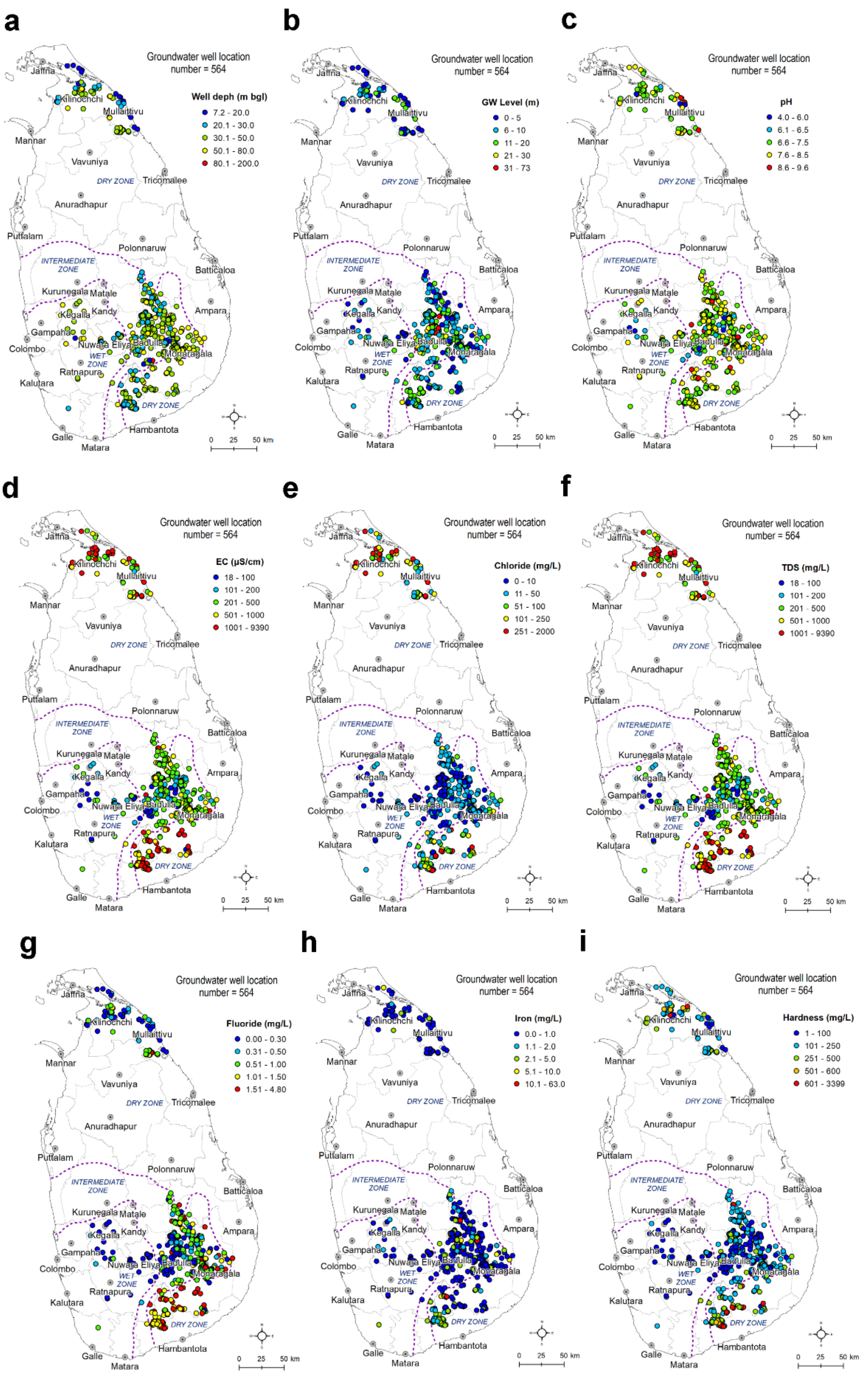

Fig. S20 Groundwater physiochemical parameters in Sri Lanka at 564 wells from the Water Resources Board database. a Well depth; **b** Groundwater levels in wells; **c** Groundwater pH levels; **d** Groundwater Electrical Conductivity (EC); **e** Chloride; **f** Total Dissolved Solids (TDS); **g** Fluoride; **h** Total iron; and **i** Water hardness. These original GIS maps are created using ArcGIS Desktop (v.10.8) software.

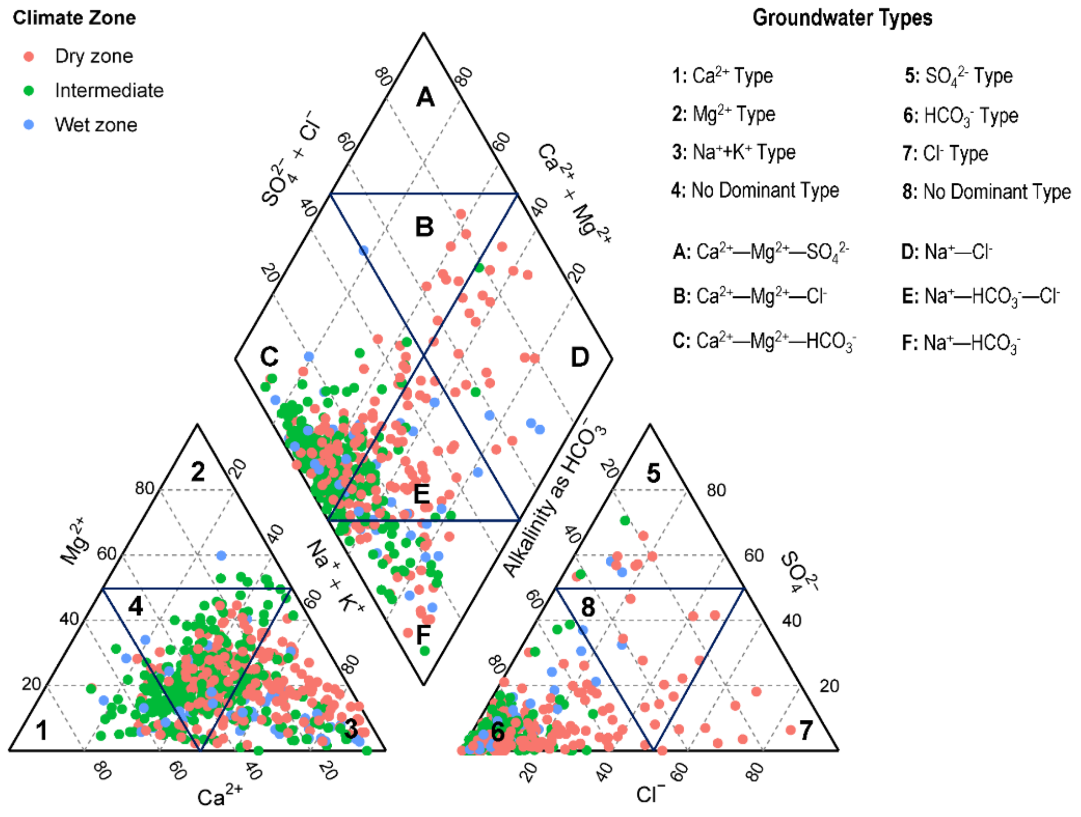

Fig. S21 Piper diagram showing groundwater classification based on chemical composition. Total 564 groundwater data are used in this diagram to show a range of water types found within the seven districts that are in three climate zones. Even though the types of groundwater are not strikingly distinction between the climate zones, the samples from dry zones show more Na-Cl type of water. This Piper diagram is created using the R programming language.

Table S2. Meta-data analysis of water-quality literature reviewed in this study.

No.	Journal Title	Year	Water Source	Water Quality Parameters	Geographical Locations	Citation
1	Determinants of water quality, availability and use in Kurunegala, Sri Lanka	1990	GW (Groundwater)	Faecal Coliform	Kurunegala district	(Mertens et al., 1990)
2	A solution to scaling problems and operational difficulties in Mihinthale drinking water supply scheme.	1998	GW	Colour, pH, Turbidity, Alkalinity, Hardness, F, Fe, TDS, Cl and EC	Anuradhapura district	(Wijesinghe and Mahanama, 1998)
3	Conceptual model of the evolution of groundwater quality at the wet zone in Sri Lanka	1999	GW	Mg, Na, Sulphate, Bicarbonate, Si and EC	Kandy and Kurunegala district	(Song et al., 1999)
4	Nitrate pollution in ground water of Kalpitiya: An evaluation of the content of nitrates in the water and food items cultivated in the area	2000	GW	Nitrate, Nitrite	Puttalam district (Kalpitiya)	(Liyanage et al., 2000)
5	Chronic kidney diseases of uncertain etiology (CKDue) in Sri Lanka: geographic distribution and environmental implications	2011	GW	Cd, Pb, Al, Ni, Cu, Zn, As and U	Whole country	(Chandrajith et al., 2011)
6	Factors controlling fluoride contents of groundwater in north-central and northwestern Sri Lanka	2011	GW	F, Na, K, Ca, Mg, Fe, Mn, Bicarbonate, pH, EC, and Total Hardness	Kurunegala, Anuradhapura, Matale districts	(Young et al., 2011)
7	Groundwater stress and vulnerability in rural coastal aquifers under competing demands: a case study from Sri Lanka	2011	GW	Cl and Nitrate	Puttalam district	(Jayasekera et al., 2011)
8	Vulnerability of Coastal Aquifers Due to Nutrient Pollution from Agriculture: Kalpitiya, Sri Lanka	2011	GW	Nitrate	Puttalam district	(Jayasingha et al., 2011)
9	Spatial distribution of fluoride in groundwater of Sri Lanka	2012	GW	F	Whole country	(Chandrajith et al., 2012)
10	Escalating chronic kidney diseases of multi-factorial origin in Sri Lanka: causes, solutions, and recommendations	2014	GW	As, Cd, F and Pesticides	Whole country	(Wimalawansa, 2014)
11	Chronic kidney disease of unknown aetiology and ground-water ionicity: study based on Sri Lanka	2015	GW	As and Cd	Polonnaruwa and Anuradhapura districts (North Central Province)	(Dharma-wardana et al., 2015)
12	An approach to delineate groundwater recharge potential sites in Ambalantota, Sri Lanka using GIS techniques	2016	GW	Discussed about the recharge potential ground water in Sri Lanka	Hambantota district	(Senanayake et al., 2016)
13	Assessing causes of quality deterioration of	2016	GW	pH, EC, Temperature, Total Alkalinity,	Puttalam district	(Edirisinghe et al., 2016)

No.	Journal Title	Year	Water Source	Water Quality Parameters	Geographical Locations	Citation
	groundwater in Puttalam, Sri Lanka, using isotope and hydrochemical tools			TDS and DO		
14	Drinking water quality and chronic kidney disease of unknown etiology (CKDu): synergic effects of fluoride, cadmium and hardness of water	2016	GW	F, Al, Cd, As, and Total Hardness	Polonnaruwa and Anuradhapura districts (North Central Province)	(Wasana et al., 2016)
15	Has irrigated water from Mahaweli River contributed to the kidney disease of uncertain etiology in the dry zone of Sri Lanka?	2016	GW	Cd, Se, As, and Mo	Nuwara Eliya district	(Diyabalanage et al., 2016)
16	The role of ions, heavy metals, fluoride, and agrochemicals: critical evaluation of potential aetiological factors of chronic kidney disease of multifactorial origin (CKDmfo/CKDu) and recommendations for its eradication	2016	GW	Cd, Pb, As, F and other agrochemicals	Whole country	(Wimalawansa, 2016)
17	Assessment of the Efficacy of Home Remedial Methods to Improve Drinking Water Quality in Two Major Aquifer Systems in Jaffna Peninsula, Sri Lanka	2017	GW	pH, DO, EC, TDS, Total Hardness, Salinity, Temperature, TS, COD, Oil and Grease, Nitrate, and TP	Jaffna district	(Wijeyaratne and Subanky, 2017)
18	Potential risk of drinking water to human health in Sri Lanka	2017	GW	F, Nitrate, Hardness, As, Al, Mn	Whole country	(Herath et al., 2017)
19	Arsenic, cadmium, lead, and chromium in well water, rice, and human urine in Sri Lanka in relation to chronic kidney disease of unknown etiology	2018	GW	As, Cd, Pb, and Cr	Whole country	(S. Herath et al., 2018)
20	Factors associated with Chronic Kidney Disease of unknown aetiology (CKDu) in North Central Province of Sri Lanka: a comparative analysis of drinking water samples	2018	GW	Cl, F, Nitrate, Phosphate, Ca, Mg, and Na	Polonnaruwa and Anuradhapura districts (North Central Province)	(Paranagama et al., 2018)
21	Geochemical and isotopic evidences from groundwater and surface water for understanding of natural contamination in chronic kidney disease of unknown etiology (CKDu) endemic zones in Sri Lanka	2018	GW	pH, EC, Temperature, Total Alkalinity, TDS	Whole country	(Edirisinghe et al., 2018)
22	Groundwater fluoride in Sri Lanka: opportunities to mitigate the risk at maximum contaminant level	2018	GW	F	Whole country	(Ranasinghe et al., 2018)

No.	Journal Title	Year	Water Source	Water Quality Parameters	Geographical Locations	Citation
23	Assessment of Groundwater Quality in CKDu Affected Areas of Sri Lanka: Implications for Drinking Water Treatment	2019	GW	DOC	Anuradhapura district	(Cooray et al., 2019)
24	Drinking water and chronic kidney disease of unknown aetiology in Anuradhapura, Sri Lanka	2019	GW	Qualitative analysis	Anuradhapura district	(de Silva, 2019)
25	Urban Watercourses in Peril: Implications of Phthalic Acid Esters on Aquatic Ecosystems Caused by Urban Sprawl	2019	GW	Phthalic Acid Esters (PAEs)	Colombo district	(Jayaweera et al., 2019)
26	Drinking-Water Supply for CKDu Affected Areas of Sri Lanka, Using Nano-filtration Membrane Technology: From Laboratory to Practice	2019	GW	pH, EC, Total Alkalinity, Mg, Ca, Hardness, K, Na, Cl, F, Sulphate, Fe, and DOC	Polonnaruwa and Anuradhapura districts (North Central Province)	(Cooray et al., 2019)
27	Characterization of dissolved organic carbon in shallow groundwater of chronic kidney disease affected regions in Sri Lanka	2019	GW	DOC	Polonnaruwa and Anuradhapura districts (North Central Province)	(Makehelwala et al., 2019)
28	Assessment of groundwater quality using water quality index (WQI): A case study of a hard rock terrain in Sri Lanka	2020	GW	pH, EC, Alkalinity, Hardness, Cl, Phosphate, Sulphate, F, Na, K, Ca, Mg, Al, Cr, Mn, Fe, Co, Ni, Cu, Zn, As, Sr, Cd and Pb	Monaragala district	(Udeshani et al., 2020)
29	Assessment of spatial and temporal trend of groundwater salinity in Jaffna Peninsula and its link to paddy land abandonment	2020	GW	Salinity and EC	Jaffna district	(Gopalakrishnan et al., 2020)
30	Evaluation of groundwater quality and reverse osmosis water treatment plants in the endemic areas of Chronic Kidney Disease of Unknown Etiology (CKDu) in Sri Lanka	2020	GW	DOC, Na, K, Ca, Mg, NH, Cl, F, Br, Nitrite, Nitrate, Sulphate, and Phosphate	Anuradhapura district	(Imbulana et al., 2020)
31	Exploring the Root Cause for Chronic Kidney Disease of Unknown Etiology (CKDu) via Analysis of Metal Ion and Counterion Contaminants in Drinking Water: A Study in Sri Lanka	2020	GW	Cd, Pb, Cr, As, Zn, Cu, Ca, Mg, Na, K, Fe, Mn, Co, Ni, F, Phosphate and Nitrate	Polonnaruwa and Anuradhapura districts (North Central Province)	(Perera et al., 2020)
32	Geogenic fluoride and arsenic in groundwater of Sri Lanka and its implications to community health	2020	GW	F and As	Whole country	(Chandrajith et al., 2020)
33	Groundwater quality in the Jaffna peninsula of Sri Lanka and a qualitative study of BTEX	2020	GW	Nitrate, EC, Pb, Cd, TDS, pH and Turbidity	Jaffna district	(Ehanathan et al., 2020)

No.	Journal Title	Year	Water Source	Water Quality Parameters	Geographical Locations	Citation
	removal by greenly synthesized iron nanoparticles-electro-catalyst system					
34	Impact of water quality on Chronic Kidney Disease of unknown etiology (CKDu) in Thunukkai Division in Mullaitivu District, Sri Lanka	2020	GW	Turbidity, pH, EC, Salinity, TDS, Nitrate, F, Phosphate, Total Hardness, Mg, Ca, Na, K, Sulphate, Alkalinity, Cl, As, and Cd	Mullaitivu district	(Gobalarajah et al., 2020)
35	Influences of seawater intrusion and anthropogenic activities on shallow coastal aquifers in Sri Lanka: evidence from hydrogeochemical and stable isotope data	2020	GW	Temperature, pH, EC, DO, Eh, Bicarbonate, Cl, F, Br, Nitrate, Nitrite, Phosphate, Sulphate, F, Cl, Na, K, Ca, Mg, Sr, FE, Mn, Al, Ni, Cu, Zn, As, and Ba	Puttalam district	(Jayathunga et al., 2020)
36	Possible links between groundwater geochemistry and chronic kidney disease of unknown etiology (CKDu): an investigation from the Ginnoruwa region in Sri Lanka	2020	GW	Temperature, pH, EC, TDS, Alkalinity, Total Hardness, Cl, F, Nitrate, Sulphate, Phosphate, Na, K, Ca, Mg, Fe, Mn, Li, B, Sr, Ba, Al, Cr, Co, Ni, Cu, Zn, As, Cd, Pb	Badulla district	(Balasooriya et al., 2020)
37	Using water quality and isotope studies to inform research in chronic kidney disease of unknown aetiology endemic areas in Sri Lanka	2020	GW	DO, pH, EC, Ca, Mg, Na, K, F, Cl, Sulphate, Nitrate, Nitrite, Bicarbonate, CO_3 , As, Cu, Mn, Fe, Ni, Rb, Zn, Si and Sr	Polonnaruwa and Anuradhapura districts (North Central Province)	(Nikagolla et al., 2020)
38	Water quality and chronic kidney disease of unknown aetiology (CKDu) in the dry zone region of Sri Lanka: impacts on well-being of village communities and the way forward	2020	GW	Qualitative analysis	Whole country	(Pinto et al., 2020)
39	Ca^{2+}, and SO_4^{2-} Interactions with Dissolved Organic Matter: Implications of Groundwater Quality for CKDu Incidence in Sri Lanka	2020	GW	DOC, Ca and Sulphate	Polonnaruwa and Anuradhapura districts (North Central Province)	(Makehelwala et al., 2020)
40	Drinking Water and CKD of Unknown Etiology in Sri Lanka: A Community Perspective	2021	GW	Qualitative analysis	Polonnaruwa and Anuradhapura districts (North Central Province)	(de Silva, 2021)
41	Study of Evidences on CKDu Based on Environmental, Social, Economic and Health Patterns of Selected Population a Case Study Done at Wilgamuwa Area.	2020	GW	F, Cl, Br, Nitrate, Sulphate and Phosphate	Matale District	(Rajapakshe et al., 2020)

No.	Journal Title	Year	Water Source	Water Quality Parameters	Geographical Locations	Citation
42	Hardness and Fluoride Removal Efficiency of Groundwater: Use of Physiochemical Methods in Water Purification at Anuradhapura District Dry Zone in Sri Lanka	2020	GW	pH, EC, Turbidity, TDS, Alkalinity, Total Hardness, F and TOC	Anuradhapura district	(Kumara et al., 2020)
43	Geochemistry of Groundwater in the Uva Province, Sri Lanka-Implications for Chronic Kidney Disease of Uncertain Origin	2021	GW	pH, EC, TDS, Salt (NaCl), DO, Total Hardness, Bicarbonate, Nitrate, Phosphate, Sulphate, Na, Mg, K, Ca, Cl, and F	Badulla and Monaragala districts	(Piyathilake et al., 2021)
44	Groundwater as a potential cause of Chronic Kidney Disease of unknown etiology (CKDu) in Sri Lanka: a review	2021	GW	F, Total Hardness, Ca, Mg, Na, Al, Si, Cd, As, Pb, DOC and cyanotoxins	Whole country	(Imbulana and Oguma, 2021)
45	Influence of climate on groundwater fluoride in different climatic domains in a hard rock terrain of Sri Lanka: implications to community health	2021	GW	F	Ratnapura, Badulla, Moneragala and Hambantota districts (Walawe river basin)	(Senarathne et al., 2021a)
46	Natural and Anthropogenic Controls of Groundwater Quality in Sri Lanka: Implications for Chronic Kidney Disease of Unknown Etiology (CKDu)	2021	GW	Nitrate, Hardness, F, As, Cd, Fe, Mn, Cu, Cr, Pb and Al	Whole country	(Xu et al., 2021)
47	Seasonal Variations in Groundwater Quality and Hydrogeochemistry in the Endemic Areas of Chronic Kidney Disease of Unknown Etiology (CKDu) in Sri Lanka	2021	GW	pH, EC, TDS, Alkalinity, Ca, Mg, Na and Bicarbonate	Anuradhapura district	(Imbulana et al., 2021)
48	The Groundwater Geochemistry and the Human Health Risk Assessment of Drinking Water in an Area with a High Prevalence of Chronic Kidney Disease of Unknown Etiology (CKDu), Sri Lanka	2021	GW	pH, Temperature, DO, EC, Ca, Mg, Na, K, Cd, Cr, Pb, As, Cu, Fe, Mn, Zn, Al, F, Phosphate, Sulphate and Nitrate	Badulla district	(Botheju et al., 2021)
49	The influence of water-rock interactions on household well water in an area of high prevalence chronic kidney disease of unknown aetiology (CKDu)	2021	GW	Na, Mg, K, Ca, Sulphate and Bicarbonate	Anuradhapura district	(McDonough et al., 2021)
50	Water sources and kidney function: investigating chronic kidney disease of unknown etiology in a prospective study	2021	GW	Agrochemicals (Diazinon, p p'-DDE, propanil, Endosulfan II, o,p'-DDT), Hardness, and Phosphate	Matale district	(Vlahos et al., 2021)

No.	Journal Title	Year	Water Source	Water Quality Parameters	Geographical Locations	Citation
51	Hygiene risk of waterborne pathogenic viruses in rural communities using onsite sanitation systems and shallow dug wells	2021	GW	E coli and PMMoV (viral indicator of faecal contamination)	Galle district	(Otaki et al., 2021)
52	Assessment of Groundwater Quality in Sri Lanka Using Multivariate Statistical Techniques	2021	GW	F, EC, pH, Cl, Sulphate, Nitrate, Na, Mg, Ca, Al, Cr, Fe, Ni, Cu, Zn, As and Cd	Whole country	(Balasooriya et al., 2021)
53	Pharmaceutical contaminants in shallow groundwater and their implication to poor sanitation facilities in low-income countries	2021	GW	E coli, Total coliforms, nitrate, Cl, and 7 pharmaceuticals (acetaminophen, atenolol, caffeine, carbamazepine, cotinine, sulfamethoxazole, and sulphapyridine)	Galle District	(Do et al., 2021)
54	Profiles of antibiotic resistome and microbial community in groundwater of CKDu prevalence zones in Sri Lanka	2021	GW	Antibiotic Resistome	Anuradhapura, Monaragala and Kandy districts	(Cooray et al., 2021)
55	Risk Assessment of Trace Element Contamination in Drinking Water and Agricultural Soil: A Study in Selected Chronic Kidney Disease of Unknown Etiology (CKDu) Endemic Areas in Sri Lanka	2021	GW	F, Cl, Br, Nitrate, Phosphate, Sulphate, Cr, Mn, Fe, Co, As, Cd, Ca, Mg, Pb, Cu, Zn, Na, Al, K and Ni	Polonnaruwa and Anuradhapura districts (North Central Province)	(Perera et al., 2021)
56	Significance of Mg-hardness and fluoride in drinking water on chronic kidney disease of unknown etiology in Monaragala, Sri Lanka	2022	GW	pH, EC, F, Hardness, Na, K, Mg, Ca, Fe, Mn, Sr, Ba, Li, Al, V, Cr, Co, Ni, Cu, Zn, As, Se, Rb, Cd, and Pb	Monaragala district	(Liyanage et al., 2022)
57	Spatial and physicochemical assessment of groundwater quality in the urban coastal region of Sri Lanka	2022	GW	pH, EC, TDS, Cl, Mg, Ca, K, Na, Bicarbonate, and Turbidity	Puttalam district	(Perera et al., 2022)
58	The environmental pollution of Kandy Lake: A case study from Sri Lanka	1982	SW (Surface Water)	F, Cu, Nitrate, TP, Specific conductivity, pH and Faecal Coliform	Kandy district (Kandy lake)	(Dissanayake et al., 1982)
59	Fish uptake and levels of Cr, Cu, Cd, Ni, Pb and Zn in Beire Lake	2001	SW	Cr, Cu, Cd, Ni, Pb and Zn	Colombo district	(Wijesinghe et al., 2001)
60	Emergence of a Microcystis bloom in an urban water body, Kandy lake, Sri Lanka	2003	SW	*Microcystis aeruginosa bloom*	Kandy district (Kandy lake)	(Silva, 2003)
61	Occurrence of toxigenic cyanobacterial blooms in freshwaters of Sri Lanka	2006	SW	Cyanobacteria and other phytoplanktons, pH, TN and TP	17 reservoirs in Sri Lanka	(Jayatissa et al., 2006)
62	Occurrence and distribution of polycyclic	2007	SW	Polycyclic Aromatic Hydrocarbons (PAHs)	Colombo and Kalutara district	(Pathiratne et al., 2007)

No.	Journal Title	Year	Water Source	Water Quality Parameters	Geographical Locations	Citation
	aromatic hydrocarbons (PAHs) in Bolgoda and Beira Lakes, Sri Lanka				(Bolgoda and Beira Lakes)	
63	Occurrence of perfluorinated acids and fluorotelomers in waters from Sri Lanka	2007	SW	Perfluorinated compounds (PFCs), Perfluorooctane sulfonate (PFOS), and perfluorooctanoic acid (PFOA)	Colombo district	(Guruge et al., 2007)
64	Spatial and temporal changes of hydrogeochemistry in ancient tank cascade systems in Sri Lanka: evidence for a constructed wetland	2008	SW	pH, EC, Nitrate, Nitrite, Phosphate, Sulphates, Alkalinity, Cl, Na, K, Ca, Mg, Fe and Mn	Kurunegala district (Malagane in the Deduru Oya basin)	(Mahatantila et al., 2008)
65	Trace Metal Concentrations in Nile Tilapia (Oreochromis niloticus) in Three Catchments, Sri Lanka	2009	SW	As, Ca, Cd, Co, Cr, Cu, Fe, K, Mg, Mn, Na, Ni, P, Pb, Sr and Zn	Anuradhapura (Rajanganaya reservoir), Ratnapura (Udawalawe reservoir) Polonnaruwa (Kaudulla tank) districts	(Allinson et al., 2009)
66	Pollution of River Mahaweli and farmlands under irrigation by cadmium from agricultural inputs leading to a chronic renal failure epidemic among farmers in NCP, Sri Lanka	2011	SW	Cd	Nuwara Eliya district	(Bandara et al., 2011)
67	Water pollution due to a harmful algal bloom: a preliminary study from two drinking water reservoirs in Kandy, Sri Lanka	2011	SW	Phosphate, Nitrate, DO, pH, Dinoflagellate Species	Kandy district (Rosmith and Dunumadalawa reservoirs)	(Yatigammana et al., 2011)
68	Aquatic life health quality assessment of the Bolgoda canal and Waga stream with respect to selec ted physicochemical parameters and bioindicators	2012	SW	Macroinvertebrates, phytoplankton, Temperature, pH, EC, DO, TN and Orthophate, BOD, Alkalinity, Sulphide, and Hardness	Colombo district	(Idroos and Manage, 2012)
69	Monitoring and predicting eutrophication of Sri Lankan inland waters using ASTER satellite data	2014	SW	Chlorophyll-a, Nitrate and TP	Colombo and Kalutara districts (Bolgoda North Lake)	(Dahanayaka et al., 2014)
70	Multivariate analysis of physico-chemical and microbial parameters of surface water in Kelani river basin	2014	SW	Water Temperature, DO, EC, TDS, Salinity, COD, Total Hardness, Pd, Cd, Cr, Cu, Zn and Al	Colombo district	(Mahagamage et al., 2016)
71	Spatial and vertical distributions of sedimentary halogenated polycyclic aromatic hydrocarbons in moderately polluted areas of Asia	2015	SW	The sedimentary Halogenated (chlorinated and brominated) Polycyclic Aromatic Hydrocarbons (PAHs)	Colombo district	(Ohura et al., 2015)

No.	Journal Title	Year	Water Source	Water Quality Parameters	Geographical Locations	Citation
72	Impact of Land Use on Surface Water Quality: A Case Study in the Gin River Basin, Sri Lanka	2016	SW	Water Temperature, pH, DO, EC, Turbidity, BOD, Ammonia, Nitrate, Nitrite, Phosphate and SS	Galle district	(Amarathunga and Kazama, 2016)
73	Impacts of agricultural practices on water quality in Uma Oya catchment area in Sri Lanka	2016	SW	EC, pH, Temperature, BOD, TDS, TS, TSS, Nitrate, Nitrite, Ammonia, Phosphate, Sulphate, F, Mg and Na	Badulla district	(Gunawardhana et al., 2015)
74	Spatial and temporal distribution of cyanobacteria in Batticaloa Lagoon	2016	SW	Cyanobacteria, Nitrate, Turbidity and TP	Batticaloa district	(Harris et al., 2016)
75	Occurrence, fate and ecological risk of antibiotics in Hospital effluent water and sediments in Sri Lanka	2016	SW	Four antibiotics classes (sulfanomides, penicillin, tetracycline and macrolids)	Whole country	(Liyanage and Manage, 2016)
76	Assessment of water quality and trophic state in Sooriyawewa reservoir in the Southern province of Sri Lanka.	2017	SW	Chlorophyll-a, Nitrate, Phosphate, SS, EC and Temperature	Hambantota district	(Wijewardene et al., 2017)
77	Impact of Population Growth on the Water Quality of Natural Water Bodies	2017	SW	BOD, DO and Total Coliform	Colombo district	(Liyanage and Yamada, 2017)
78	Industrial pollution and the management of river water quality: a model of Kelani River, Sri Lanka	2017	SW	pH, EC, Turbidity, DO, COD, BOD, Cl, Faecal Coliform, Total Coliform, Nitrate and Phosphate	Colombo district	(Gunawardena et al., 2017)
79	Land use impacts on river health of Uma Oya, Sri Lanka: implications of spatial scales	2017	SW	pH, EC, Temperature, DO, TDS, TS, TSS, Nitrate, Nitrite, Phosphate, Sulphate and Ammonia	Badulla district	(Jayawardana et al., 2017)
80	Spatial distribution, enrichment, and source of environmentally important elements in Batticaloa lagoon, Sri Lanka	2017	SW	As, Cr, Cu, Fe, Nb, Ni, Pb, Sc, Sr, Th, V, Y, Zn, and Zr	Batticaloa district	(Adikaram et al., 2017)
81	Seasonal and tidal influence for water quality changes in coastal Bolgoda Lake system, Sri Lanka	2018	SW	EC, pH, Temperature, DO and Eh	Colombo and Kalutara districts (Bolgoda North Lake)	(Ratnayake et al., 2018)
82	Stresses over surface water sources in a human dominated environment: A case study in Hamilton canal, Sri Lanka	2018	SW	EC, Salinity, pH, Turbidity, Total Alkalinity, Total Hardness, TDS, F, Na, Mg, Ca, K, Fe, Cl, Phosphate, Sulphate, Pb, and Cd	Puttalam and Colombo districts	(Chandrasekara et al., 2018)
83	Water Quality Modelling in Kelani River Downstream	2018	SW	DO, BOD, COD, TN and TP	Colombo district	(Kehelella et al., 2018)
84	Occurrence of trihalomethane (THM) in relation to treatment technologies and water	2018	SW	Total trihalomethanes (THMs)	Kandy district	(Amarasooriya et al., 2018)

No.	Journal Title	Year	Water Source	Water Quality Parameters	Geographical Locations	Citation
	quality under tropical conditions					
85	Community engagement and pollution mitigation at Kandy Lake, Sri Lanka	2018	SW	Qualitative analysis	Kandy district	(Jinadasa et al., 2018)
86	Assessment of Pollution Sources, Fate of Pollutants, and Potential Instream Interventions to Mitigate Pollution of Earthen Canals of Urban to Rural-Urban Fringe	2019	SW	DO, pH, EC, TS, TSS, TDS, Temperature, Turbidity, Nitrite, Nitrate, Ammonia and Soluble Reactive Phosphorus	Colombo district	(Gomes et al., 2019)
87	Distributed modelling of water resources and pollute transport in Malwathu Oya Basin, Sri Lanka	2019	SW	TN, TP, Dissolved Nitrogen, Dissolved Phosphorus, Particulate and Phosphorus	Mullaitivu district	(Dahanayake and Rajapakse, 2019)
88	Influence of hydrology on water quality and trophic state of irrigation reservoirs in Sri Lanka	2019	SW	Alkalinity, Chlorophyll-a, DO, EC, Nitrate, pH, Temperature and TP	Whole country	(Nadarajah et al., 2019)
89	Seasonal and Spatial Variation of Dissolved Oxygen and Nutrients in Padaviya Reservoir, Sri Lanka	2019	SW	DO, pH, Reactive Phosphate, Nitrite, Nitrate and Ammonia	Anuradhapura district	(Siriwardana et al., 2019)
90	Plastics in surface water of southern coastal belt of Sri Lanka (Northern Indian Ocean): Distribution and characterization by FTIR	2020	SW	Microplastics	Galle, Matara and Hambantota districts.	(Athapaththu et al., 2020)
91	Seasonality impels the antibiotic resistance in Kelani River of the emerging economy of Sri Lanka	2020	SW	Antibiotics (resistance for norfloxacin, ciprofloxacin, levofloxacin, kanamycin monosulfate, tetracycline, and sulfamethoxazole)	Colombo district	(Kumar et al., 2020)
92	Toxic hazards of industrial waste receiving canal system in the lower catchment of Kelani River basin, Sri Lanka	2020	SW	pH, Temperature, DO, TDS, BOD, COD, Nitrate, Phosphate, Cd, Pb, Cu, Cr, Oil and Grease	Colombo district	(Kuruppuarachchi and Pathiratne, 2020)
93	Prevalence of antibiotic resistance in the tropical rivers of Sri Lanka and India	2020	SW	E coli and antibiotics (levofloxacin, ciprofloxacin, norfloxacin, kanamycin monosulphate, sulfamethoxazole, and tetracycline)	Colombo and Galle districts	(Kumar et al., 2020b)
94	Potential for Formation of Trihalomethane in Diverted and Non-Diverted Areas of Mahaweli River in Sri Lanka.	2020	SW	Total trihalomethanes (THMs)	Nuwara Eliya district	(Bandara et al., 2020)
95	Assessment of current status and geospatial analysis of compliance to bacteriological parameters: Tools to	2021	SW	Total Coliform, Thermotolerant Coliform	Gampaha, Colombo and Kalutara districts	(Wijesekara et al., 2021)

No.	Journal Title	Year	Water Source	Water Quality Parameters	Geographical Locations	Citation
	ensure access to safe water in Sri Lanka					
96	Effects of hydrological regimes and limnological parameters on plankton community properties in tropical irrigation reservoirs: A Sri Lankan case study	2021	SW	Turbidity, pH, Conductivity, Temperature, Alkalinity, Chlorophyll-a, Dissolved Phosphorus, DO, Nitrate, TP, Phytoplankton and Zooplankton	Kurunegala, Puttalam, Polonnaruwa and Anuradhapura districts (10 reservoirs)	(Weerakoon et al., 2021)
97	Microplastic pollution in Marine Protected Areas of Southern Sri Lanka	2021	SW	Microplastics	Hambantota and Galle districts	(Dharmadasa et al., 2021)
98	Modified, optimized method of determination of Tributyltin (TBT) contamination in coastal water, sediment and biota in Sri Lanka	2021	SW	Tributyltin (TBT)	Colombo, Gampaha, Kalutara, Galle, Matara, Hambantota districts.	(Bandara et al., 2021)
99	Assessing Potential Environmental Impacts of Pesticide Usage in Paddy Ecosystems: A Case Study in the Deduru Oya River Basin, Sri Lanka	2022	SW	Pesticides (Diazinon, Fipronil, Fenobucarb, Chlorantraniliprole, Thiamethonxam, Oxyfluorfen, Etofenprox, Tebuconazol, Captan)	Kandy, Matale and Kurunegala districts (Deduru Oya river)	(Jayasiri et al., 2022b)
100	Heavy metal pollution in selected upland tributaries of Sri Lanka: comprehension towards the localization of sources of pollution	2022	SW	pH, EC, TDS, Pb, Cd, As and Hg	Nuwara Eliya, Kandy, Matale, Kurunegala and Galle districts	(Kodikara et al., 2022)
101	Spatio-temporal analysis of water quality for pesticides and other agricultural pollutants in Deduru Oya river basin of Sri Lanka	2022	SW	**Pesticides - Herbicides** (MCPA, pretilachlor, bispyribac sodium, propanil, glyphosate, fenoxaprop-p-ethyl, oxyfluorfen, cyhalofopbutyl) **Pesticides - Insecticides** (thiamethoxam, chlorantraniliprole, fenobucarb, carbosulfan, fipronil, diazinon, carbofuran, etofenprox) **Pesticides - Fungicides** (carbendazim, hexaconazole, tebuconazole, captan) **Heavy metals** (As, Cd, Pb) **Minerals** (Ammoniacal Nitrogen, Nitrite, Nitrate), Phosphate, Sulphate, Mg, Ca, Na) **Physico-chemical properties** (COD, pH, EC, DO, Turbidity)	Kandy, Matale and Kurunegala districts (Deduru Oya river)	(Jayasiri et al., 2022a)

No.	Journal Title	Year	Water Source	Water Quality Parameters	Geographical Locations	Citation
102	Environmental pollution in Sri Lanka: A review.	2000	GW, SW	DO, COD, BOD, Total Coliform, TN, TP, Zn, Cd and Pb	Whole country	(Ileperuma, 2000)
103	Human impacts and the status of water quality in the Bundala RAMSAR wetland lagoon system in Southern Sri Lanka	2004	GW, SW	EC, pH, Nitrate, Ammonia, TN, Total Reactive Phosphorus and TP	Hambantota district (Bundala National park)	(Piyankarage et al., 2004)
104	Fate of phosphate and nitrate in waters of an intensive agricultural area in the dry zone of Sri Lanka	2010	GW, SW	Nitrate, Phosphate	Anuradhapura (Kala Oya basin),	(Young et al., 2010)
105	Geochemical characteristics of groundwater in different climatic zones of Sri Lanka	2015	GW, SW	Na, K, Ca, Mg, Fe, Mn, Cu, As and Cd	Whole country	(Rubasinghe et al., 2015)
106	Contamination status of salmonella spp., Shigella spp. and campylobacter spp. in surface and groundwater of the Kelani River Basin, Sri Lanka	2020	GW, SW	Total Coliform, E. coli, *Salmonella spp.*, *Shigella spp.* and *Campylobacter spp.*	Colombo district	(Mahagamage et al., 2020)
107	Quality of surface and ground waters for domestic and irrigation purposes in CKD/CKDu prevalent areas in Moneragala District, Sri Lanka	2020	GW, SW	pH, DO, TDS, EC, Nitrate, Phosphate, Sulphate, F, Cl, Hardness, Na, Mg, K and Ca	Moneragala district	(Dissanayake et al., 2020)
108	Evaluation of Water Quality of Community Managed Water Supply Schemes in Galle District	2020	GW, SW	Temperature, Turbidity, EC, pH, F, Fe, TN, Total Hardness, Mn, Cd, As, Pb, E coli and Total Coliforms	Galle District	(Shayan et al., 2020)
109	Influence of regional climatic on the hydrogeochemistry of a tropical river basin-a study from the Walawe river basin of Sri Lanka	2021	GW, SW	pH, EC, TDS, total hardness, HCO_3^- , Cl, F, sulphate, Nitrate-N, Nitrite, phosphate, Na, K, Ca, Mg	Ratnapura, Badulla, Moneragala and Hambantota districts (Walawe river basin)	(Senarathne et al., 2021b)
110	Prevalence and Quantitative Analysis of Antibiotic Resistance Genes (ARGs) in Surface and Groundwater in Meandering Part of the Kelani River Basin in Sri Lanka	2021	GW, SW	Antibiotics (Tetracycline, Penicillin)	Colombo district	(Liyanage et al., 2021)
111	Fluoride occurrences, health problems, detection, and remediation methods for drinking water: A comprehensive review	2022	GW, SW	F	Whole country	(Solanki et al., 2022)
112	Metal release from serpentine soils in Sri Lanka	2014	Soil	Ni, Mn and Cr	Hambantota, Ratnapura, Monaragala districts	(Vithanage et al., 2014)
113	Building resilience on water quality management through	2018	WR (Water Resources)	BOD, Ammonia, Nitrate, Nitrate, TP, TSS, TDS and Cl	Whole country	(Wickramasinghe et al., 2017)

No.	Journal Title	Year	Water Source	Water Quality Parameters	Geographical Locations	Citation
	grey water footprint approach: a case study from Sri Lanka					
114	Policy choice and riverine water quality in developing countries: An integrated hydro-economic modelling approach	2018	WR	DO and BOD	Colombo district	(Gunawardena et al., 2018)
115	Applicability of semi quantitative approach to assess the potential environmental risks for sustainable implementation of water supply schemes: a case study of Sri Lanka	2021	WR	Colour, Turbidity, pH, TDS, Total Alkalinity, Hardness, Nitrate, Fe, Total Coliform and E coli	Kegalle District	(Bellanthudawa et al., 2021)
116	Treatment enhances the prevalence of antibiotic-resistant bacteria and antibiotic resistance genes in the wastewater of Sri Lanka, and India	2020	WW (wastewater), SW	E coli and antibiotics (Fluoroquinolone, β-lactams, and sulphonamides)	Colombo district	(Kumar et al., 2020a)

Abbreviations used in this table:

Al: Aluminium, As: Arsenic, B: Boron, Ba: Barium, Br: Bromine, Ca: Calcium, Cd: Cadmium, Cl: Chloride, Co: Cobalt, Cr:Chromium, Cu: Copper, F: Fluoride, Fe: Iron, Hg: Mercury, K: Potassium, Li: Lithium, Mg: Magnesium, Mn: Manganese, Mo: Molybdenum, Na: Sodium, Nb: Niobium, Ni: Nickel, Pb: Lead, Rb: Rubidium, Sc: Scandium, Se: Selenium, Si: Silicon, Sr: Strontium, Th: Thorium, U: Uranium, V: Vanadium, Y: Yttrium, Zn: Zinc, Zr: Zirconium

EC: Electrical Conductivity, TDS: Total Dissolved Solids, DO: Dissolved Oxygen, DOC: Dissolved Organic Carbon, TS: Total Solids, COD: Chemical Oxygen Demand, BOD: Biological Oxygen Demand, TP: Total Phosphorus, TN: Total Nitrogen, Eh: Oxidation-reduction potential, TOC: Total Organic Carbon, SS: Suspended Solids/Sediment

Table S3 The ten large river basins in Sri Lanka, their population and annual rainfall.

River Basin	Area (km^2)	Ranking (Top 10)	Population	Annual Rainfall (mm)
Mahaweli Ganga	10606	1	3,504,473	1921
Malwathu Oya (Aruvi Aru)	3448	2	468,718	1359
Kala Oya	2805	3	575,744	1358
Deduru Oya	2766	4	1,051,811	1734
Kalu Ganga	2631	5	1,028,714	3990
Walawe Ganga	2529	6	577,988	2034
Kelani Ganga	2298	7	2,129,173	3487
Yan Oya	2045	8	159,043	1560
Gal Oya	1866	9	331,441	1826
Mi Oya	1528	10	199,485	1285

Table S4. Mean surface water-quality parameters measured (mean period: 2003 to 2016) along the course of River Kelani (CEA, 2019).

Location	pH	EC	TURB	TEMP	DO	COD	BOD5	Cl	Cr	Pb	NO3	PO4	Total Coli	Faecal coli
Unit		mS/cm	NTU	deg. C	mg/L	mg/L	mg/L	mg/L	µg/L	µg/L	mg/L	mg/L	MPN/100ml	MPN/100ml
Sri Lankan Standards	6.5 to 8.5	0.7*	5.00		6.00	3.00	10.00	250.00	50.00	50.00	10.00	0.70	10000†	1000†
Eswathu Oya	6.53	0.03	9.06	26.71	6.55	9.77	1.94	8.11	1.24	0.97	0.43	0.05	8072	NA
Hanwella Bridge	6.97	0.04	56.94	26.79	7.07	10.17	1.59	6.61	0.61	0.59	0.28	0.07	5790	1956
Japanese Friendship Bridge	7.10	1.72	92.96	27.30	6.55	16.27	1.69	413.65	0.34	0.47	0.45	0.07	14576	7191
Kaduwela Bridge	7.17	0.06	48.83	27.04	7.03	9.95	1.43	8.63	0.69	0.47	0.30	0.09	7441	1667
Kolonnawa Ela	7.17	2.66	97.00	28.97	1.80	59.50	14.33	388.33	0.01	0.01	0.42	0.25	16000	13667
Maha Ela	6.73	0.11	40.62	27.96	4.49	18.23	4.26	14.04	0.71	0.70	0.33	0.08	8600	2200
New Bridge Peliyagoda	7.04	1.76	31.63	27.73	5.99	25.91	3.16	667.59	1.12	1.07	0.53	0.07	10490	NA
Pugoda Ela	6.86	0.17	62.17	27.08	6.19	15.50	1.91	12.98	0.77	0.62	0.38	0.10	8011	2695
Pugoda Ferry	7.11	0.05	48.86	26.77	7.21	9.60	1.59	6.29	0.51	0.45	0.29	0.09	6361	2400
Pusseli Oya	6.78	0.04	67.24	27.41	6.58	13.19	1.84	8.72	0.72	0.59	0.83	0.08	6963	1929
Raggahawatte Ela	6.93	0.50	63.30	27.69	4.36	26.09	4.64	40.12	1.16	0.82	1.15	0.24	9472	3368
Seethawake Ferry	6.95	0.21	63.61	28.80	6.52	12.15	2.07	11.93	0.79	0.60	0.48	0.12	6453	2411
Thalduwa Bridge	7.09	0.03	43.57	27.06	7.03	10.48	1.61	5.98	0.71	0.58	0.29	0.06	6148	2051
Victoria Bridge	7.01	1.94	37.67	27.43	6.22	18.97	2.51	547.67	2.64	0.70	0.42	0.14	11936	4749
Wak Oya	6.89	0.03	37.12	27.13	6.81	10.36	1.60	6.43	0.74	0.59	0.29	0.07	6893	2190
Welivita Bridge	7.17	0.06	41.44	27.15	6.93	10.64	1.80	10.38	0.70	0.63	0.33	0.09	6486	2526
**Basin average**	**6.97**	**0.59**	**52.63**	**27.44**	**6.08**	**17.30**	**3.00**	**134.84**	**0.84**	**0.62**	**0.45**	**0.11**	**8731**	**3643**

* Category D shall be water source that require to undergo general treatment process, for drinking
† Category A shall be water that requires simple treatment, for drinking

Table S5 Number of groundwater samples in each district (*n*=25) within Sri Lanka. data source: National Water Supply and Drainage Board (NWSDB).

Name of district	No of sample	Name of district	No of sample
Ampara	40	Kurunegala	51
Anuradhapura	62	Mannar	15
Badulla	34	Matale	42
Batticaloa	23	Matara	8
Colombo	9	Monaragala	41
Galle	13	Mullaitivu	21
Gampaha	29	Nuwara Eliya	26
Hambantota	45	Polonnaruwa	47
Jaffna	3	Puttalam	29
Kalutara	26	Ratnapura	29
Kandy	16	Trincomalee	17
Kegalle	20	Vavuniya	25
Kilinochchi	17	**National total:**	**688**

Table S6. Mean groundwater quality parameters within each district in Sri Lanka from the NWSDB database.

District	Well Depth	Yield	GWL Depth	pH	Chloride	Alkalinity	Nitrate	Nitrite	Fluoride	Phosphate	TDS	Hardness	Iron	Sulphate
Ampara	40.13	76.24	8.69	7.39	183.00	168.28	1.24	0.09	1.14	0.88	663.44	196.84	6.72	57.82
Anuradhapura	48.51	65.71	10.62	7.65	164.32	259.72	3.14	0.02	1.16	0.55	617.15	287.74	4.73	46.00
Badulla	38.00	67.56	12.84	7.54	16.10	174.77	1.48	0.02	0.68	1.06	206.53	143.77	4.17	12.04
Batticaloa	43.13	66.55	10.93	7.43	211.86	202.68	3.75	0.20	0.88	1.71	1564.14	198.77	1.66	63.68
Colombo	55.09	140.11	19.31	7.19	23.22	76.50	1.96	0.02	0.48	0.08	160.00	64.00	1.96	3.00
Galle	47.54	405.42	13.53	7.48	21.92	85.62	1.85	0.01	0.47	0.04	156.83	95.46	1.53	12.50
Gampaha	45.39	82.25	17.16	7.43	25.79	103.10	2.39	0.03	0.50	0.08	147.50	121.50	3.10	15.08
Hambantota	36.82	106.49	14.69	7.47	374.11	260.33	1.33	NA	1.40	NA	1387.13	465.24	1.19	125.66
Jaffna	10.67	173.33	7.00	7.34	37.67	198.67	1.77	0.01	0.31	0.87	324.00	192.00	0.06	7.33
Kalutara	44.19	59.96	10.96	7.29	14.92	72.08	0.56	0.00	0.60	0.11	114.83	64.12	2.87	4.67
Kandy	59.13	109.20	14.46	7.42	118.25	171.21	7.68	0.25	0.75	0.82	401.00	255.63	3.14	33.70
Kegalle	56.79	413.53	16.30	7.57	208.70	192.84	0.90	0.00	0.59	NA	186.11	244.50	1.53	39.77
Kilinochchi	30.12	455.82	13.12	7.59	298.54	248.50	0.49	0.01	0.47	0.29	394.00	223.75	0.15	32.81
Kurunegala	44.50	34.42	12.58	7.06	372.09	268.04	1.95	0.17	1.10	0.35	856.27	491.32	3.01	48.30
Mannar	31.07	531.53	17.80	7.64	229.07	321.27	1.80	0.03	0.33	0.61	840.20	277.33	0.48	28.73
Matale	43.41	48.06	12.80	6.53	54.89	205.09	3.10	0.46	0.55	0.23	337.75	195.81	1.62	9.19
Matara	48.88	927.25	21.69	7.13	24.88	113.00	0.05	0.02	0.58	0.31	185.50	124.75	2.28	9.29
Monaragala	44.90	95.09	8.49	7.79	57.83	225.78	1.12	0.25	0.83	0.59	396.22	207.61	3.02	49.14
Mullaitivu	41.93	221.20	20.50	7.40	336.10	254.38	2.84	0.08	0.46	0.95	1288.55	370.62	0.27	29.05
Nuwara Eliya	47.61	106.50	21.80	6.29	15.18	73.42	6.01	3.11	0.31	0.49	146.00	72.50	2.53	5.60
Polonnaruwa	46.55	90.56	10.34	6.98	76.52	231.09	4.02	0.02	1.20	0.69	405.59	233.05	1.56	21.16
Puttalam	43.47	125.11	16.30	7.36	676.71	241.71	1.37	0.10	0.42	0.04	819.58	560.86	1.85	56.24
Ratnapura	45.48	237.33	20.28	7.40	42.55	204.45	1.46	0.01	1.37	0.06	325.48	209.61	1.80	31.23
Trincomalee	37.25	147.65	9.88	7.45	500.71	283.41	0.84	0.06	0.41	0.52	701.00	555.29	4.53	48.93
Vavuniya	37.95	79.12	9.85	7.62	164.09	335.55	4.78	0.02	1.05	0.71	782.94	329.67	1.34	23.31
National	42.74	194.64	14.08	7.34	169.96	198.86	2.32	0.21	0.72	0.52	536.31	247.27	2.28	32.57

Table S7. Mean groundwater quality parameters within each aquifer type in Sri Lanka from the NWSDB database.

Aquifer	Well Depth	Yield	GWL Depth	pH	Chloride	Alkalinity	Nitrate	Nitrite	Fluoride	Phosphate	TDS	Hardness	Iron	Sulphate
Basement regolith aquifer	44.67	151.65	15.28	7.22	175.80	197.55	3.12	0.65	0.85	0.77	621.89	231.66	2.54	39.19
Deep confined aquifer	28.75	587.09	17.87	7.45	438.26	272.91	0.95	0.03	0.25	0.42	596.43	389.29	0.36	50.48
Laterite (cabook) aquifer	45.31	70.26	13.47	7.38	54.44	93.74	1.71	0.01	0.48	0.21	214.21	125.18	2.41	14.45
Regolith or fractured aquifer	44.21	74.93	9.79	7.55	164.85	259.85	2.66	0.10	1.04	0.59	523.85	302.75	3.38	36.21
Shallow alluvial aquifer	40.21	92.66	12.15	7.22	327.46	201.69	1.59	0.06	0.91	0.63	1033.69	400.97	1.98	80.72
Shallow karstic aquifer	12.00	400.00	1.00	6.61	41.00	192.00	2.70	0.02	0.57	2.45	324.00	220.00	0.03	20.00
Shallow sandy aquifer	46.35	225.76	22.38	7.22	417.08	108.46	3.45	0.05	0.52	0.24	279.00	317.50	3.74	43.33

Table S8. Mean groundwater quality parameters within each climate zone in Sri Lanka from the NWSDB database.

Climate	Well Depth	Yield	GWL Depth	pH	Chloride	Alkalinity	Nitrate	Nitrite	Fluoride	Phosphate	TDS	Hardness	Iron	Sulphate
Dry zone	41.55	125.82	11.61	7.42	279.16	262.88	2.54	0.08	1.04	0.74	875.28	343.15	2.28	54.40
Intermediate zone	42.64	91.57	14.56	7.22	129.03	193.21	2.85	0.49	0.71	0.55	436.24	234.44	3.32	32.01
Wet zone	49.93	223.09	17.01	7.24	60.20	104.85	2.75	1.34	0.52	0.37	178.74	127.26	2.63	18.43

Supplementary references:

CEA, (2019) Ambient water quality standards of Sri Lanka. The Gazette of the Democratic Socialist Republic of Sri Lanka, Central Environmental Authority, Colombo.

Cooray, P.G. (1984) The geology of Sri Lanka (Ceylon), 2nd ed. National Museums of Sri Lanka, Colombo.

Cooray, P.G. (1994) The Precambrian of Sri Lanka: a historical review. Precambrian Research 66, 3-18.

Dissanayake, C.B., Chandrajith, R., (2018) The Hydrogeological and Geochemical Characteristics of Groundwater of Sri Lanka, in: Mukherjee, A. (Ed.), Groundwater of South Asia. Springer Hydrogeology, Singapore, pp. 405-428.

Dissanayake, C.B., Weerasooriya, S.V.R., (1985) Hydrogeochemical atlas of Sri Lanka. Natural Resources. Energy & Science Authority of Sri Lanka, Colombo.

Fan, M., (2015) Sri Lanka's water supply and sanitation sector: Achievements and a way forward, ADB South Asia working paper series. Asian Development Bank (ADB), Manila, Philippines.

Herath, H.M.A.S., Kubota, K., Kawakami, T., Nagasawa, S., Motoyama, A., Weragoda, S.K., Chaminda, G.G.T. and Yatigammana, S.K. (2017) Potential risk of drinking water to human health in Sri Lanka. Environmental Forensics 18, 241-250.

Indika, S., Wei, Y., Cooray, T., Ritigala, T., Jinadasa, K.B.S.N., Weragoda, S.K., Weerasooriya, R. (2022) Groundwater-based drinking water supply in Sri Lanka: status and perspectives. Water 14, 1428.

Jayakody, P.M. (2015) The Influence of La Nina on Sri Lanka Rainfall. Sri Lanka Journal of Meteorology 1, 41-49.

Karuratne, R.N., (2007) Ground water - National Atlas of Sri Lanka. Survey Department of Sri Lanka, Colombo, pp. 66-67.

Lees, D.J., Gunatilake, J., (2017) The Hydrogeology of the Central Highlands in Sri Lanka and its effect on tunnel construction, 16th Australasian Tunnelling Conference 2017. Engineers Australia and Australasian Tunnelling Society.

Makubura, R., et al. (2022) A Simplified Mathematical Formulation for Water Quality Index (WQI): A Case Study in the Kelani River Basin, Sri Lanka. Fluids MDPI, 7, 147.

Panabokke, C.R., (2007) Groundwater Conditions in Sri Lanka – A geomorphic perspective. National Science Foundation of Sri Lanka, Colombo.

Panabokke, C.R., Perera, A.P.G.R.L., (2005) Groundwater Resources of Sri Lanka. Water Resources Board, Colombo, Sri Lanka, p. 28.

Panagos, P., Jones, A., Bosco, C., Kumar, P.S.S. (2011) European digital archive on soil maps (EuDASM). International Journal of Digital Earth 4, 434-443.

Premanath, L., (2021) Sri Lanka water quality study. The World Bank, Washington D.C., p. 71.

SLS 614, (2013) Sri Lankan Standards for potable water. Sri Lanka Standards Institution, Colombo, Sri Lanka.

Villholth, K.G., Rajasooriyar, L.D. (2010) Groundwater resources and management challenges in Sri Lanka–An overview. Water resources management 24, 1489-1513.

Zubair, L., Siriwardhana, M., Chandimala, J., Yahiya, Z. (2008) Predictability of Sri Lankan rainfall based on ENSO. International Journal of Climatology 28, 91-101.
